# Thyroid dysfunction in MASLD: Results of a nationwide study

**DOI:** 10.1016/j.jhepr.2025.101369

**Published:** 2025-02-26

**Authors:** Shuai Yuan, Fahim Ebrahimi, David Bergman, Marijana Vujković, Eleonora Scorletti, Xixin Ruan, Jie Chen, Hannes Hagström, Jonas F. Ludvigsson

**Affiliations:** 1 Unit of Cardiovascular and Nutritional Epidemiology, Institute of Environmental Medicine, Karolinska Institutet, Stockholm, Sweden; 2 Department of Surgery, Perelman School of Medicine at the University of Pennsylvania, PA, USA; 3 Corporal Michael J. Crescenz VA Medical Center, Philadelphia, PA, USA; 4 Department of Medical Epidemiology and Biostatistics, Karolinska Institutet, Stockholm, Sweden; 5 Department of Gastroenterology and Hepatology, Clarunis University Center for Gastrointestinal and Liver Diseases, Basel, Switzerland; 6 Department of Medicine, University of Pennsylvania Perelman School of Medicine, Philadelphia, PA, USA; 7 School of Human Development and Health, Faculty of Medicine, University of Southampton, Southampton, UK; 8 National Institute for Health Research Southampton Biomedical Research Centre, University of Southampton and University Hospital Southampton National Health Service Foundation Trust, Southampton, UK; 9 Department of Genetics, Perelman School of Medicine, University of Pennsylvania, Philadelphia, PA, USA; 10 Department of Gastroenterology, Central South University Third Xiangya Hospital, Changsha, Hunan, China; 11 Division of Hepatology, Department of Upper GI, Karolinska University Hospital, Stockholm, Sweden; 12 Department of Medicine, Karolinska Institutet, Stockholm, Sweden; 13 Department of Pediatrics, Örebro University Hospital, Örebro, Sweden; 14 Division of Digestive and Liver Disease, Department of Medicine, Columbia University Medical Center, New York, NY, USA

**Keywords:** Hyperthyroidism, Hypothyroidism, Liver disease

## Abstract

**Background & Aims:**

Thyroid hormones are known to be potent modulators of hepatic metabolism and targeting the thyroid hormone receptor was recently approved as the first treatment for metabolic-associated steatotic liver disease (MASLD); however, the exact relationship between thyroid disorders and biopsy-confirmed MASLD remains unclear.

**Methods:**

We conducted a nationwide matched case–control study leveraging data from the Swedish Epidemiology Strengthened by histoPathology Reports in Sweden (ESPRESSO) cohort, which includes liver biopsy data spanning from 1969 to 2017. We identified 12,172 patients with MASLD and 56,831 matched general-population controls, including 5,478 patients with MASLD with 10,682 sibling controls. Conditional logistic regression was used to calculate odds ratios for hypothyroidism and hyperthyroidism defined through ICD codes or prescription records. Causal inference was examined using Mendelian randomization (MR). Both observational and MR mediation analyses were performed to explore the roles of metabolic features.

**Results:**

Hypothyroidism was associated with 1.68-fold increased odds of MASLD (95% CI 1.36–2.06). The association remained stable in the analysis using siblings as controls. However, in absolute terms, hypothyroidism was uncommon and seen in 2.5% in people with MASLD and in 1.4% of controls. Higher genetically predicted thyroid-stimulating hormone levels and hypothyroidism were linked to increased MASLD risk. Mediation analysis showed that metabolic disorders contributed ∼41% to this risk. Furthermore, there was an inverse association between hyperthyroidism and MASLD (adjusted odds ratio 0.17, 95% CI 0.05–0.56); however, the association did not reach statistical significance in the MR analysis.

**Conclusions:**

The findings suggest that hypothyroidism is associated with a heightened risk of MASLD and that hyperthyroidism is potentially protective against MASLD.

**Impact and implications:**

The approval by the US FDA of resmetirom, a thyroid hormone receptor β-selective agonist for non-cirrhotic metabolic dysfunction-associated steatohepatitis with stage 2–3 fibrosis, highlights the potential role of thyroid dysfunction in metabolic-associated steatotic liver disease (MASLD). This study identified hypothyroidism as a risk factor for MASLD, especially in men and individuals younger than 40 years, with the association peaking at non-cirrhotic fibrosis. Metabolic disorders mediated ∼41% of the hypothyroidism–MASLD association. Hyperthyroidism was potentially inversely associated with MASLD. Despite its low prevalence (2.5% in MASLD cases, 1.4% in controls), the population health impact of hypothyroidism warrants further attention.

## Highlights

• Hypothyroidism raises MASLD odds by 1.68-fold, with stable sibling-based results.
• In terms of histological types, hypothyroidism–MASLD risk peaks in non-cirrhotic fibrosis.
• Metabolic disorders mediate ∼41% of the hypothyroidism–MASLD association.
• Genetically predicted thyroid-stimulating hormone levels and hypothyroidism are linked to higher MASLD risk.
• Hyperthyroidism shows a potential inverse association with MASLD.

## Graphical abstract

**Figure:**
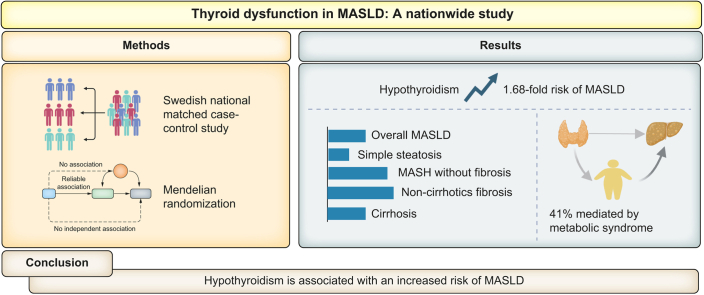

## Introduction

Metabolic-associated steatotic liver disease (MASLD) has become a major global health issue, affecting ∼38% of adults worldwide.^1^ Over the past three decades, the prevalence of MASLD has risen sharply,^2^ but treatment options remain limited despite this growing burden. However, in March 2024, the US FDA approved the first drug for MASLD, resmetirom, which is an oral, liver-directed, thyroid hormone receptor β-selective agonist.^3^ This approval, specifically for patients with non-cirrhotic metabolic dysfunction-associated steatohepatitis (MASH) and stage 2–3 fibrosis, underscores the potential role of thyroid dysfunction in MASLD pathogenesis.

Impaired thyroid function is known to alter hepatic metabolism by impacting lipid metabolism, fatty acid oxidation, and energy production.^4^ Epidemiological studies have also explored the relationship between hypothyroidism and MASLD, with meta-analyses generally indicating a positive association.^5^^,^^6^ However, the causal role of hypothyroidism in MASLD development remains unclear as a result of several limitations: (1) significant heterogeneity of diagnostic modalities for MASLD and missing information on liver histology; (2) predominance of cross-sectional study designs; (3) small sample sizes; and (4) potential residual confounding resulting from inadequate adjustment for key variables, such as metabolic disorders and autoimmune diseases. Furthermore, the role of sex differences in this association has not been fully investigated.^7^ To strengthen causal inference, Mendelian randomization (MR) has been proposed as a valuable approach, using genetic variants as instrumental variables to reduce confounding and reverse causation.^8^ In terms of the association between hyperthyroidism and MASLD, few studies have been conducted,^9^^,^^10^ and often only with a small sample size.

The increasing prevalence of MASLD is linked to metabolic risk factors.^11^ Hypothyroidism, a common endocrine disorder characterized by insufficient thyroid hormone production, has been implicated in various metabolic disturbances, particularly weight gain and dyslipidemia, which could serve as pathways linking hypothyroidism to MASLD.[12], [13], [14] However, few studies have thoroughly investigated this potential mechanism.^15^ Thus, we used a nationwide matched case–control and MR approach to assess the association between hypothyroidism, hyperthyroidism, and MASLD, and to explore the potential mediating roles of metabolic traits in the hypothyroidism–MASLD association.

## Materials and methods

We conducted a nationwide matched case–control study to estimate the associations of a previous diagnosis of hypothyroidism and hyperthyroidism with biopsy-proven MASLD. To strengthen causality of the association, we performed a two-sample MR analysis using data from large-scale studies and biobanks. Mediation analysis was performed to understand the biological pathways.

### Nationwide matched case–control study

#### Data source and study population

The case–control analysis was based on data from the Epidemiology Strengthened by histoPathology Reports in Sweden (ESPRESSO) cohort, which prospectively collected liver histopathology data from all 28 pathology departments across Sweden between 1969 and 2017.^16^ Each histopathology report includes a unique personal identity number, biopsy date, liver topography, and morphological details. We identified all adult patients aged 18 years or older who underwent liver biopsy between 1969 and 2017. Among these, we first removed those who had emigrated from Sweden before the liver biopsy date (the index date) and then excluded patients with a history of alcohol abuse/misuse, other causes of acute or chronic liver disease, or previous liver transplantation, using a validated ICD algorithm (Table S1).^17^ We then defined patients with MASLD according to Systematized Nomenclature of Medicine Clinical Terms (SNOMED) and ICD codes (Table S2). This approach previously demonstrated a positive predictive value (PPV) of 92% for identifying MASLD.^17^ Patients with MASLD were further classified into four histological subgroups based on SNOMED definitions^18^: simple steatosis; non-alcoholic steatohepatitis (MASH) without fibrosis; MASLD with non-cirrhotic fibrosis; and cirrhosis. Individuals with liver biopsy data were matched with up to five reference individuals from the general population who had no record of earlier liver biopsy at time of matching, based on age, sex, county of residence, and the calendar year of biopsy. We also obtained data on individuals with liver biopsy data and their sibling controls without liver biopsy.

We further excluded patients with thyroid cancer and hyperthyroidism before MASLD diagnosis in the analysis of hypothyroidism, and patients with thyroid cancer and hypothyroidism before MASLD diagnosis in the analysis of hyperthyroidism (Fig. S1). Identical exclusion criteria were applied among the general population controls and sibling controls. We linked ESPRESSO to validated nationwide registers that contain data on demographics, comorbidities, prescribed medication, and mortality.

#### Ethical permit

The study was approved by the Stockholm Ethics Review Board (2014/1287-31/4, 2018/972-32, and 2022-05774-02), and informed consent was waived because of the register-based nature of the research.^19^

#### Definition of hypothyroidism and hyperthyroidism

Hypothyroidism was identified using ICD codes (ICD-8: 245,02, 245,03; ICD-9: 245C; ICD-10: E06.3) from the National Patient Register, which has recorded hospitalization data since 1964 and specialized outpatient visit data since 2001. Given that patients with hypothyroidism can be diagnosed and treated in primary care, we also used the prescription of levothyroxine (ATC H03AA01) and liothyronine (ATC H03AA02) as one of the definitions of hypothyroidism based on data from 1 July 2005 onward from the Swedish Prescribed Drug Register.^20^ Likewise, hyperthyroidism was defined using ICD codes (ICD-8: 242,0–2; ICD-9: 242A–E, 376D; ICD-10: E05.0–E05.3, H06.2) in the National Patient Register.

#### Assessment of covariates and potential mediators

We gathered comprehensive information on demographics, comorbidities, and medication use of each individual (Table S3). In brief, age at the index date (biopsy date for patients with MASLD or the corresponding matching date for controls), sex, date of birth, and emigration status were obtained from the Total Population Register.^21^ Education level was sourced from the Longitudinal Integrated Database for Health Insurance and Labour Market Studies (LISA),^22^ which provides education data for >98% of all individuals aged 25–64 years, with an estimated accuracy for highest attained level of education of 85%.^22^ Comorbidities, including pernicious anemia, metabolic disorders (diabetes, obesity, dyslipidemia, or hypertension), chronic obstructive pulmonary disease (COPD), and autoimmune diseases^23^ (Table S4) were identified using established ICD algorithms from the National Patient Register with PPVs ranging from 85% to 95%.^24^ COPD with diagnostic age ≥40 years was used as a proxy for heavy smoking. Medication data, including lipid-lowering drugs, antidiabetic medication, and antihypertensive medication, were retrieved from the Prescribed Drug Register.^20^ Given the temporal sequence of the diagnosis of metabolic disorder, hypothyroidism, and MASLD among cases, metabolic disorders diagnosed before hypothyroidism were deemed a confounder, whereas metabolic disorders diagnosed after hypothyroidism and before MASLD were treated as potential mediators (Fig. S2).

#### Statistical analyses

We conducted conditional logistic regression to assess the relationship between hypothyroidism, hyperthyroidism, and MASLD. Crude and multivariable-adjusted odds ratios (ORs) with 95% CIs were estimated, accounting for matching factors, including age, sex, calendar year, and county of residence. I addition, adjustments were made for potential confounders, including education level (≤9 years, 10–12 years, ≥13 years, or missing), country of birth (Nordic or other), metabolic disorders diagnosed before hypothyroidism, COPD, pernicious anemia, autoimmune diseases, and the number of inpatient and outpatient visits over the past 12 months before the index/matching date (used as a proxy for healthcare utilization). Assuming the causality between hypothyroidism and MASLD, we used the ‘mediation’ R package^25^ (R Foundation for Statistical Analysis, Vienna, Austria) to estimate the mediation of metabolic disorders diagnosed after hypothyroidism. The analysis is based on nonparametric bootstrapping to generate mediation effects, allowing for robust inference about the mediation pathways. We then performed the analysis among patients with MASLD by treating simple steatosis as the reference group. We conducted a sensitivity analysis by changing the control population to full siblings of patients with MASLD to control for potential confounding factors related to genetic predisposition and shared childhood exposures. Subgroup analyses were performed stratified by sex (men and women) and age (<40 years and ≥40 years). For hyperthyroidism, we abstained from subgroup and mediation analyses because of inadequate statistical power. All analyses were performed using R version 4.4.1 (R Foundation for Statistical Computing, Vienna, Austria), with statistical significance defined as a two-sided *p* value of ≤0.05.

### MR study

#### Study design

Hypothyroidism is characterized by low levels of thyroid hormones and increased levels of thyroid-stimulating hormone (TSH). Thus, we used TSH and clinically diagnosed hypothyroidism and hyperthyroidism as the exposures in the MR analyses. We first conducted a two-sample MR analysis to investigate the associations of genetic liability to higher levels of TSH, hypothyroidism, and hyperthyroidism with risk of MASLD. We further conducted multivariable MR analyses to examine whether metabolic traits mediated the associations. Given that MR analyses were based on publicly available summary data, an ethical permit was not required.

#### Genetic instrument selection

The TSH genome-wide association study (GWAS) data werebsourced from the ThyroidOmics Consortium (https://transfer.sysepi.medizin.uni-greifswald.de/thyroidomics/datasets/), where TSH levels were measured in 271,040 European participants whose levels fell within the upper and lower 2.5% of the TSH distribution, followed by inverse normal transformation.^26^ The study accounted for assay and population characteristics, as well as environmental factors, such as population iodine supply.^26^ GWAS data on hypothyroidism were extracted from the Veteran Authority’s (VA) Million Veteran Program, where hypothyroidism was defined by Phecode 244 with diagnostic data from hospital electronic health records.^27^ To minimize population structure bias, we obtained data on participants of European ancestry, including 62,814 cases and 378,321 non-cases of MASLD (https://www.ncbi.nlm.nih.gov/projects/gap/cgi-bin/study.cgi?study_id=phs002453.v1.p1). Summary GWAS data on hyperthyroidism were available in a meta-analysis of three biobanks including 3,271 cases and 512,267 non-cases of MASLD.^28^ We first obtained all genetic variants (*i.e.* single nucleotide polymorphisms [SNPs]) associated with the exposure at the genome-wide significance level (*p* <5 × 10^-8^) and then clumped these SNPs by linkage disequilibrium (*r*^*2*^ <0.001). In total, 254, 265, and 24 SNPs were used as genetic instruments for TSH, hypothyroidism, and hyperthyroidism, respectively. All retained SNPs had F statistic >10, indicating limited weak instrument bias.^29^ Detailed information on used SNPs is available in Table S5.

#### Data sources for MASLD

Summary-level data on MASLD were obtained from a GWAS meta-analysis of UK Biobank, Estonian Biobank, eMERGE, and FinnGen, including 8,434 MASLD cases, all identified through electronic health records, and 770,180 non-cases of MASLD.^30^ Detailed diagnostic codes can be found in the original GWAS.^30^ Associations were adjusted for age, sex, and top genetic principal components.

#### Data sources for metabolic traits

Based on previous findings,^31^ obesity, type 2 diabetes mellitus (T2DM), triglycerides and systolic blood pressure were selected as MASLD-associated metabolic traits and included in the multivariable MR analysis. Summary-level data on BMI,^32^ T2DM (www.ncbi.nlm.nih.gov/projects/gap/cgi-bin/study.cgi?study_id=phs002453.v1.p1),^33^ triglycerides (www.nealelab.is/uk-biobank), and systolic blood pressure^34^ were available in GWASs including 806,834, 1,407,282 (228,499 cases), 343,992, and 1,006,863 individuals of European ancestry, respectively.

#### Statistical analyses

Exposure and outcome data were harmonized based on effect and non-effect alleles. SNPs with allele mismatch were removed. We used the inverse variance weighted (IVW) method with multiplicative random effects as the primary analysis, supplemented by three sensitivity analyses: the weighted median method,^35^ MR-Egger regression,^36^ and MR-PRESSO.^37^ The IVW method is known for its precision but is highly sensitive to SNP outliers, which can lead to horizontal pleiotropy. By contrast, the weighted median method can provide a robust estimate when more than half of the total weight comes from valid SNPs. The MR-Egger regression includes an intercept test to assess horizontal pleiotropy and offers corrected estimates, although it tends to be underpowered. It also detects outlier SNPs and recalculates estimates after their removal, improving the robustness of the results. We calculated Cochran’s Q to evaluate heterogeneity among SNP estimates. Horizontal pleiotropy was assessed using the MR-Egger intercept test and the MR-PRESSO global test. Multivariable MR using exposure-specific instruments was performed using the MendelianRandomization R package and mediation of metabolic traits was calculated using the formula: (total effect-direct effect)/total effect. Two-sample MR analysis was conducted using the TwoSampleMR package in R 4.3.2. Statistical significance was set at a two-sided *p* value of ≤0.05.

## Results

### Nationwide matched case–control study

The characteristics of the cases of MASLD and general population controls included in the study are shown in Table 1. In the analysis of hypothyroidism, we included a total of 12,172 MASLD cases with different histological features and 56,831 matched population comparators. Patients with MASLD were more frequently diagnosed with diabetes mellitus, obesity, dyslipidemia, and hypertension compared with controls. For the analysis treating siblings as controls, we included 5,478 MASLD cases with 10,682 sibling comparators.

**Table 1:** Characteristics of MASLD cases and controls.

Characteristic	Population controls	All MASLD	Simple steatosis	MASH without fibrosis	Non-cirrhotic fibrosis	Cirrhosis
	n = 56,831	n = 12,172	n = 8,144	n = 1,399	n = 1,941	n = 688
Sex, n (%)						
Men	30,795 (54.2)	6,666 (54.8)	4,517 (55.5)	721 (51.5)	1,052 (54.2)	376 (54.7)
Women	26,036 (45.8)	5,506 (45.2)	3,627 (44.5)	678 (48.5)	889 (45.8)	312 (45.3)
Age						
Mean (SD)	52.1 (16.4)	52.4 (16.4)	51.9 (16.0)	52.1 (17.1)	52.4 (17.9)	58.5 (13.7)
Median (IQR)	54 (41–64)	54 (42–64)	53 (41–64)	54 (41–64)	56 (43–65)	61 (52–67)
Country of birth, n (%)						
Nordic country	52,097 (91.7)	10,946 (89.9)	7,376 (90.6)	1,241 (88.7)	1,705 (87.8)	624 (90.7)
Other	4,734 (8.3)	1,226 (10.1)	768 (9.4)	158 (11.3)	236 (12.2)	64 (9.3)
Education level∗, n (%)						
≤9 years	18,027 (31.7)	3,959 (32.5)	2,643 (32.5)	443 (31.7)	606 (31.2)	267 (38.8)
10–12 years	22,016 (38.7)	4,963 (40.8)	3,266 (40.1)	596 (42.6)	829 (42.7)	272 (39.5)
≥13 years	14,234 (25.1)	2,466 (20.3)	1,681 (20.6)	278 (19.9)	417 (21.5)	90 (13.1)
Missing	2,554 (4.5)	784 (6.4)	554 (6.8)	82 (5.8)	89 (4.6)	59 (8.6)
Start of follow-up, year						
1969–1989	11,016 (19.4)	2,293 (18.9)	1,815 (26.2)	182 (13.0)	159 (8.2)	137 (20.0)
1990–2005	31,694 (55.8)	6,751 (55.5)	4,723 (58.1)	787 (56.3)	893 (46.0)	348 (50.7)
2006–2019	14,063 (24.8)	3,116 (25.6)	1,598 (19.6)	429 (30.7)	888 (45.8)	201 (29.3)

**Comorbidities, n (%)**						
Any metabolic comorbidity	7,092 (12.5)	2,975 (24.4)	1,654 (20.3)	379 (27.1)	704 (36.3)	238 (34.6)
Diabetes	1,798 (3.2)	1,137 (9.3)	585 (7.2)	134 (9.6)	271 (14.0)	147 (21.4)
Obesity	111 (0.2)	218 (1.8)	121 (1.5)	39 (2.8)	51 (2.6)	7 (1.0)
Dyslipidemia	3,994 (7.0)	1,402 (11.5)	786 (9.7)	191 (13.7)	336 (17.3)	89 (12.9)
Hypertension	3,361 (5.9)	1,306 (10.7)	659 (8.1)	175 (12.5)	349 (18.0)	123 (17.9)
No. of hospital visits over past 12 months (mean, SD)	2.6 (8.1)	8.6 (17.8)	6.9 (14.5)	9.8 (18.9)	14.6 (26.0)	8.9 (17.8)

All variables reported as mean (SD) or n (%), unless described otherwise.

MASH, metabolic dysfunction-associated steatohepatitis.

∗ Education categories were based on compulsory school, high school, and college. Education level was recorded beginning in 1990; thus, data presented are for persons with index dates on or after 1 January, 1990. Persons with index dates before 1990 had their education level recorded as missing.

A previous diagnosis of hypothyroidism was observed in 309 (2.5%) patients with MASLD and 819 (1.4%) controls, indicating a 1.81-fold increased odds of MASLD (95% CI 1.57–2.09) in the crude model. This association persisted in the multivariable analysis (OR 1.68; 95% CI 1.36–2.06). In addition, the relationship between previous hypothyroidism and the development of MASLD varied across histological stages. The OR increased from 1.22 (95% CI 0.91–1.65) for simple steatosis to 2.54 (95% CI 1.44–4.48) for MASH without fibrosis, and further to 2.58 (95% CI 1.76–3.78) for MASLD with non-cirrhotic fibrosis (Table 2). However, for cirrhosis, the OR was 1.46 (95% CI 0.54–3.96). This pattern was also observed in the analysis among MASLD by treating simple steatosis as the reference group (Table S6).

**Table 2:** Association between hypothyroidism and MASLD among MASLD cases and general population controls.

	Population controls	All MASLD	Simple steatosis	MASH without fibrosis	Non-cirrhotic fibrosis	Cirrhosis
	n = 56,831	n = 12,172	n = 8,144	n = 1,399	n = 1,941	n = 688
%	1.4	2.5	1.6	3.7	5.3	2.8
Crude model, OR (95% CI)	1 (ref.)	1.81 (1.57–2.09)	1.55 (1.26–1.92)	2.11 (1.47–3.04)	2.32 (1.79–3.00)	1.29 (0.75–2.21)
Multivariable-adjusted model, OR (95% CI)	1 (ref.)	1.68 (1.36–2.06)	1.22 (0.91–1.65)	2.54 (1.44–4.48)	2.58 (1.76–3.78)	1.46 (0.54–3.96)

Estimates were obtained from conditional logistic regression; all models were conditioned on stratum factors (age, sex, biopsy calendar year, and county). Multivariable-adjusted model was adjusted for education, country at birth, metabolic disorders (obesity, type 2 diabetes mellitus, dyslipidemia, and hypertension), autoimmune disease, chronic obstructive pulmonary disease, anemia, and clinical visit in the past 12 months.

MASH, metabolic dysfunction-associated steatohepatitis; OR, odds ratio.

Subgroup analysis revealed possibly higher risks of MASLD following a previous hypothyroidism diagnosis in men and in the group younger than 40 years (Table S7). The OR of MASLD was 3.10 (95% CI 2.02–4.76) in men and 5.32 (95% CI 2.29–12.34) in the group younger than 40 years. The association between hypothyroidism and MASLD remained stable in the sensitivity analysis treating unaffected siblings as controls (Table S8). The mediation analysis revealed that 41.2% (95% 30.1–53.0%) of the hypothyroidism–MASLD association was mediated by metabolic disorders diagnosed after hypothyroidism and before MASLD.

In the analysis of hyperthyroidism, we included a total of 11,875 MASLD cases and 56,248 matched population comparators. A previous diagnosis of hyperthyroidism was observed in 11 (0.09%) patients with MASLD and 69 (0.13%) controls. Compared with non-hyperthyroidism, prior diagnosis of hyperthyroidism was associated with a reduced odds of MASLD in the crude model (OR 0.74, 95% CI 0.39–1.40) and (OR 0.17, 95% CI 0.05–0.56) in the multivariable analysis. The association became clear after adjusting for autoimmune disease and remained stable in the analysis treating unaffected siblings as controls.

### MR study

Out of the 254 SNPs associated with TSH, 226 were available in the MASLD GWAS dataset, whereas 219 of the 265 SNPs associated with hypothyroidism were present in the same dataset. Genetically predicted higher levels of TSH and genetic liability to hypothyroidism were associated with an increased risk of MASLD (Table 3). The OR of MASLD was 1.10 (95% CI 1.03–1.17) per SD increase in genetically predicted inverse normal transformed TSH levels, and 1.07 (95% CI 1.03–1.12) per unit increase in genetic predisposition to hypothyroidism. Genetic liability to hyperthyroidism was not associated with MASLD (OR 0.92, 95% CI 0.84–1.01; *p* = 0.096). The associations remained generally consistent in sensitivity analyses and no significant heterogeneity or horizontal pleiotropy was detected (Table 3).

**Table 3:** Genetically predicted higher levels of TSH and hypothyroidism in relation to risk of MASLD in MR analysis.

Exposure	Method	OR (95% CI)	*p* value
Genetically predicted TSH levels	Inverse variance weighted with multiplicative random effects	1.10 (1.03–1.17)	0.004
	Weighted median	1.11 (1.00–1.24)	0.049
	MR-Egger	1.02 (0.91–1.15)	0.735
	Cochran’s Q = 224; *P*_MR-Egger intercept test_ = 0.159; *P*_MR-PRESSO global test_ = 0.410		
Genetic liability to hypothyroidism	Inverse variance weighted with multiplicative random effects	1.07 (1.03– 1.12)	0.001
	Weighted median	1.07 (1.00–1.15)	0.045
	MR-Egger	1.05 (0.96–1.15)	0.282
	Cochran’s Q = 206; *P*_MR-Egger intercept test_ = 0.513; *P*_MR-PRESSO global test_ = 0.617		
Genetic liability to hyperthyroidism	Inverse variance weighted with multiplicative random effects	0.92 (0.84–1.01)	0.096
	Weighted median	0.94 (0.79–1.12)	0.499
	MR-Egger	0.91 (0.67–1.25)	0.567
	Cochran’s Q = 14; *P*_MR-Egger intercept test_ = 0.931; *P*_MR-PRESSO global test_ = NA		

Results were obtained using method listed in the ‘Method’ column and are given as OR with 95% CI for genetic liability to MASLD as outcome; *p* <0.05 were considered statistically significant. MR-PRESSO detected no outlier in either analysis. MASH, metabolic dysfunction-associated steatohepatitis; MR, Mendelian randomization; OR, odds ratio; TSH, thyroid-stimulating hormone.

Approximately 5.4% and 19.4% of the associations between genetically predicted TSH and MASLD was mediated by BMI and triglycerides, respectively. Likewise, 2.8% and 9.7% of the associations between genetically predicted hypothyroidism and MASLD was mediated by BMI and triglycerides, respectively. However, these analyses were underpowered to detect statistically significant mediation effects (Table S9). Limited mediation was observed for genetically predicted T2DM or systolic blood pressure.

## Discussion

This nationwide case–control and MR study identified a positive association between previous hypothyroidism and an elevated risk of developing MASLD. This association was pronounced in men and individuals younger than 40 years. The association intensified in more advanced stages of MASLD, peaking with non-cirrhotic fibrosis, but did not continue to increase as the disease progressed to cirrhosis. Metabolic disorders that developed after diagnosis of hypothyroidism mediated ∼41% of this association. We also found an inverse association between a previous diagnosis of hyperthyroidism and MASLD development; however, this association was not observed in the MR analysis. In absolute terms, hypothyroidism was uncommon and seen in 2.5% in people living with MASLD and in 1.4% controls. Although hypothyroidism might have a lower prevalence, the condition is common enough to have a meaningful population health impact.

Our finding of a positive association between hypothyroidism and MASLD is consistent with previous research.^5^^,^^6^ A meta-analysis of 13 studies (two cohorts, four case–control, and seven cross-sectional with a sample size up to 18,544) reported a significant association between hypothyroidism and MASLD (OR, 1.52, 95% CI 1.24–1.87), despite considerable heterogeneity among the studies.^5^ Another meta-analysis, which included 12 cross-sectional and three longitudinal studies, also found a similar association (OR, 1.42, 95% CI 1.15–1.77).^6^ A positive association was also observed between higher levels of TSH and higher levels of liver fibrosis.^38^ Furthermore, an MR study utilizing GWAS data from 1,483 European non-alcoholic fatty liver disease cases and 17,781 controls provided additional evidence supporting this link.^39^ Our study, which leveraged a nationwide dataset and was well powered, further reinforces the positive association between hypothyroidism and MASLD, adding robust support to the existing body of evidence.

The meta-analysis of three studies, although limited by small sample sizes, investigated the link between hypothyroidism and the risk of severe MASLD,^6^ finding that the magnitude of risk closely mirrored the severity of MASLD histology.^6^ This partially aligns with our results in that we observed a similar trend, but did not observe a continued increase in risk for cirrhosis. Interestingly, another study reported that, although hypothyroidism was highly prevalent among patients with MASLD and was associated with increased MASLD inflammatory activity, it was not linked to fibrosis or grade of steatosis.^40^ A potential explanation for this is that, given that the development of cirrhosis is a prolonged process, hypothyroidism diagnosed in the 5–10 years before cirrhosis diagnosis might have only a marginal impact on liver disease progression. In addition, this might reflect that hypothyroidism might not be a universal driver of MASLD, but rather a factor in a specific subgroup of patients who have a unique hepatic or systemic metabolic response. This hypothesis is partly supported by the results of the trial of resmetirom, where only ∼30% of the high-dose patients responded.^3^ However, we cannot rule out that the null association for grade of steatosis might be caused by underdiagnosis of subclinical hypothyroidism or inadequate power because of the inclusion of fewer patients with cirrhosis. Nonetheless, these findings collectively underscore the potential importance of early detection and management of hypothyroidism in mitigating the progression of MASLD.

In our study, we observed a notably stronger association between hypothyroidism and MASLD in men (OR, 3.10), which aligns with findings from a Korean cross-sectional study.^7^ In addition, we identified a more pronounced association in individuals younger than 40 years (OR, 5.32) compared with those older than 40 years (OR, 1.49). These findings suggest that younger individuals and men are at higher risk for MASLD in the context of hypothyroidism. MASLD is most commonly the consequence of long-standing obesity. Thus, these data underscore the fact that an alternative underlying mechanism leading to the development of MASLD in younger individuals might be hypothyroidism. However, future studies examining all patients in such subgroups are needed.

Although the role of metabolic disorders as intermediaries between hypothyroidism and MASLD has been suggested,[13], [14], [15] empirical evidence remains limited. Our cohort study found that ∼41% of the association between hypothyroidism and MASLD might be mediated by metabolic disorders. Notably, multivariable MR analysis identified triglycerides as a key mediator, consistent with findings from animal studies.^12^ These are consistent with the understanding that metabolic disorder is a key metabolic risk factor for MASLD. Although a larger proportion of the hypothyroidism–MASLD association was mediated by metabolic disorders, hypothyroidism still retained a direct, unmediated effect on MASLD risk. These findings emphasize the dual role of hypothyroidism as: (1) an upstream factor that contributes to metabolic disorders; and (2) an independent risk factor for MASLD through mechanisms unrelated to traditional metabolic disorders, such as direct effects on liver metabolism, inflammation, or fibrosis pathways, implying that liver cells metabolize intracellular fat without noticeable changes in systemic metabolism. Thus, further research is necessary to investigate other alternative pathways and enhance our understanding of the relationship between hypothyroidism and MASLD.

We also found a lower risk of MASLD in patients with hyperthyroidism. This is consistent with earlier studies^9^^,^^10^^,^^41^ and in line with recent trial data demonstrating a beneficial effect of resmetirom.^3^

Our paper has some strengths. As a nationwide case–control study, >12,000 individuals with biopsy-verified MASLD were included. Excluding other liver diseases, our definition of MASLD had a high PPV.^17^ We adjusted our analyses for a large number of covariates, including healthcare consumption, and confirmed our hypothyroidism results in a sibling comparison that should have also minimized intrafamilial confounding. Not all patients with hyperthyroidism and hypothyroidism are detected through the National Patient Registry and, therefore, we also exploited Prescribed Drug Register data to increase our sensitivity for thyroid disease. Finally, we carried out MR analyses to triangulate our findings. Notably, directly comparing the magnitude of observational and MR associations was challenging because of differences in exposure measurement, that is, clinical diagnosis in the case–control study versus genetic predisposition in MR. However, these two approaches complement each other: the case–control study reflects real-world clinical implications by using actual disease diagnoses, whereas MR strengthens causal inference by mitigating confounding and reverse causation through the use of genetic instruments.

We acknowledge several limitations in our study. First, the analysis was limited to individuals who underwent liver biopsy, which inherently introduces selection bias. Liver biopsy is typically performed in patients with more severe or atypical presentations of disease, making this cohort less representative of the broader MASLD population. This selection bias is evidenced by the lower rate of simple steatosis observed in our study (67%) compared with the expected rate of 75–80% in the general population. However, the persistence of associations between thyroid diseases and MASLD in this cohort suggests that these links are not confined to early stages of the disease, but also extend to more advanced stages. This highlights the potential role of thyroid diseases as clinically significant risk factors across the MASLD spectrum. By contrast, the observed discrepancy might also partly reflect potential misclassification or variability in pathologist coding practices, particularly given the absence of centralized biopsy reading. Nevertheless, the previous validation of pathology reports provides confidence that steatosis was accurately identified, mitigating concerns about misclassification.^17^ Second, we lacked detailed data on the specific indications for liver biopsy. Although liver biopsy in Sweden is generally performed in patients with clinical suspicion of advanced fibrosis or alternative diagnoses, the absence of precise indication data limited our ability to fully contextualize the findings. Third, the general population comparator group did not undergo liver biopsy, preventing direct histological comparisons. This approach assumed that these individuals did not have clinically significant liver disease; however, the possibility of undetected MASLD or other liver conditions in this group cannot be entirely excluded, which could introduce potential bias. Nonetheless, given the invasive nature and associated risks of liver biopsy, it would be both clinically and scientifically inappropriate to compare patients with MASLD to individuals with mild findings who underwent biopsy that turned out to be ‘normal’. In addition, any misclassification of MASLD within the comparator group would likely bias the results toward the null, thereby underestimating the true association. This conservative bias strengthens confidence in the observed findings. Fourth, in the ESPRESSO cohort, we lacked data on thyroid-related hormones, liver function tests, or related biomarkers, which limits our ability to explore mechanistic pathways underlying the observed associations. Fifth, the analyses of hyperthyroidism and the subgroup analysis for cirrhosis were limited by low statistical power, potentially affecting the robustness of these findings. Sixth, while we identified associations between hypothyroidism, hyperthyroidism, and MASLD, we did not have data on the impact of hypothyroidism treatments on liver disease severity. Future studies incorporating treatment data and additional biomarkers could provide more comprehensive insights into these relationships. Finally, the absence of GWAS data on MASLD histopathological subtypes limited our ability to explore the associations between genetically predicted thyroid-related indicators and MASLD severity using MR.

In summary, our study found a significant association between hypothyroidism and an increased risk of developing MASLD, with a particularly pronounced risk in younger individuals and men. This risk escalated with MASLD severity, peaking at non-cirrhotic fibrosis, underscoring the importance of early intervention to mitigate disease progression. Finally, we found a potential inverse relationship between hyperthyroidism and MASLD, supporting an effect of newly established thyroid hormone receptor β-agonist treatment in MASLD.

## Abbreviations

COPD, chronic obstructive pulmonary disease; ESPRESSO, Epidemiology Strengthened by histoPathology Reports in Sweden cohort; GWAS, genome-wide association study; ICD, International Classification of Diseases; IVW, inverse variance weighted; LISA, Longitudinal Integrated Database for Health Insurance and Labour Market Studies; MASH, non-alcoholic steatohepatitis; MASLD, metabolic-associated steatotic liver disease; MR, Mendelian randomization; OR, odds ratio; PPV, positive predictive value; SNOMED, Systematized Nomenclature of Medicine Clinical Terms; SNPs, single nucleotide polymorphisms; T2DM, type 2 diabetes mellitus; TSH, thyroid-stimulating hormone; VA, Veteran Authority.

## Financial support

SY is supported by an American Heart Association Postdoctoral Fellowship (24POST1189614).

## Authors’ contributions

Conceived and designed the study; authored the initial draft of the manuscript: SY, JFL.

Contributed to data collection: SY, XR, JFL.

Undertook the statistical analyses: SY.

Contributed to data interpretation, offered significant intellectual insights to the manuscript, and approved its final version: SY, FE, DB, MV, ES, XR, JC, HH, JFL.

## Data availability statement

Data are not available because of Swedish data protection regulations.

## Conflicts of interest

FE has served as an advisory board member for Boehringer Ingelheim. HH’s institutions have received research funding from Astra Zeneca, EchoSens, Gilead, Intercept, MSD, Novo Nordisk, and Pfizer. HH has served as consultant or on advisory boards for Astra Zeneca, Bristol Myers-Squibb, MSD, and Novo Nordisk, and has been part of hepatic events adjudication committees for Arrowhead, Boehringer Ingelheim, KOWA and GW Pharma. JFL has coordinated an unrelated study on behalf of the Swedish Inflammatory Bowel Disease (IBD) quality register (SWIBREG). This study received funding from Janssen. He has also received financial support from MSD to develop a paper reviewing national healthcare registers in China and has funding from Takeda for a celiac disease project. JFL also has ongoing collaborations with MSD focusing on IBD. The other authors have no conflicts of interest to disclose.

Please refer to the accompanying ICMJE disclosure forms for further details.
