# Supplementary material for: Thyroid dysfunction in MASLD: Results of a nationwide study
Source: JHEP Rep. 2025 Feb 26;7(5):101369. doi: 10.1016/j.jhepr.2025.101369 (PMC12060450; doi:10.1016/j.jhepr.2025.101369)
Supplement: Multimedia component 1 [file mmc1.pdf]

Thyroid dysfunction in MASLD: Results of a nationwide study

Shuai Yuan, Fahim Ebrahimi, David Bergman, Marijana Vujković, Eleonora Scorletti, Xixin Ruan, Jie Chen, Hannes Hagström, Jonas F. Ludvigsson

Table of contents

Table S1 2
Table S2 3
Table S3 4
Table S4 5
Table S5 7
Table S6 20
Table S7 21
Table S8 22
Table S9 23
Fig. S1. 24
Fig. S2. 25

Table S1. Study exclusion criteria

Excluded Conditions[1]	ICD-7 / ICD-8	ICD-9*	ICD-10
Alcohol abuse/misuse or Alcohol-related liver disease	280,00; 281,00; 307,00; 307,10; 307,99; 322; 581,10; 583,10; 261,00; 262,00; 291; 291,1; 303; 571,00; 571,01; 979; 980,00; 980,01; 980,98; 980,99	291; 294A; 303; 305A; 357F; 425F; 535D; 571A-D; 760W; 790D; 977D; 980A; 980X; V97B	E24.4; F10; G31.2; G62.1; G72.1; I42.6; K29.2; K70; K86.0; Q35.4; R78.0; T51.0; T51.8; T51.9; X65; Y15; Y57.3; Y90; Y91; Z50.2; Z71.4; Z71.2
Other abuse- and drug-related diagnoses	571,0; E860; N980	571A-D	F11-F19
Drug-induced liver disease	–	573D	K71
Viral hepatitis (e.g., hepatitis B, C)	070; 999,20	070	B15-19; B00.8; B25.1
Budd-Chiari	–	453A	I82
Liver abscess	572	572A	K75.0; A06.4
HIV	079,83; Y40,49; Y41,49	279K	B20-B24
Haemochromatosis	273,2	275A	E83.1
Wilson’s disease	273,3	275B	E83.0
Autoimmune hepatitis	–	573D; 571E	K75.4
Primary biliary cholangitis	–	571G	K74.3; K74.4
Other cholangitis	574,06	576B	K83; K83.0A
Alpha-1 antitrypsin deficiency	–	277G	E88.0
Glycogen storage disease	–	271	E74
Liver transplantation†	–	V42H	Z94.4<br>JJC, DJ005; DJ006†
Gastric bypass surgery	–	–	JDF†

†Liver transplantation and Bariatric surgery were further defined via procedure codes.
[1] We excluded any person with a diagnosis of another aetiology of liver disease, or alcohol abuse/misuse or alcohol-related liver disease, defined on or before the index date.
*Note that in the Swedish versions of ICD-9, the fourth position of the code is denoted by a letter, and not by a digit. This letter corresponds alphabetically to the fourth digit that is used in US versions of ICD (for example, 571A corresponds to 571.0).
Abbreviations: HIV, Human Immunodeficiency Virus; ICD, International Classification of Disease.

Table S2: Definition of MASLD subgroups according to histology

**Subgroup**	**SNOMED and ICD codes**	
	**Inclusion**	**Exclusion**
Cirrhosis	M4950x	
MASLD with non-cirrhotic fibrosis<br>(note that this may or may not include NASH)	Steatosis: M5008x or M5520x, PLUS at least one fibrosis code: M49 [exactly], M4900x or M49060.	Cirrhosis: M495 [exactly] or M4950x.
NASH without fibrosis	Steatosis: either M5008x or M5520x, PLUS at least one of the following:<br>1. any M4- code, or<br>2. M5400x	M4 defines a broad category of inflammation, both acute and chronic.<br>Cannot have any of the following:<br>Fibrosis codes (M49 [exactly], M4900x or M49060) OR<br>Cirrhosis: M495 [exactly] or M4950x.
Simple steatosis	M5008x or M5520x	Cannot have any of the following:<br>Inflammation codes: M4- or M5400x OR<br>Fibrosis: M49 [exactly], M4900x or M49060 OR<br>Cirrhosis: M495 [exactly] or M4950x

Abbreviations: ICD, International Classification of Diseases; NASH, Nonalcoholic steatohepatitis; SNOMED, Systematized Nomenclature of Medicine.

Table S3. Definitions of covariates

Covariate	ICD8 (1969-1986)	ICD9 (1987-1996)	ICD10 (1997-)	ATC code
Pernicious anemia	281	281A	D51	
Diabetes mellitus	250<br><br>Or any of the ATC medication codes (see right-most column)	250<br><br>Or any of the ATC medication codes (see right-most column)	E10-E14<br>(Note: E14 also includes E14.0-E14.9)<br>Or any of the ATC medication codes (see right-most column)	A10A<br>A10AB-AE<br>A10BA-BX
Obesity	278<br>649,1	278<br>649B	E650-E679<br>E65<br>E66	
Dyslipidemia	272;<br>Or any of the ATC medication codes	272<br>Or any of the ATC medication codes	E78<br>Or any of the ATC medication codes	C10AA<br>C10BA<br>C10BX<br>C10AB<br>C10AC
Hypertension	400-404	401-405	I10-I13, I15	C09
COPD (≥40 years)		491, 492, 496	J41, 42,43,44	

COPD. Chronic obstructive Pulmonary Disease.

Table S4. Definitions of autoimmune diseases*

Autoimmune disease	ICD code
Systemic/cutaneous lupus erythematosus	– ICD-8: 734,1<br>– ICD-9: 710A<br>– ICD-10: M32, L931
Vitiligo	– ICD-8: 709,05<br>– ICD-9: -<br>– ICD-10: L80
Psoriasis	– ICD-8: 696,0 and 696,1<br>– ICD-9: 696B<br>– ICD-10: L40
Alopecia areata	– ICD-8: 704,00<br>– ICD-9: -<br>– ICD-10: L63
Systemic sclerosis	– ICD-8: 734,00-734,19<br>– ICD-9: 710B<br>– ICD-10: M34
Dermatomyositis or juvenile dermatomyositis	– ICD-8: 716,00<br>– ICD-9: 710D<br>– ICD-10: M33 except M33.2
Polymyositis	– ICD-8: 716,10<br>– ICD-9: 710E<br>– ICD-10: M33.2
Type 1 diabetes	– ICD-8: 250 (limited to first diagnosis ≤30 years)<br>– ICD-9: 250 (limited to first diagnosis ≤30 years)<br>– ICD-10: E10
Addison´s disease	– ICD-8: 255,10<br>– ICD-9: 255E<br>– ICD-10: E27.1 and E27.2
Autoimmune hepatitis	– ICD-8: 573,0 and 571,9<br>– ICD-9: 573D, 573E<br>– ICD-10: K75.4
Primary biliary cholangitis	– ICD-8: -<br>– ICD-9: 571G<br>– ICD-10: K74.3
Inflammatory bowel disease	– ICD8: 569,04; 563,1; 563,10; 569,02; 563,00<br>– ICD-9: 555, 556<br>– ICD-10: K50, K51 and K52.3
Sjögren syndrome	– ICD-8: 734,90

	– ICD-9: 710C<br>– ICD-10: M35.0
Myasthenia gravis	– ICD-8: 733,00<br>– ICD-9: 358A<br>– ICD-10: G70.0
Rheumatoid arthritis	– ICD-8: 712,10; 712,20; 712,38; 712,39<br>– ICD-9: 714A; 714B; 714C; 714W; 719D<br>– ICD-10: M05; M06.0; M06.2; M06.3, M06.8; M06.9; M12.3
Multiple sclerosis	– ICD-8: 340,99<br>– ICD-9: 340<br>– ICD-10: G35
Ankylosing spondylitis	– ICD-8: 712,40<br>– ICD-9: 720A<br>– ICD-10: M45
Spondyloarthritis	– ICD8: 720,1 and 720,2<br>– ICD-9: 720A; 696A; 713B; 099D; 711A; 720B; 720C; 720W<br>– ICD-10: M45; M08.1; L40.5; M07.0-3; M07.4-5; M07.6; M09.1.2; M02.0-2; M02.3; M02.8-9; M46.0/M46.1/8/9
Sarcoidosis	– ICD-8: 135<br>– ICD-9: 135<br>– ICD-10: D86; G53.2; M63.3
Celiac disease	– ICD-8: 269,00<br>– ICD-9: 579A<br>– ICD-10: K90.0

* Code reference: Yuan S, Leffler D, Lebwohl B, Green PHR, Larsson SC, Söderling J, Sun J, Ludvigsson JF. Older age of celiac disease diagnosis and risk of autoimmune disease: A nationwide matched case-control study. J Autoimmun. 2024;143:103170.

Table S5. Genetic instruments for TSH, hypothyroidism, and hyperthyroidism

Exposure	SNP	EA	EAF	Beta	SE	P value	F statistic
TSH	rs4268443	A	0.31	-0.017	0.003	4.26E-08	30
TSH	rs10748781	A	0.57	-0.059	0.003	1.97E-87	387
TSH	rs11190164	A	0.74	-0.020	0.003	1.69E-09	37
TSH	rs1931666	A	0.69	-0.018	0.003	3.52E-08	30
TSH	rs12783458	C	0.63	0.023	0.003	5.57E-14	57
TSH	rs17481499	T	0.17	0.046	0.004	9.13E-33	146
TSH	rs11255770	T	0.24	-0.024	0.003	1.16E-12	50
TSH	rs4933466	A	0.60	0.033	0.003	2.67E-28	121
TSH	rs4934383	T	0.66	-0.023	0.003	8.64E-14	54
TSH	rs12411841	T	0.97	-0.055	0.008	1.32E-11	46
TSH	rs74451129	T	0.28	-0.024	0.004	1.77E-11	45
TSH	rs7417283	T	0.80	0.033	0.004	1.78E-19	80
TSH	rs17020127	A	0.91	-0.103	0.005	1.57E-88	405
TSH	rs11605461	A	0.48	-0.039	0.003	6.91E-41	179
TSH	rs6590334	T	0.48	0.017	0.003	5.69E-09	34
TSH	rs11221835	T	0.23	-0.022	0.003	1.33E-10	42
TSH	rs11602347	C	0.60	-0.024	0.003	9.20E-15	59
TSH	rs179785	A	0.48	0.018	0.003	4.35E-10	39
TSH	rs11038357	A	0.28	-0.065	0.003	1.26E-92	415
TSH	rs11204756	A	0.50	-0.019	0.003	3.91E-11	44
TSH	rs2581928	A	0.27	-0.022	0.003	8.69E-12	45
TSH	rs6669935	C	0.43	0.018	0.003	1.91E-09	37
TSH	rs2229738	T	0.09	-0.039	0.006	8.41E-13	51
TSH	rs523587	T	0.43	0.016	0.003	2.29E-08	32
TSH	rs56279106	A	0.27	-0.020	0.003	2.03E-09	37
TSH	rs10751136	A	0.50	0.019	0.003	3.89E-11	43
TSH	rs1138328	C	0.72	0.019	0.003	1.57E-08	33
TSH	rs2147948	A	0.18	-0.028	0.004	7.99E-13	51
TSH	rs7529294	T	0.54	-0.018	0.003	1.16E-09	37
TSH	rs139260332	A	0.01	-0.100	0.015	7.37E-12	47
TSH	rs12091047	T	0.35	0.067	0.003	8.98E-106	466
TSH	rs10799824	A	0.16	-0.119	0.004	5.12E-194	881
TSH	rs12133889	A	0.39	0.029	0.003	2.21E-22	93
TSH	rs4844563	A	0.67	-0.017	0.003	3.54E-08	30
TSH	rs1920413	A	0.46	0.016	0.003	4.84E-08	29
TSH	rs3184504	T	0.49	0.030	0.003	2.47E-25	108
TSH	rs10743983	A	0.55	-0.018	0.003	7.23E-10	39
TSH	rs1690789	T	0.48	-0.029	0.003	5.11E-23	99
TSH	rs2807367	C	0.67	0.020	0.003	6.07E-11	42
TSH	rs11830037	A	0.08	0.040	0.006	3.42E-13	53
TSH	rs182307407	A	0.03	0.052	0.009	7.07E-09	33

TSH	rs7315310	A	0.40	-0.020	0.003	1.25E-11	46
TSH	rs7955258	A	0.42	-0.047	0.003	1.97E-54	249
TSH	rs7975994	C	0.92	0.040	0.005	6.09E-14	56
TSH	rs12826041	A	0.37	-0.018	0.003	3.67E-09	34
TSH	rs71447494	A	0.84	-0.027	0.004	1.07E-10	42
TSH	rs11836059	A	0.74	-0.019	0.003	2.15E-08	31
TSH	rs149363012	T	0.02	0.125	0.012	1.90E-26	114
TSH	rs61916657	A	0.32	-0.019	0.003	1.18E-09	36
TSH	rs10878984	T	0.35	-0.021	0.003	2.08E-12	50
TSH	rs61938844	A	0.03	0.172	0.010	5.52E-71	315
TSH	rs10735341	A	0.13	-0.042	0.004	1.52E-22	94
TSH	rs9521826	A	0.73	0.025	0.003	1.88E-14	57
TSH	rs17462267	A	0.73	-0.037	0.003	6.08E-29	122
TSH	rs9537312	T	0.21	-0.024	0.004	4.37E-11	43
TSH	rs10914681	T	0.25	0.023	0.003	2.34E-11	44
TSH	rs9532662	T	0.31	0.019	0.003	3.17E-09	36
TSH	rs568995	A	0.21	0.034	0.004	7.42E-22	94
TSH	rs4885687	T	0.73	0.026	0.003	5.96E-15	61
TSH	rs12889167	T	0.55	0.039	0.003	1.20E-40	181
TSH	rs12436555	A	0.16	-0.022	0.004	3.54E-08	31
TSH	rs6680638	T	0.47	-0.016	0.003	4.08E-08	30
TSH	rs7145546	C	0.55	-0.035	0.003	7.00E-34	148
TSH	rs148291250	A	0.99	0.137	0.019	1.33E-12	50
TSH	rs116909374	T	0.04	-0.159	0.009	1.11E-78	349
TSH	rs12891811	T	0.58	-0.022	0.003	1.67E-13	53
TSH	rs71405769	A	0.03	0.057	0.008	6.16E-12	48
TSH	rs117266160	A	0.03	-0.060	0.009	1.46E-11	46
TSH	rs61981267	T	0.02	0.072	0.013	9.88E-09	33
TSH	rs10132220	A	0.07	-0.045	0.006	2.61E-15	63
TSH	rs78218384	T	0.91	-0.066	0.005	1.01E-38	173
TSH	rs2234919	A	0.06	-0.053	0.006	2.71E-18	77
TSH	rs75235317	A	0.06	0.049	0.006	5.13E-15	61
TSH	rs8022931	C	0.54	-0.049	0.003	1.58E-62	280
TSH	rs117655019	T	0.03	-0.082	0.010	6.11E-17	70
TSH	rs2371566	T	0.08	-0.044	0.005	2.55E-16	67
TSH	rs112123737	T	0.06	-0.038	0.006	3.89E-09	34
TSH	rs77303590	T	0.03	0.068	0.010	1.72E-12	49
TSH	rs2295727	A	0.93	-0.033	0.006	1.51E-08	32
TSH	rs768356	T	0.80	-0.074	0.004	1.35E-92	424
TSH	rs12567744	A	0.68	0.036	0.003	1.15E-30	135
TSH	rs78218421	T	0.06	0.044	0.006	9.05E-13	50
TSH	rs1288492	T	0.46	0.019	0.003	2.94E-10	41
TSH	rs1044474	A	0.55	0.026	0.003	9.71E-19	78
TSH	rs17477923	T	0.75	0.061	0.003	7.59E-75	341

TSH	rs6494466	A	0.26	-0.020	0.003	2.08E-09	36
TSH	rs11857151	C	0.68	0.049	0.003	6.56E-57	250
TSH	rs30233	A	0.57	-0.032	0.003	1.53E-27	114
TSH	rs334699	A	0.06	-0.145	0.007	6.89E-98	440
TSH	rs17305041	A	0.92	0.036	0.006	3.07E-09	35
TSH	rs7203093	A	0.91	-0.038	0.005	8.47E-14	55
TSH	rs11641216	A	0.61	0.016	0.003	4.96E-08	29
TSH	rs1045476	A	0.18	0.043	0.004	2.55E-29	124
TSH	rs4785953	A	0.87	-0.032	0.004	7.54E-14	57
TSH	rs524770	A	0.39	0.023	0.003	2.89E-15	61
TSH	rs57186408	T	0.03	0.049	0.009	2.11E-08	31
TSH	rs73575083	A	0.67	0.092	0.003	3.03E-199	887
TSH	rs11864793	C	0.91	-0.044	0.006	1.80E-14	60
TSH	rs117544538	A	0.94	0.036	0.006	1.85E-08	32
TSH	rs12036629	A	0.53	-0.032	0.003	1.25E-27	119
TSH	rs55938136	A	0.78	-0.038	0.004	1.50E-23	99
TSH	rs199528	T	0.20	0.040	0.004	4.99E-29	125
TSH	rs35073649	T	0.38	0.025	0.003	4.56E-17	68
TSH	rs113599227	T	0.04	-0.084	0.007	9.31E-30	128
TSH	rs1801690	C	0.94	0.056	0.006	3.18E-19	80
TSH	rs9889941	A	0.70	0.018	0.003	7.30E-09	33
TSH	rs1042673	A	0.54	-0.043	0.003	9.41E-50	220
TSH	rs4793439	T	0.47	0.041	0.003	3.57E-45	197
TSH	rs879736	T	0.17	0.026	0.004	4.24E-11	44
TSH	rs112719042	C	0.24	-0.021	0.004	3.02E-08	31
TSH	rs6567094	A	0.55	0.031	0.003	2.22E-26	114
TSH	rs72978712	T	0.79	-0.038	0.004	3.79E-22	96
TSH	rs1496627	A	0.58	0.021	0.003	5.31E-13	53
TSH	rs11670562	T	0.74	-0.020	0.003	2.92E-09	35
TSH	rs4807021	A	0.30	0.024	0.003	7.64E-14	57
TSH	rs76978239	T	0.09	-0.031	0.005	1.00E-08	33
TSH	rs11672947	T	0.39	-0.024	0.003	1.36E-14	58
TSH	rs3848573	T	0.81	0.032	0.004	1.28E-17	73
TSH	rs7248104	A	0.41	-0.058	0.003	3.20E-87	379
TSH	rs12974053	T	0.04	0.048	0.007	6.45E-11	42
TSH	rs141735698	A	0.96	0.076	0.009	7.50E-19	79
TSH	rs1203930	A	0.22	-0.065	0.004	1.08E-78	347
TSH	rs1883806	T	0.39	0.020	0.003	5.21E-10	38
TSH	rs28482886	A	0.71	0.026	0.003	1.53E-15	63
TSH	rs139242164	T	0.01	0.116	0.016	2.55E-13	54
TSH	rs55830103	T	0.82	-0.028	0.004	2.52E-12	50
TSH	rs237080	T	0.29	-0.023	0.003	5.99E-12	46
TSH	rs6133344	T	0.51	-0.024	0.003	1.43E-16	68
TSH	rs11675342	T	0.42	0.028	0.003	1.34E-21	94

TSH	rs79259951	A	0.93	0.037	0.006	1.39E-10	41
TSH	rs6414089	A	0.36	0.018	0.003	6.44E-09	34
TSH	rs4471862	A	0.32	-0.025	0.003	2.40E-15	64
TSH	rs3792198	A	0.56	0.017	0.003	7.69E-09	34
TSH	rs62114518	T	0.31	-0.020	0.003	3.98E-09	35
TSH	rs28484879	T	0.92	0.031	0.005	7.83E-09	33
TSH	rs2288188	T	0.96	0.072	0.008	6.16E-20	83
TSH	rs2364727	T	0.11	0.027	0.005	1.30E-08	32
TSH	rs4340549	T	0.76	-0.023	0.004	1.45E-11	44
TSH	rs4274624	T	0.77	-0.020	0.004	9.34E-09	34
TSH	rs62189580	C	0.60	0.018	0.003	4.36E-09	35
TSH	rs56092701	T	0.09	0.045	0.005	7.79E-19	78
TSH	rs737308	T	0.28	-0.093	0.003	1.02E-177	786
TSH	rs6724073	T	0.73	0.051	0.004	3.35E-48	209
TSH	rs7589228	A	0.63	0.026	0.003	2.43E-18	77
TSH	rs5997969	T	0.33	0.025	0.003	3.99E-16	66
TSH	rs62192963	A	0.15	0.032	0.004	6.44E-15	60
TSH	rs113360717	T	0.03	0.081	0.008	1.38E-23	101
TSH	rs10186921	T	0.56	0.038	0.003	1.05E-38	173
TSH	rs6546566	T	0.69	0.018	0.003	6.65E-09	34
TSH	rs1661584	T	0.75	-0.048	0.003	2.66E-48	214
TSH	rs2439737	T	0.51	-0.017	0.003	4.63E-09	34
TSH	rs55717031	T	0.31	-0.020	0.003	4.94E-10	39
TSH	rs9857136	A	0.42	-0.032	0.003	1.63E-27	120
TSH	rs7651090	A	0.69	0.048	0.003	2.38E-52	236
TSH	rs59381142	A	0.24	-0.050	0.004	1.03E-46	207
TSH	rs6805350	T	0.94	-0.035	0.006	2.95E-08	31
TSH	rs115044883	A	0.08	-0.033	0.006	1.19E-08	33
TSH	rs17450274	A	0.29	0.027	0.003	8.33E-17	69
TSH	rs115371738	A	0.02	0.062	0.011	7.58E-09	34
TSH	rs13138273	A	0.80	0.111	0.004	1.00E-200	895
TSH	rs76697438	A	0.01	-0.104	0.015	4.75E-12	48
TSH	rs524424	A	0.56	-0.018	0.003	2.23E-09	35
TSH	rs1385737	T	0.13	-0.036	0.005	2.47E-14	59
TSH	rs11732564	A	0.28	-0.025	0.003	6.50E-15	59
TSH	rs1458819	T	0.89	0.028	0.005	6.34E-09	33
TSH	rs155943	A	0.47	0.019	0.003	4.11E-11	43
TSH	rs77994712	C	0.94	0.062	0.006	6.13E-22	94
TSH	rs6898408	C	0.34	0.018	0.003	7.57E-09	33
TSH	rs28567918	T	0.80	-0.022	0.004	2.19E-08	31
TSH	rs440225	A	0.71	-0.022	0.004	3.07E-08	31
TSH	rs62363038	A	0.77	0.026	0.003	3.90E-14	58
TSH	rs10075093	T	0.21	-0.028	0.004	7.38E-15	61
TSH	rs7707827	A	0.10	0.035	0.005	9.20E-12	46

TSH	rs115827777	T	0.02	0.096	0.012	7.61E-17	70
TSH	rs79211479	C	0.97	-0.068	0.009	5.25E-13	52
TSH	rs2928167	A	0.86	0.133	0.004	1.00E-200	957
TSH	rs9687206	A	0.57	-0.136	0.003	1.00E-200	2183
TSH	rs151297408	T	0.01	-0.091	0.014	1.50E-10	41
TSH	rs142891552	T	0.02	0.069	0.011	2.57E-10	40
TSH	rs148079416	C	0.98	0.085	0.012	5.95E-13	52
TSH	rs13436203	A	0.97	-0.069	0.008	9.80E-17	69
TSH	rs16874254	T	0.06	-0.065	0.007	1.28E-22	95
TSH	rs4273585	A	0.44	-0.043	0.003	3.11E-48	215
TSH	rs115236194	A	0.98	-0.066	0.012	1.48E-08	32
TSH	rs1969151	T	0.21	0.020	0.004	1.24E-08	33
TSH	rs1114707	T	0.34	0.021	0.003	3.03E-11	45
TSH	rs6901783	A	0.74	-0.019	0.003	6.79E-09	33
TSH	rs9497965	T	0.40	0.033	0.003	9.03E-29	128
TSH	rs79708723	A	0.03	0.068	0.009	5.14E-14	57
TSH	rs7758026	C	0.88	0.050	0.005	3.14E-28	119
TSH	rs73022136	T	0.03	0.072	0.010	2.91E-12	48
TSH	rs56228667	T	0.85	0.097	0.004	3.98E-122	557
TSH	rs1033701	A	0.27	-0.117	0.003	1.00E-200	1332
TSH	rs117801821	C	0.97	-0.068	0.011	1.27E-09	37
TSH	rs146760552	T	0.02	0.082	0.014	9.20E-09	33
TSH	rs12199716	A	0.02	0.109	0.014	4.99E-16	65
TSH	rs375911171	T	0.02	0.081	0.014	3.68E-09	35
TSH	rs34471367	A	0.96	-0.043	0.008	3.22E-08	31
TSH	rs9364808	T	0.92	0.033	0.006	4.84E-09	35
TSH	rs893310	T	0.53	-0.021	0.003	6.08E-13	51
TSH	rs62425402	T	0.11	-0.030	0.005	1.12E-10	41
TSH	rs751171	T	0.66	-0.036	0.003	9.06E-31	133
TSH	rs9396865	T	0.84	-0.030	0.004	4.57E-14	58
TSH	rs16895218	A	0.94	0.041	0.007	2.82E-10	39
TSH	rs1265091	T	0.18	0.045	0.004	2.79E-29	126
TSH	rs9461666	T	0.97	-0.067	0.011	5.14E-10	39
TSH	rs1129735	T	0.33	0.039	0.003	2.01E-30	134
TSH	rs184252375	T	0.29	0.038	0.005	4.11E-14	56
TSH	rs2396083	C	0.68	0.097	0.003	1.00E-200	921
TSH	rs34046483	A	0.08	0.078	0.006	4.12E-41	179
TSH	rs77547428	T	0.98	-0.105	0.015	4.85E-13	52
TSH	rs66760320	T	0.25	-0.078	0.003	7.94E-120	528
TSH	rs2031365	T	0.28	0.049	0.003	5.37E-52	231
TSH	rs62621812	A	0.03	0.080	0.010	2.08E-16	68
TSH	rs55837101	T	0.22	0.020	0.004	1.75E-08	32
TSH	rs706024	A	0.61	-0.020	0.003	3.16E-11	44
TSH	rs4318967	T	0.59	0.019	0.003	3.84E-10	38

TSH	rs10950803	A	0.55	0.018	0.003	2.82E-09	36
TSH	rs700750	A	0.62	0.031	0.003	3.15E-25	105
TSH	rs1875057	A	0.37	0.017	0.003	8.74E-09	33
TSH	rs10263288	A	0.89	-0.032	0.005	5.37E-12	48
TSH	rs3134107	A	0.18	-0.023	0.004	1.11E-09	37
TSH	rs72682433	T	0.90	-0.035	0.005	2.32E-13	53
TSH	rs78404781	A	0.99	0.183	0.016	2.86E-32	140
TSH	rs114322847	T	0.03	-0.161	0.010	2.84E-61	275
TSH	rs28640053	A	0.17	0.028	0.004	2.94E-13	52
TSH	rs56009477	A	0.85	0.052	0.004	2.62E-38	169
TSH	rs66963240	T	0.43	-0.038	0.003	6.98E-40	174
TSH	rs73234168	A	0.26	-0.061	0.003	1.43E-75	336
TSH	rs62506639	T	0.81	-0.027	0.004	1.47E-13	54
TSH	rs9772642	T	0.70	-0.029	0.003	1.84E-19	79
TSH	rs67639219	A	0.33	-0.020	0.003	4.44E-11	43
TSH	rs396483	T	0.30	0.021	0.003	7.73E-11	41
TSH	rs2921053	C	0.46	-0.025	0.003	1.48E-16	66
TSH	rs17729624	A	0.10	0.034	0.005	1.72E-12	50
TSH	rs925489	T	0.65	0.075	0.003	4.50E-138	623
TSH	rs182020360	T	0.98	-0.064	0.011	5.84E-09	34
TSH	rs4743168	A	0.63	-0.017	0.003	2.47E-08	31
TSH	rs10818666	T	0.73	-0.019	0.003	9.46E-09	33
TSH	rs954585	A	0.65	-0.020	0.003	3.04E-11	44
TSH	rs7853472	A	0.38	0.028	0.003	2.51E-21	93
TSH	rs734638	C	0.70	0.020	0.003	6.50E-10	37
TSH	rs554833	T	0.35	0.042	0.003	8.54E-44	194
TSH	rs4917375	A	0.44	0.017	0.003	1.74E-08	32
TSH	rs9298749	A	0.61	-0.032	0.003	3.50E-26	112
TSH	rs10814737	T	0.95	-0.054	0.007	2.98E-14	58
TSH	rs806028	A	0.08	0.029	0.005	4.87E-08	30
TSH	rs17209669	T	0.25	0.025	0.003	3.28E-14	58
TSH	rs7864565	A	0.41	-0.036	0.003	1.56E-34	153
TSH	rs10122640	A	0.09	0.036	0.005	1.13E-12	50
TSH	rs10814915	T	0.44	0.052	0.003	8.80E-70	317
TSH	rs4741961	T	0.84	-0.033	0.004	5.02E-16	64
TSH	rs7875193	T	0.35	0.017	0.003	4.08E-08	29
TSH	rs13286806	A	0.42	-0.018	0.003	1.42E-09	36
Hypothyroidism	rs10119187	T	0.82	-0.054	0.007	5.48E-14	57
Hypothyroidism	rs10180164	A	0.97	-0.088	0.016	4.27E-08	30
Hypothyroidism	rs10407062	T	0.54	0.037	0.006	3.24E-11	44
Hypothyroidism	rs1042127	A	0.82	0.048	0.008	1.05E-08	33
Hypothyroidism	rs1042140	A	0.77	-0.039	0.007	8.58E-09	33
Hypothyroidism	rs1050979	A	0.50	-0.039	0.006	3.24E-12	49
Hypothyroidism	rs10735341	A	0.17	-0.047	0.008	2.04E-08	31

Hypothyroidism	rs1074849	G	0.77	-0.043	0.007	8.29E-11	42
Hypothyroidism	rs10748781	C	0.44	0.060	0.006	1.73E-24	104
Hypothyroidism	rs10751648	T	0.43	0.034	0.006	1.97E-09	36
Hypothyroidism	rs10753774	C	0.71	-0.032	0.006	4.39E-08	30
Hypothyroidism	rs10768126	A	0.60	-0.043	0.006	1.07E-13	55
Hypothyroidism	rs10775303	G	0.48	0.031	0.006	4.49E-08	30
Hypothyroidism	rs1079418	A	0.69	0.102	0.006	9.00E-61	270
Hypothyroidism	rs10814915	T	0.44	0.063	0.006	3.26E-29	126
Hypothyroidism	rs10818037	G	0.44	-0.181	0.006	1.00E-200	916
Hypothyroidism	rs10887774	G	0.73	0.043	0.006	3.09E-12	49
Hypothyroidism	rs10905430	C	0.58	0.048	0.006	1.79E-15	63
Hypothyroidism	rs10910095	G	0.86	-0.051	0.007	3.58E-12	48
Hypothyroidism	rs10917470	G	0.50	-0.096	0.006	4.35E-64	286
Hypothyroidism	rs11073716	C	0.62	0.034	0.006	1.53E-09	36
Hypothyroidism	rs11181289	C	0.62	0.033	0.006	4.54E-08	30
Hypothyroidism	rs11185131	A	0.47	0.037	0.006	3.06E-09	35
Hypothyroidism	rs112165453	C	0.97	-0.116	0.019	1.44E-09	37
Hypothyroidism	rs112614658	A	0.94	-0.068	0.012	1.14E-08	33
Hypothyroidism	rs112880194	G	0.96	0.090	0.016	7.56E-09	33
Hypothyroidism	rs113135100	G	0.87	0.049	0.009	4.02E-08	30
Hypothyroidism	rs113170275	G	0.68	0.037	0.006	3.89E-09	35
Hypothyroidism	rs113317416	C	0.88	-0.104	0.011	8.52E-22	92
Hypothyroidism	rs113866322	T	0.63	0.040	0.006	3.36E-12	48
Hypothyroidism	rs114786190	G	0.92	-0.067	0.011	4.80E-10	39
Hypothyroidism	rs11603616	G	0.68	0.033	0.006	4.02E-08	30
Hypothyroidism	rs11666808	T	0.38	-0.033	0.006	4.58E-08	30
Hypothyroidism	rs11675342	C	0.58	-0.154	0.006	7.18E-159	721
Hypothyroidism	rs11683914	T	0.46	0.057	0.006	7.45E-22	92
Hypothyroidism	rs11687741	C	0.65	0.038	0.006	4.52E-11	43
Hypothyroidism	rs116909374	C	0.97	0.143	0.019	9.68E-14	55
Hypothyroidism	rs11718237	G	0.56	0.036	0.006	3.08E-10	40
Hypothyroidism	rs11724128	T	0.69	-0.038	0.007	3.74E-08	30
Hypothyroidism	rs117591891	C	0.98	-0.158	0.025	1.86E-10	41
Hypothyroidism	rs117744081	A	0.97	-0.089	0.016	2.13E-08	31
Hypothyroidism	rs11830037	C	0.92	-0.063	0.011	5.09E-09	34
Hypothyroidism	rs12046347	G	0.41	-0.035	0.006	2.42E-09	36
Hypothyroidism	rs12134155	C	0.37	0.047	0.006	4.95E-16	66
Hypothyroidism	rs12144309	C	0.81	0.044	0.007	6.20E-11	43
Hypothyroidism	rs12187443	T	0.68	0.041	0.006	2.14E-11	45
Hypothyroidism	rs12357667	G	0.59	0.048	0.006	1.03E-16	69
Hypothyroidism	rs1239704	G	0.20	0.047	0.007	2.86E-11	44
Hypothyroidism	rs12636552	A	0.69	-0.032	0.006	3.94E-08	30
Hypothyroidism	rs12893151	C	0.79	0.044	0.007	4.99E-10	39
Hypothyroidism	rs12904211	T	0.66	0.039	0.006	2.78E-10	40

Hypothyroidism	rs12928973	C	0.89	0.049	0.009	2.97E-08	31
Hypothyroidism	rs12937081	A	0.86	0.050	0.008	5.29E-10	39
Hypothyroidism	rs12952267	G	0.76	0.038	0.007	1.68E-08	32
Hypothyroidism	rs13075906	C	0.60	0.074	0.006	4.87E-35	153
Hypothyroidism	rs1317085	C	0.67	0.035	0.006	3.21E-08	31
Hypothyroidism	rs1320344	A	0.31	0.036	0.006	2.38E-10	40
Hypothyroidism	rs13209358	C	0.92	-0.092	0.010	1.05E-19	83
Hypothyroidism	rs13250295	C	0.70	0.045	0.006	5.42E-13	52
Hypothyroidism	rs13399762	A	0.95	-0.071	0.012	6.64E-09	34
Hypothyroidism	rs142127908	A	0.47	0.048	0.008	4.78E-09	34
Hypothyroidism	rs142647938	C	0.98	0.124	0.022	1.97E-08	32
Hypothyroidism	rs143104579	G	0.98	0.128	0.023	1.45E-08	32
Hypothyroidism	rs145124960	C	0.98	-0.140	0.022	2.42E-10	40
Hypothyroidism	rs145269503	G	0.98	0.116	0.020	6.88E-09	34
Hypothyroidism	rs1470579	A	0.68	0.065	0.006	1.10E-28	123
Hypothyroidism	rs147563297	G	0.99	-0.197	0.024	8.09E-17	69
Hypothyroidism	rs147610574	T	0.96	-0.086	0.015	3.52E-09	35
Hypothyroidism	rs1479567	G	0.62	-0.125	0.006	2.00E-102	462
Hypothyroidism	rs148554863	T	0.98	0.205	0.022	7.09E-20	83
Hypothyroidism	rs149875147	G	0.93	-0.066	0.012	1.36E-08	32
Hypothyroidism	rs151190323	C	0.91	-0.061	0.010	8.49E-10	38
Hypothyroidism	rs151213404	G	0.61	0.062	0.006	6.90E-26	111
Hypothyroidism	rs151233	C	0.87	-0.068	0.008	4.89E-16	66
Hypothyroidism	rs1534424	A	0.61	0.055	0.006	1.49E-20	86
Hypothyroidism	rs1567124	C	0.70	-0.036	0.006	2.08E-08	31
Hypothyroidism	rs16899682	G	0.98	-0.120	0.018	1.81E-11	45
Hypothyroidism	rs1702877	C	0.68	-0.050	0.006	3.63E-17	71
Hypothyroidism	rs17111361	T	0.88	0.058	0.008	5.01E-12	48
Hypothyroidism	rs17194601	T	0.87	0.054	0.009	4.59E-09	34
Hypothyroidism	rs17494319	C	0.79	0.042	0.007	1.18E-08	33
Hypothyroidism	rs17664732	T	0.73	0.069	0.006	4.00E-27	116
Hypothyroidism	rs17676218	C	0.77	0.037	0.007	2.79E-08	31
Hypothyroidism	rs177055	G	0.36	-0.033	0.006	7.19E-09	33
Hypothyroidism	rs17770799	T	0.85	0.073	0.008	8.16E-21	88
Hypothyroidism	rs185985460	C	0.98	-0.134	0.020	2.01E-11	45
Hypothyroidism	rs1868167	A	0.22	0.055	0.007	4.18E-16	66
Hypothyroidism	rs189508974	T	0.97	0.102	0.018	1.75E-08	32
Hypothyroidism	rs192874879	G	0.98	-0.155	0.025	6.96E-10	38
Hypothyroidism	rs196047	A	0.39	-0.032	0.006	4.32E-08	30
Hypothyroidism	rs1998616	A	0.72	-0.041	0.006	2.87E-12	49
Hypothyroidism	rs2016105	G	0.98	-0.260	0.024	1.04E-26	114
Hypothyroidism	rs2056625	G	0.61	-0.033	0.006	4.73E-08	30
Hypothyroidism	rs2061914	A	0.91	-0.059	0.010	1.42E-09	37
Hypothyroidism	rs2111485	A	0.40	-0.039	0.006	1.73E-11	45

Hypothyroidism	rs2160042	G	0.87	0.052	0.009	1.71E-09	36
Hypothyroidism	rs2250179	T	0.43	-0.036	0.006	8.14E-10	38
Hypothyroidism	rs229540	T	0.58	-0.051	0.006	3.46E-19	80
Hypothyroidism	rs2297163	T	0.81	-0.046	0.007	3.81E-11	44
Hypothyroidism	rs2314345	G	0.37	0.094	0.006	5.60E-59	262
Hypothyroidism	rs2374589	A	0.93	-0.061	0.011	3.07E-08	31
Hypothyroidism	rs2391836	C	0.73	0.068	0.006	1.15E-25	110
Hypothyroidism	rs2435204	A	0.77	-0.088	0.007	3.05E-39	172
Hypothyroidism	rs244689	A	0.14	0.054	0.008	2.86E-11	44
Hypothyroidism	rs2456451	G	0.66	0.070	0.006	6.23E-33	143
Hypothyroidism	rs2472540	G	0.54	-0.063	0.006	9.38E-29	124
Hypothyroidism	rs263660	A	0.72	0.036	0.006	2.28E-08	31
Hypothyroidism	rs2654188	T	0.39	0.051	0.006	3.12E-16	67
Hypothyroidism	rs2675662	A	0.58	0.032	0.006	4.03E-09	35
Hypothyroidism	rs2706119	A	0.10	0.054	0.010	3.53E-08	30
Hypothyroidism	rs2761063	C	0.48	-0.068	0.006	1.04E-32	142
Hypothyroidism	rs2783971	A	0.47	-0.044	0.006	7.05E-14	56
Hypothyroidism	rs2811985	C	0.31	-0.039	0.006	2.52E-09	36
Hypothyroidism	rs28558845	G	0.84	-0.052	0.007	2.46E-12	49
Hypothyroidism	rs28646006	C	0.79	0.100	0.009	1.31E-31	137
Hypothyroidism	rs2911460	A	0.31	0.036	0.006	8.78E-09	33
Hypothyroidism	rs2921053	G	0.55	0.071	0.006	6.57E-33	143
Hypothyroidism	rs2928166	T	0.87	0.070	0.009	5.64E-16	66
Hypothyroidism	rs30233	G	0.44	0.038	0.006	1.99E-10	40
Hypothyroidism	rs3087243	G	0.55	0.125	0.006	4.47E-109	492
Hypothyroidism	rs3134996	A	0.36	-0.145	0.007	3.37E-98	442
Hypothyroidism	rs3184504	T	0.49	0.132	0.006	2.19E-121	549
Hypothyroidism	rs329124	A	0.58	0.037	0.006	8.01E-10	38
Hypothyroidism	rs334719	A	0.07	-0.130	0.014	9.15E-20	83
Hypothyroidism	rs34048763	G	0.71	0.043	0.007	5.45E-11	43
Hypothyroidism	rs34292522	A	0.69	-0.048	0.006	8.85E-15	60
Hypothyroidism	rs34357460	G	0.67	0.054	0.006	5.90E-17	70
Hypothyroidism	rs34536443	G	0.95	0.084	0.013	2.86E-10	40
Hypothyroidism	rs34666276	T	0.63	-0.037	0.006	1.50E-10	41
Hypothyroidism	rs34787248	C	0.83	0.094	0.011	1.06E-17	73
Hypothyroidism	rs35000415	C	0.89	-0.053	0.009	2.60E-09	35
Hypothyroidism	rs35004101	T	0.96	0.096	0.016	2.67E-09	35
Hypothyroidism	rs35074907	G	0.98	-0.155	0.020	1.92E-14	59
Hypothyroidism	rs35484831	C	0.95	-0.071	0.013	2.66E-08	31
Hypothyroidism	rs369340113	G	0.56	0.065	0.008	5.44E-18	75
Hypothyroidism	rs3755397	A	0.90	0.061	0.009	1.12E-10	42
Hypothyroidism	rs3761959	C	0.56	-0.047	0.006	1.28E-16	68
Hypothyroidism	rs3796220	C	0.79	-0.043	0.007	1.26E-09	37
Hypothyroidism	rs3802214	T	0.19	0.048	0.007	6.31E-11	43

Hypothyroidism	rs3821374	A	0.23	0.045	0.008	3.43E-09	35
Hypothyroidism	rs3826884	G	0.82	-0.044	0.007	9.46E-10	37
Hypothyroidism	rs3850765	T	0.42	-0.034	0.006	3.37E-09	35
Hypothyroidism	rs41295121	C	0.99	0.191	0.030	1.64E-10	41
Hypothyroidism	rs41444548	C	0.93	0.103	0.011	1.01E-19	83
Hypothyroidism	rs4269168	C	0.53	-0.045	0.005	9.68E-17	69
Hypothyroidism	rs4409785	T	0.82	-0.069	0.007	2.59E-22	94
Hypothyroidism	rs4478401	G	0.62	0.039	0.006	1.63E-10	41
Hypothyroidism	rs4664302	T	0.22	-0.040	0.007	5.81E-09	34
Hypothyroidism	rs4679083	T	0.58	-0.034	0.006	5.80E-09	34
Hypothyroidism	rs4684098	G	0.27	0.085	0.007	1.14E-36	160
Hypothyroidism	rs4690362	A	0.14	-0.093	0.010	2.76E-22	94
Hypothyroidism	rs4742719	G	0.73	-0.055	0.006	4.39E-19	80
Hypothyroidism	rs4743034	G	0.77	0.052	0.007	9.52E-15	60
Hypothyroidism	rs4760332	C	0.69	0.034	0.006	7.18E-09	33
Hypothyroidism	rs4804416	T	0.58	-0.088	0.006	5.27E-55	244
Hypothyroidism	rs4820946	C	0.24	0.047	0.007	2.05E-12	49
Hypothyroidism	rs4844563	A	0.67	-0.034	0.006	6.01E-09	34
Hypothyroidism	rs4915077	T	0.92	-0.238	0.010	5.80E-133	602
Hypothyroidism	rs4933466	A	0.60	0.051	0.006	7.03E-18	74
Hypothyroidism	rs496201	C	0.49	0.031	0.005	2.28E-08	31
Hypothyroidism	rs4963198	G	0.36	-0.033	0.006	1.66E-08	32
Hypothyroidism	rs535850137	G	0.62	-0.059	0.009	5.54E-11	43
Hypothyroidism	rs557444314	A	0.98	-0.197	0.030	8.01E-11	42
Hypothyroidism	rs559734662	T	0.93	0.083	0.014	1.16E-09	37
Hypothyroidism	rs56249713	T	0.58	0.036	0.006	1.33E-10	41
Hypothyroidism	rs564419538	G	0.80	-0.061	0.010	2.49E-09	36
Hypothyroidism	rs56822079	C	0.88	0.050	0.009	2.87E-08	31
Hypothyroidism	rs5750617	T	0.70	-0.042	0.006	8.28E-11	42
Hypothyroidism	rs58288624	T	0.92	0.076	0.013	5.26E-09	34
Hypothyroidism	rs58397833	A	0.67	-0.037	0.006	1.06E-09	37
Hypothyroidism	rs58554773	G	0.82	0.042	0.007	9.49E-09	33
Hypothyroidism	rs587611953	C	0.82	-0.057	0.009	3.34E-11	44
Hypothyroidism	rs59381142	G	0.76	0.046	0.006	5.70E-13	52
Hypothyroidism	rs6036117	A	0.72	0.043	0.007	4.50E-10	39
Hypothyroidism	rs60389750	C	0.68	-0.038	0.007	1.65E-08	32
Hypothyroidism	rs6081210	A	0.20	-0.041	0.007	2.91E-08	31
Hypothyroidism	rs62226440	G	0.71	0.054	0.006	9.05E-18	74
Hypothyroidism	rs62476228	A	0.75	-0.054	0.007	4.03E-13	53
Hypothyroidism	rs62560473	G	0.96	0.214	0.019	1.14E-29	128
Hypothyroidism	rs62621812	G	0.98	-0.105	0.018	8.51E-09	33
Hypothyroidism	rs6415788	G	0.39	0.036	0.006	1.12E-08	33
Hypothyroidism	rs6679677	C	0.90	-0.215	0.009	6.17E-123	556
Hypothyroidism	rs6733824	G	0.44	0.031	0.006	1.26E-08	32

Hypothyroidism	rs67412685	G	0.82	-0.043	0.008	1.08E-08	33
Hypothyroidism	rs6748294	G	0.82	0.044	0.008	1.05E-08	33
Hypothyroidism	rs6751804	C	0.34	-0.049	0.006	1.14E-15	64
Hypothyroidism	rs6755639	A	0.49	-0.044	0.006	2.86E-15	62
Hypothyroidism	rs6892488	C	0.87	-0.051	0.008	6.06E-10	38
Hypothyroidism	rs6919204	C	0.78	-0.042	0.007	3.18E-09	35
Hypothyroidism	rs6923866	T	0.75	0.074	0.007	1.23E-25	110
Hypothyroidism	rs6996585	A	0.59	0.040	0.006	5.14E-12	48
Hypothyroidism	rs7017073	T	0.78	0.043	0.007	8.80E-09	33
Hypothyroidism	rs706778	C	0.59	-0.052	0.005	4.21E-22	93
Hypothyroidism	rs7100297	T	0.41	0.048	0.006	1.32E-17	73
Hypothyroidism	rs7101437	A	0.53	0.041	0.006	2.48E-13	54
Hypothyroidism	rs7128207	G	0.44	-0.056	0.006	5.57E-23	97
Hypothyroidism	rs71311695	T	0.28	0.040	0.007	1.15E-09	37
Hypothyroidism	rs71351111	G	0.65	-0.057	0.006	1.54E-19	82
Hypothyroidism	rs71508903	C	0.80	-0.055	0.007	1.02E-15	64
Hypothyroidism	rs7151424	G	0.55	0.059	0.006	3.33E-24	103
Hypothyroidism	rs7185291	C	0.38	-0.034	0.006	1.53E-08	32
Hypothyroidism	rs7228151	T	0.80	-0.061	0.007	8.71E-18	74
Hypothyroidism	rs7251	C	0.68	0.040	0.006	2.64E-11	44
Hypothyroidism	rs7254729	T	0.42	0.049	0.006	2.30E-17	72
Hypothyroidism	rs72682416	G	0.89	-0.052	0.009	1.89E-09	36
Hypothyroidism	rs72845719	T	0.88	0.119	0.010	8.50E-35	151
Hypothyroidism	rs7298096	G	0.45	0.031	0.006	4.81E-08	30
Hypothyroidism	rs7327740	T	0.73	-0.058	0.006	1.55E-21	91
Hypothyroidism	rs7329077	C	0.90	0.061	0.010	2.43E-10	40
Hypothyroidism	rs73575095	T	0.68	0.103	0.006	4.77E-61	272
Hypothyroidism	rs737308	T	0.28	-0.095	0.007	3.72E-47	208
Hypothyroidism	rs7417283	C	0.21	-0.063	0.008	1.23E-15	64
Hypothyroidism	rs74267890	G	0.98	0.143	0.022	9.17E-11	42
Hypothyroidism	rs742870	T	0.52	0.070	0.006	9.02E-32	138
Hypothyroidism	rs7444385	C	0.60	0.032	0.006	1.29E-08	32
Hypothyroidism	rs74745605	A	0.83	-0.063	0.008	9.73E-17	69
Hypothyroidism	rs748741	A	0.64	-0.063	0.006	3.69E-26	112
Hypothyroidism	rs751171	T	0.67	-0.042	0.006	8.00E-12	47
Hypothyroidism	rs75172178	T	0.56	-0.036	0.006	5.62E-09	34
Hypothyroidism	rs75258843	A	0.98	0.175	0.026	1.44E-11	46
Hypothyroidism	rs75438091	C	0.95	0.107	0.015	6.03E-13	52
Hypothyroidism	rs7568275	G	0.22	0.065	0.007	4.86E-23	98
Hypothyroidism	rs7570971	C	0.62	0.042	0.007	3.13E-09	35
Hypothyroidism	rs75848076	A	0.98	-0.124	0.020	3.41E-10	39
Hypothyroidism	rs75926384	C	0.99	0.287	0.046	3.06E-10	40
Hypothyroidism	rs7617081	A	0.34	0.035	0.006	7.39E-09	33
Hypothyroidism	rs76318082	C	0.90	-0.052	0.009	3.95E-08	30

Hypothyroidism	rs76428106	T	0.99	-0.179	0.027	5.82E-11	43
Hypothyroidism	rs7655915	C	0.81	0.095	0.007	1.96E-39	173
Hypothyroidism	rs77349713	T	0.94	0.078	0.012	1.56E-11	45
Hypothyroidism	rs7752035	T	0.95	-0.076	0.013	4.25E-09	35
Hypothyroidism	rs7754251	G	0.44	-0.069	0.006	1.30E-34	151
Hypothyroidism	rs7772305	A	0.69	-0.058	0.007	5.45E-17	70
Hypothyroidism	rs7799042	G	0.80	0.043	0.007	1.23E-09	37
Hypothyroidism	rs78432262	G	0.94	0.070	0.012	8.08E-09	33
Hypothyroidism	rs78533227	A	0.84	0.046	0.008	6.98E-09	34
Hypothyroidism	rs7853349	A	0.62	-0.146	0.006	4.56E-134	607
Hypothyroidism	rs78622464	C	0.90	0.053	0.010	3.97E-08	30
Hypothyroidism	rs7870475	T	0.52	0.033	0.006	3.56E-08	30
Hypothyroidism	rs7955141	A	0.44	-0.034	0.006	7.87E-09	33
Hypothyroidism	rs7955258	A	0.40	-0.038	0.006	1.63E-10	41
Hypothyroidism	rs7993585	T	0.49	-0.038	0.006	3.92E-11	44
Hypothyroidism	rs8013274	G	0.80	-0.112	0.007	8.99E-56	248
Hypothyroidism	rs8015224	T	0.69	0.037	0.006	5.49E-09	34
Hypothyroidism	rs8107162	T	0.57	0.032	0.006	9.39E-09	33
Hypothyroidism	rs836473	T	0.58	-0.034	0.006	6.67E-09	34
Hypothyroidism	rs855655	T	0.50	-0.037	0.007	2.04E-08	31
Hypothyroidism	rs871852	G	0.50	0.038	0.006	2.31E-11	45
Hypothyroidism	rs881858	G	0.30	-0.108	0.006	1.81E-69	310
Hypothyroidism	rs910634	G	0.51	-0.033	0.006	2.10E-09	36
Hypothyroidism	rs911760	C	0.78	-0.048	0.008	1.69E-08	32
Hypothyroidism	rs9298817	A	0.36	0.046	0.006	9.13E-14	56
Hypothyroidism	rs932036	A	0.70	-0.034	0.006	8.32E-09	33
Hypothyroidism	rs933392	T	0.17	0.063	0.008	6.46E-14	56
Hypothyroidism	rs9368695	C	0.98	0.119	0.021	1.58E-08	32
Hypothyroidism	rs9377117	T	0.72	-0.097	0.007	1.42E-49	219
Hypothyroidism	rs9380229	C	0.71	0.122	0.008	2.57E-48	213
Hypothyroidism	rs9457243	C	0.94	-0.080	0.011	1.06E-12	51
Hypothyroidism	rs9554561	C	0.73	0.042	0.006	8.47E-11	42
Hypothyroidism	rs9729284	C	0.28	-0.036	0.006	1.82E-09	36
Hypothyroidism	rs9821630	A	0.73	0.036	0.007	3.97E-08	30
Hypothyroidism	rs9878655	T	0.56	0.091	0.006	1.32E-50	224
Hypothyroidism	rs992105	C	0.17	-0.049	0.008	1.45E-10	41
Hypothyroidism	rs9926188	C	0.49	0.032	0.006	2.42E-08	31
Hypothyroidism	rs995734	G	0.24	-0.169	0.007	2.05E-121	549
Hyperthyroidism	rs2476601	G	0.89	-0.386	0.054	6.18E-13	52
Hyperthyroidism	rs1977710	G	0.44	0.146	0.022	5.19E-11	43
Hyperthyroidism	rs12612769	C	0.22	0.145	0.025	8.78E-09	33
Hyperthyroidism	rs231779	T	0.47	0.235	0.022	3.61E-26	112
Hyperthyroidism	rs6780858	G	0.47	-0.163	0.023	3.85E-13	53
Hyperthyroidism	rs13136820	T	0.70	-0.136	0.024	2.37E-08	31

Hyperthyroidism	rs73393463	C	0.03	-0.316	0.056	1.51E-08	32
Hyperthyroidism	rs3998799	G	0.40	0.317	0.026	1.21E-33	147
Hyperthyroidism	rs11758670	C	0.12	-0.486	0.035	3.36E-43	190
Hyperthyroidism	rs116749187	T	0.11	0.297	0.035	2.62E-17	72
Hyperthyroidism	rs75324027	G	0.28	0.344	0.024	4.18E-46	204
Hyperthyroidism	rs9357156	C	0.23	0.440	0.025	1.09E-68	307
Hyperthyroidism	rs7754251	C	0.49	0.131	0.023	7.16E-09	33
Hyperthyroidism	rs239933	G	0.48	0.135	0.022	6.73E-10	38
Hyperthyroidism	rs860262	A	0.42	-0.137	0.025	2.11E-08	31
Hyperthyroidism	rs2466028	C	0.36	-0.145	0.024	6.28E-10	38
Hyperthyroidism	rs10821944	T	0.70	-0.157	0.023	1.43E-11	46
Hyperthyroidism	rs4409785	C	0.14	0.203	0.035	6.81E-09	34
Hyperthyroidism	rs7135295	A	0.11	0.237	0.036	3.51E-11	44
Hyperthyroidism	rs28414437	C	0.42	0.287	0.023	1.98E-36	159
Hyperthyroidism	rs9929899	A	0.56	-0.224	0.039	1.31E-08	32
Hyperthyroidism	rs17689159	C	0.30	0.162	0.024	1.55E-11	46
Hyperthyroidism	rs1569723	A	0.71	0.164	0.023	1.62E-12	50
Hyperthyroidism	rs34544259	G	0.40	0.141	0.023	6.75E-10	38

EA, effect allele; EAF, effect allele frequency; TSH, thyroid-stimulating hormone.

Table S6. Association between hypothyroidism and metabolic dysfunction-associated steatotic liver disease (MASLD) histological subtypes by treating simple steatosis as the reference group

	Simple steatosis N=8144	MASH without fibrosis N=1399	Non-cirrhotic fibrosis N=1941	Cirrhosis N=688
Number, %	1.7	3.7	5.3	2.8
· Unadjusted HR (95% CI)	1 (ref.)	2.2 (1.58-3.05)	3.29 (2.52-4.29)	1.48 (0.91-2.42)
· Multivariable adjusted model (95% CI)	1 (ref.)	2.11 (1.42-3.12)	2.72 (2-3.7)	1.17 (0.61-2.24)

Abbreviations: CI, confidence interval; MASH, metabolic dysfunction-associated steatohepatitis; OR, odds ratio.
All models were conditioned on matching factors, including age, sex, biopsy calendar year, and county.
Multivariable adjusted model was adjusted for education, country at birth, metabolic disorders, autoimmune disease, chronic obstructive pulmonary disease, anemia, clinical visit in the past 12 months.

Table S7: Results of subgroup analyses

Stratified Models	Population Controls N=56,831	All MASLD N=12,172	Simple Steatosis N=8144	NASH without fibrosis N=1399	Non-Cirrhotic Fibrosis N=1941	Cirrhosis N=688
Male	1 (ref.)	3.1 (2.02-4.76)	2.76 (1.46-5.21)	1.22 (0.28-5.31)	4.56 (2.04-10.22)	4.15 (0.4-42.78)
Female	1 (ref.)	1.63 (1.3-2.04)	1.23 (0.89-1.71)	2.89 (1.53-5.47)	2.25 (1.49-3.41)	1.16 (0.41-3.29)
Age at index biopsy						
18-39 years	1 (ref.)	5.32 (2.29-12.34)	2.84 (0.76-10.65)	8.18 (0.45-149.8)	24.54 (2.85-210.88)	-
≥40 years	1 (ref.)	1.49 (1.19-1.85)	1.14 (0.83-1.56)	2.24 (1.22-4.11)	2.06 (1.38-3.07)	1.48 (0.55-3.98)

The association was adjusted for age, sex, index calendar year, county, education, metabolic disorders, autoimmune disease, chronic obstructive pulmonary disease, anemia, clinical visit in the past 12 months.

Table S8. Association between hypothyroidism and metabolic dysfunction-associated steatotic liver disease (MASLD) among MASLD cases and their sibling controls

	Sibling controls	All MASLD	Simple Steatosis	MASH without fibrosis	Non-Cirrhotic Fibrosis	Cirrhosis
	N=10,682	N=5478	N=3655	N=632	N=931	N=260
Number, %	1.6	3	1.9	5.1	5.9	3.5
Crude model, OR (95% CI)	1 (ref.)	2.30 (1.73-3.05)	1.93 (1.27-2.92)	2.23 (1.10-4.51)	3.76 (2.13-6.62)	1.52 (0.57-4.04)
Multivariable adjusted model, OR (95% CI)	1 (ref.)	2.08 (1.31-3.31)	1.52 (0.76-3.04)	1.51 (0.35-6.48)	7.94 (1.91-33)	0.81 (0.12-5.65)

Abbreviations: CI, confidence interval; MASH, metabolic dysfunction-associated steatohepatitis; OR, odds ratio.
Crude model was adjusted for age, sex, index calendar year (biopsy year for cases and matching year for sibling controls), and county. Multivariable adjusted model was adjusted for age, sex, index calendar year, county, education, metabolic disorders, autoimmune disease, chronic obstructive pulmonary disease, anemia, clinical visit in the past 12 months.

Table S9. Results of multivariable Mendelian randomization and mediation analyses

Exposure	Adjustment	Beta	SE	P	Mediation	Mediation_LB	Mediation_UB
TSH	None	0.093	0.032	0.004			
	BMI	0.088	0.033	0.007	5.38%	-89.01	99.77
	Type 2 diabetes	0.098	0.033	0.003	-5.38%	-104.81	94.06
	Triglycerides	0.075	0.033	0.024	19.35%	-68.93	107.64
	Systolic blood pressure	0.091	0.033	0.006	2.15%	-73.64	108.05
Hypothyroidism	None	0.072	0.021	0.001			
	BMI	0.070	0.021	0.001	2.78%	-76.95	82.51
	Type 2 diabetes	0.073	0.021	0.000	-1.39%	-82.80	80.02
	Triglycerides	0.065	0.021	0.002	9.72%	-67.29	86.74
	Systolic blood pressure	0.077	0.021	0.000	-6.94%	-75.01	83.35

LB, lower bound of 95% confidence interval; TSH, thyroid-stimulating hormone; UB, upper bound of 95% confidence interval.

Fig. S1. Flow chart of sample selection.

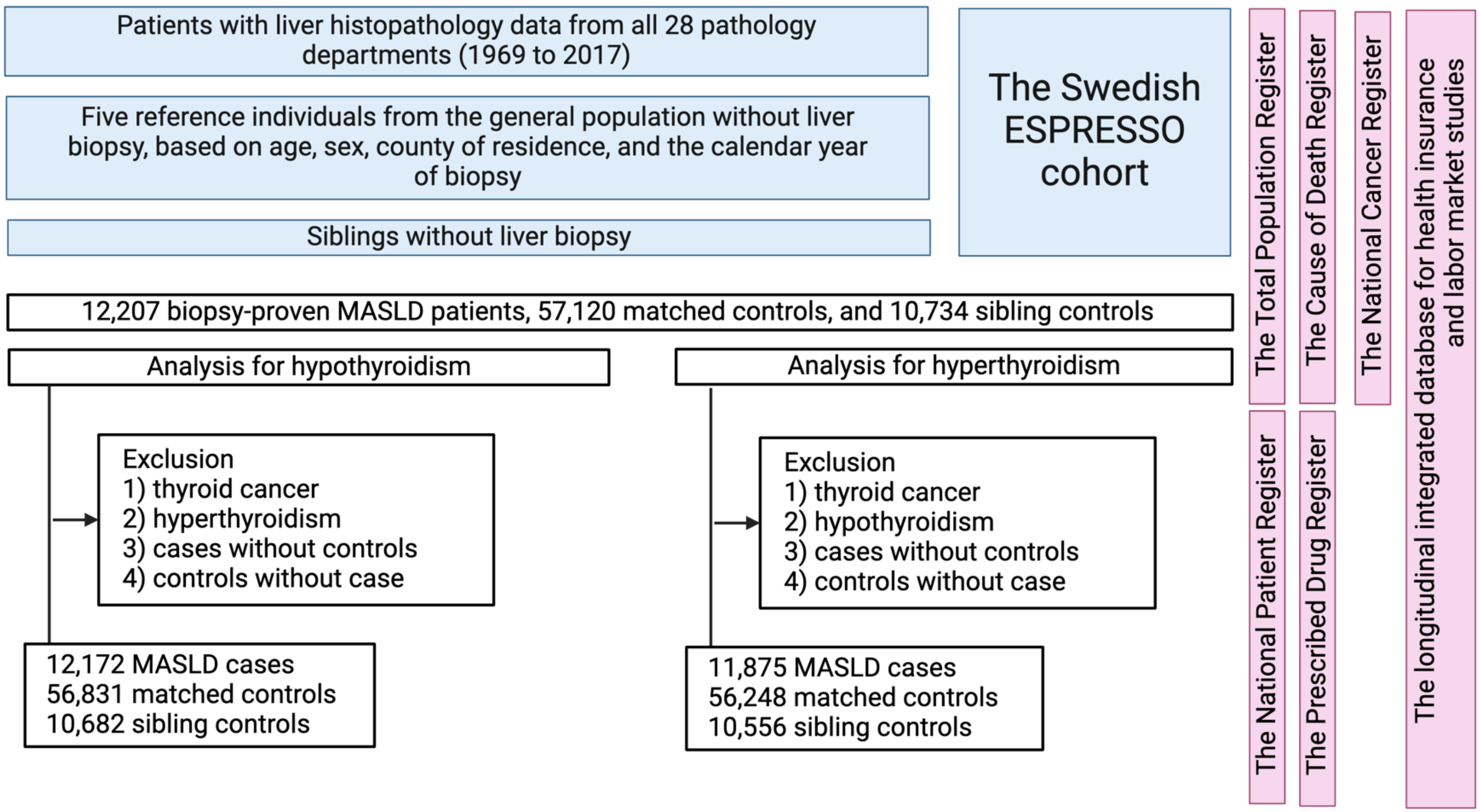

MASLD, metabolic dysfunction-associated steatotic liver disease.

Fig. S2. The directed acyclic graph distinguishing confounding and mediating roles of metabolic disorders in case-control study.

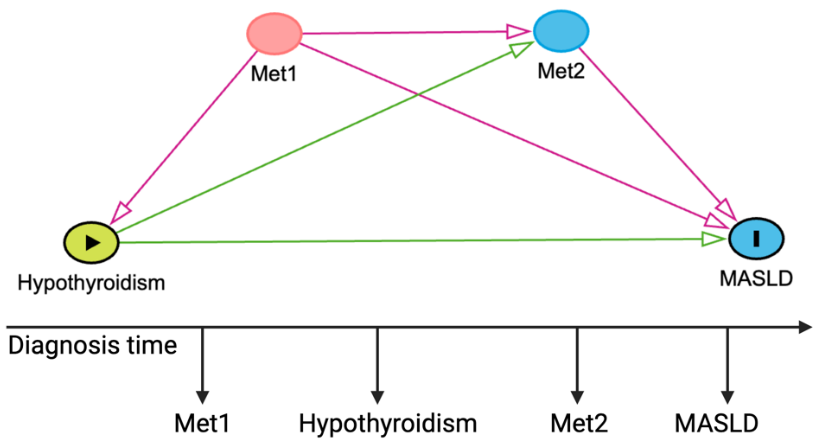

MASLD, metabolic dysfunction-associated steatotic liver disease. Met1 indicates metabolic disorders diagnosed before hypothyroidism and Met2 indicates metabolic disorders diagnosed after hypothyroidism and before MASLD.
