# Supplementary material for: Thyroid dysfunction in MASLD: Results of a nationwide study
Source: JHEP Rep. 2025 Feb 26;7(5):101369. doi: 10.1016/j.jhepr.2025.101369 (PMC12060450; doi:10.1016/j.jhepr.2025.101369)
Supplement: Multimedia component 4 [file mmc4.pdf]

JHEP|Reports

Thyroid dysfunction in MASLD: Results of a nationwide study

Authors

Shuai Yuan, Fahim Ebrahimi, David Bergman, …, Jie Chen, Hannes Hagström, Jonas F. Ludvigsson

Correspondence

shuai.yuan@ki.se (S. Yuan).

Graphical abstract

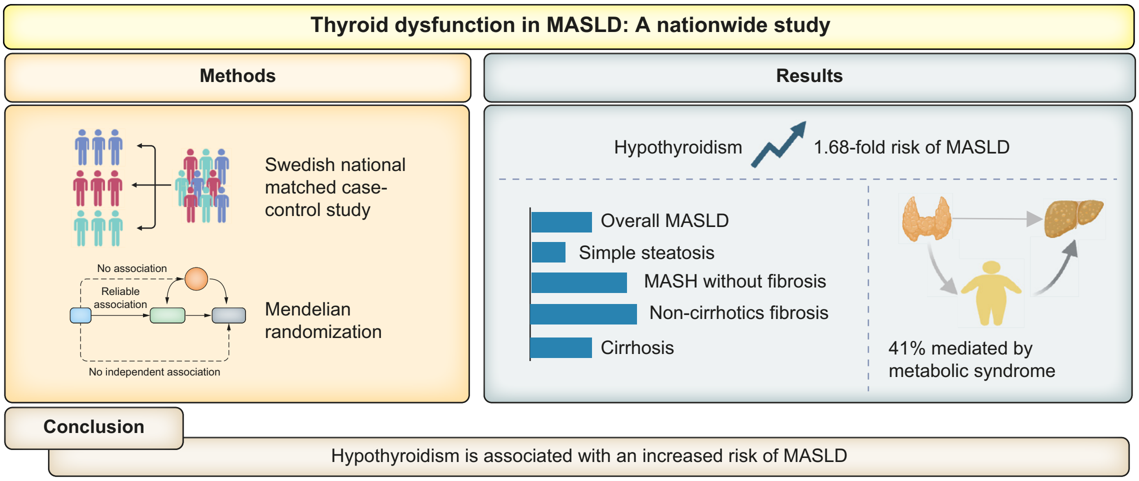

Highlights:

- Hypothyroidism raises MASLD odds by 1.68-fold, with stable sibling-based results.
- In terms of histological types, hypothyroidism–MASLD risk peaks in non-cirrhotic fibrosis.
- Metabolic disorders mediate ~41% of the hypothyroidism–MASLD association.
- Genetically predicted thyroid-stimulating hormone levels and hypothyroidism are linked to higher MASLD risk.
- Hyperthyroidism shows a potential inverse association with MASLD.

Impact and implications:

The approval by the US FDA of resmetirom, a thyroid hormone receptor β-selective agonist for non-cirrhotic metabolic dysfunction-associated steatohepatitis with stage 2–3 fibrosis, highlights the potential role of thyroid dysfunction in metabolic-associated steatotic liver disease (MASLD). This study identified hypothyroidism as a risk factor for MASLD, especially in men and individuals younger than 40 years, with the association peaking at non-cirrhotic fibrosis. Metabolic disorders mediated ~41% of the hypothyroidism–MASLD association. Hyperthyroidism was potentially inversely associated with MASLD. Despite its low prevalence (2.5% in MASLD cases, 1.4% in controls), the population health impact of hypothyroidism warrants further attention.

https://doi.org/10.1016/j.jhepr.2025.101369

© 2025 The Author(s). Published by Elsevier B.V. on behalf of European Association for the Study of the Liver (EASL). This is an open access article under the CC BY license (http://creativecommons.org/licenses/by/4.0/). JHEP Reports, 2025, 7, 1–8

Research article

JHEP|Reports

Thyroid dysfunction in MASLD: Results of a nationwide study

Shuai Yuan[1,2,3,*], Fahim Ebrahimi[4,5], David Bergman[4], Marijana Vujković[3,6], Eleonora Scorletti[7,8,9], Xixin Ruan[10], Jie Chen[10], Hannes Hagström[11,12], Jonas F. Ludvigsson[4,13,14]

JHEP Reports **2025**. vol. 7 | 1–8

Check for updates

Background & Aims: Thyroid hormones are known to be potent modulators of hepatic metabolism and targeting the thyroid hormone receptor was recently approved as the first treatment for metabolic-associated steatotic liver disease (MASLD); however, the exact relationship between thyroid disorders and biopsy-confirmed MASLD remains unclear.

Methods: We conducted a nationwide matched case–control study leveraging data from the Swedish Epidemiology Strengthened by histoPathology Reports in Sweden (ESPRESSO) cohort, which includes liver biopsy data spanning from 1969 to 2017. We identified 12,172 patients with MASLD and 56,831 matched general-population controls, including 5,478 patients with MASLD with 10,682 sibling controls. Conditional logistic regression was used to calculate odds ratios for hypothyroidism and hyperthyroidism defined through ICD codes or prescription records. Causal inference was examined using Mendelian randomization (MR). Both observational and MR mediation analyses were performed to explore the roles of metabolic features.

Results: Hypothyroidism was associated with 1.68-fold increased odds of MASLD (95% CI 1.36–2.06). The association remained stable in the analysis using siblings as controls. However, in absolute terms, hypothyroidism was uncommon and seen in 2.5% in people with MASLD and in 1.4% of controls. Higher genetically predicted thyroid-stimulating hormone levels and hypothyroidism were linked to increased MASLD risk. Mediation analysis showed that metabolic disorders contributed ~41% to this risk. Furthermore, there was an inverse association between hyperthyroidism and MASLD (adjusted odds ratio 0.17, 95% CI 0.05–0.56); however, the association did not reach statistical significance in the MR analysis.

Conclusions: The findings suggest that hypothyroidism is associated with a heightened risk of MASLD and that hyperthyroidism is potentially protective against MASLD.

© 2025 The Author(s). Published by Elsevier B.V. on behalf of European Association for the Study of the Liver (EASL). This is an open access article under the CC BY license (http://creativecommons.org/licenses/by/4.0/).

Introduction

Metabolic-associated steatotic liver disease (MASLD) has become a major global health issue, affecting ~38% of adults worldwide.[1] Over the past three decades, the prevalence of MASLD has risen sharply,[2] but treatment options remain limited despite this growing burden. However, in March 2024, the US FDA approved the first drug for MASLD, resmetirom, which is an oral, liver-directed, thyroid hormone receptor β-selective agonist.[3] This approval, specifically for patients with non-cirrhotic metabolic dysfunction-associated steatohepatitis (MASH) and stage 2–3 fibrosis, underscores the potential role of thyroid dysfunction in MASLD pathogenesis.

Impaired thyroid function is known to alter hepatic metabolism by impacting lipid metabolism, fatty acid oxidation, and energy production.[4] Epidemiological studies have also explored the relationship between hypothyroidism and MASLD, with meta-analyses generally indicating a positive association.[5,6] However, the causal role of hypothyroidism in MASLD development remains unclear as a result of several limitations: (1) significant heterogeneity of diagnostic modalities for MASLD and missing information on liver histology; (2) predominance of cross-sectional study designs; (3) small sample sizes; and (4) potential residual confounding resulting from inadequate adjustment for key variables, such as metabolic disorders and autoimmune diseases. Furthermore, the role of sex differences in this association has not been fully investigated.[7] To strengthen causal inference, Mendelian randomization (MR) has been proposed as a valuable approach, using genetic variants as instrumental variables to reduce confounding and reverse causation.[8] In terms of the association between hyperthyroidism and MASLD, few studies have been conducted,[9,10] and often only with a small sample size.

The increasing prevalence of MASLD is linked to metabolic risk factors.[11] Hypothyroidism, a common endocrine disorder characterized by insufficient thyroid hormone production, has been implicated in various metabolic disturbances, particularly weight gain and dyslipidemia, which could serve as pathways linking hypothyroidism to MASLD.[12–14] However, few studies have thoroughly investigated this potential mechanism.[15] Thus, we used a nationwide matched case–control and MR approach to assess the association between hypothyroidism, hyperthyroidism, and MASLD, and to explore the potential mediating roles of metabolic traits in the hypothyroidism–MASLD association.

* Corresponding author. Address: Institute of Environmental Medicine, Karolinska Institutet, Nobels väg 13, 171 65 Solna, Stockholm, Sweden.
E-mail address: shuai.yuan@ki.se (S. Yuan).
https://doi.org/10.1016/j.jhepr.2025.101369

Materials and methods

We conducted a nationwide matched case–control study to estimate the associations of a previous diagnosis of hypothyroidism and hyperthyroidism with biopsy-proven MASLD. To strengthen causality of the association, we performed a two-sample MR analysis using data from large-scale studies and biobanks. Mediation analysis was performed to understand the biological pathways.

Nationwide matched case–control study

Data source and study population

The case–control analysis was based on data from the Epidemiology Strengthened by histoPathology Reports in Sweden (ESPRESSO) cohort, which prospectively collected liver histopathology data from all 28 pathology departments across Sweden between 1969 and 2017.[16] Each histopathology report includes a unique personal identity number, biopsy date, liver topography, and morphological details. We identified all adult patients aged 18 years or older who underwent liver biopsy between 1969 and 2017. Among these, we first removed those who had emigrated from Sweden before the liver biopsy date (the index date) and then excluded patients with a history of alcohol abuse/misuse, other causes of acute or chronic liver disease, or previous liver transplantation, using a validated ICD algorithm (Table S1).[17] We then defined patients with MASLD according to Systematized Nomenclature of Medicine Clinical Terms (SNOMED) and ICD codes (Table S2). This approach previously demonstrated a positive predictive value (PPV) of 92% for identifying MASLD.[17] Patients with MASLD were further classified into four histological subgroups based on SNOMED definitions[18]: simple steatosis; non-alcoholic steatohepatitis (MASH) without fibrosis; MASLD with non-cirrhotic fibrosis; and cirrhosis. Individuals with liver biopsy data were matched with up to five reference individuals from the general population who had no record of earlier liver biopsy at time of matching, based on age, sex, county of residence, and the calendar year of biopsy. We also obtained data on individuals with liver biopsy data and their sibling controls without liver biopsy.

We further excluded patients with thyroid cancer and hyperthyroidism before MASLD diagnosis in the analysis of hypothyroidism, and patients with thyroid cancer and hypothyroidism before MASLD diagnosis in the analysis of hyperthyroidism (Fig. S1). Identical exclusion criteria were applied among the general population controls and sibling controls. We linked ESPRESSO to validated nationwide registers that contain data on demographics, comorbidities, prescribed medication, and mortality.

Ethical permit

The study was approved by the Stockholm Ethics Review Board (2014/1287-31/4, 2018/972-32, and 2022-05774-02), and informed consent was waived because of the register-based nature of the research.[19]

Definition of hypothyroidism and hyperthyroidism

Hypothyroidism was identified using ICD codes (ICD-8: 245,02, 245,03; ICD-9: 245C; ICD-10: E06.3) from the National Patient Register, which has recorded hospitalization data since 1964 and specialized outpatient visit data since 2001. Given that patients with hypothyroidism can be diagnosed and treated in primary care, we also used the prescription of levothyroxine (ATC H03AA01) and liothyronine (ATC H03AA02) as one of the definitions of hypothyroidism based on data from 1 July 2005 onward from the Swedish Prescribed Drug Register.[20] Likewise, hyperthyroidism was defined using ICD codes (ICD-8: 242,0–2; ICD-9: 242A–E, 376D; ICD-10: E05.0–E05.3, H06.2) in the National Patient Register.

Assessment of covariates and potential mediators

We gathered comprehensive information on demographics, comorbidities, and medication use of each individual (Table S3). In brief, age at the index date (biopsy date for patients with MASLD or the corresponding matching date for controls), sex, date of birth, and emigration status were obtained from the Total Population Register.[21] Education level was sourced from the Longitudinal Integrated Database for Health Insurance and Labour Market Studies (LISA),[22] which provides education data for >98% of all individuals aged 25–64 years, with an estimated accuracy for highest attained level of education of 85%.[22] Comorbidities, including pernicious anemia, metabolic disorders (diabetes, obesity, dyslipidemia, or hypertension), chronic obstructive pulmonary disease (COPD), and autoimmune diseases[23] (Table S4) were identified using established ICD algorithms from the National Patient Register with PPVs ranging from 85% to 95%.[24] COPD with diagnostic age ≥40 years was used as a proxy for heavy smoking. Medication data, including lipid-lowering drugs, antidiabetic medication, and antihypertensive medication, were retrieved from the Prescribed Drug Register.[20] Given the temporal sequence of the diagnosis of metabolic disorder, hypothyroidism, and MASLD among cases, metabolic disorders diagnosed before hypothyroidism were deemed a confounder, whereas metabolic disorders diagnosed after hypothyroidism and before MASLD were treated as potential mediators (Fig. S2).

Statistical analyses

We conducted conditional logistic regression to assess the relationship between hypothyroidism, hyperthyroidism, and MASLD. Crude and multivariable-adjusted odds ratios (ORs) with 95% CIs were estimated, accounting for matching factors, including age, sex, calendar year, and county of residence. I addition, adjustments were made for potential confounders, including education level (≤9 years, 10–12 years, ≥13 years, or missing), country of birth (Nordic or other), metabolic disorders diagnosed before hypothyroidism, COPD, pernicious anemia, autoimmune diseases, and the number of inpatient and outpatient visits over the past 12 months before the index/matching date (used as a proxy for healthcare utilization). Assuming the causality between hypothyroidism and MASLD, we used the ‘mediation’ R package[25] (R Foundation for Statistical Analysis, Vienna, Austria) to estimate the mediation of metabolic disorders diagnosed after hypothyroidism. The analysis is based on nonparametric bootstrapping to generate mediation effects, allowing for robust inference about the mediation pathways. We then performed the analysis among patients with MASLD by treating simple steatosis as the reference group. We conducted a sensitivity analysis by

changing the control population to full siblings of patients with MASLD to control for potential confounding factors related to genetic predisposition and shared childhood exposures. Subgroup analyses were performed stratified by sex (men and women) and age (<40 years and ≥40 years). For hyperthyroidism, we abstained from subgroup and mediation analyses because of inadequate statistical power. All analyses were performed using R version 4.4.1 (R Foundation for Statistical Computing, Vienna, Austria), with statistical significance defined as a two-sided p value of ≤0.05.

MR study

Study design

Hypothyroidism is characterized by low levels of thyroid hormones and increased levels of thyroid-stimulating hormone (TSH). Thus, we used TSH and clinically diagnosed hypothyroidism and hyperthyroidism as the exposures in the MR analyses. We first conducted a two-sample MR analysis to investigate the associations of genetic liability to higher levels of TSH, hypothyroidism, and hyperthyroidism with risk of MASLD. We further conducted multivariable MR analyses to examine whether metabolic traits mediated the associations. Given that MR analyses were based on publicly available summary data, an ethical permit was not required.

Genetic instrument selection

The TSH genome-wide association study (GWAS) data werebsourced from the ThyroidOmics Consortium (https://transfer.sysepi.medizin.uni-greifswald.de/thyroidomics/datasets/), where TSH levels were measured in 271,040 European participants whose levels fell within the upper and lower 2.5% of the TSH distribution, followed by inverse normal transformation.[26] The study accounted for assay and population characteristics, as well as environmental factors, such as population iodine supply.[26] GWAS data on hypothyroidism were extracted from the Veteran Authority's (VA) Million Veteran Program, where hypothyroidism was defined by Phecode 244 with diagnostic data from hospital electronic health records.[27] To minimize population structure bias, we obtained data on participants of European ancestry, including 62,814 cases and 378,321 non-cases of MASLD (https://www.ncbi.nlm.nih.gov/projects/gap/cgi-bin/study.cgi?study_id=phs002453.v1.p1). Summary GWAS data on hyperthyroidism were available in a meta-analysis of three biobanks including 3,271 cases and 512,267 non-cases of MASLD.[28] We first obtained all genetic variants (*i.e.* single nucleotide polymorphisms [SNPs]) associated with the exposure at the genome-wide significance level ($p < 5 \times 10^{-8}$) and then clumped these SNPs by linkage disequilibrium ($r^2 < 0.001$). In total, 254, 265, and 24 SNPs were used as genetic instruments for TSH, hypothyroidism, and hyperthyroidism, respectively. All retained SNPs had F statistic >10, indicating limited weak instrument bias.[29] Detailed information on used SNPs is available in Table S5.

Data sources for MASLD

Summary-level data on MASLD were obtained from a GWAS meta-analysis of UK Biobank, Estonian Biobank, eMERGE, and FinnGen, including 8,434 MASLD cases, all identified through electronic health records, and 770,180 non-cases of MASLD.[30] Detailed diagnostic codes can be found in the original GWAS.[30] Associations were adjusted for age, sex, and top genetic principal components.

Data sources for metabolic traits

Based on previous findings,[31] obesity, type 2 diabetes mellitus (T2DM), triglycerides and systolic blood pressure were selected as MASLD-associated metabolic traits and included in the multivariable MR analysis. Summary-level data on BMI,[32] T2DM (www.ncbi.nlm.nih.gov/projects/gap/cgi-bin/study.cgi?study_id=phs002453.v1.p1),[33] triglycerides (www.nealelab.is/uk-biobank), and systolic blood pressure[34] were available in GWASs including 806,834, 1,407,282 (228,499 cases), 343,992, and 1,006,863 individuals of European ancestry, respectively.

Statistical analyses

Exposure and outcome data were harmonized based on effect and non-effect alleles. SNPs with allele mismatch were removed. We used the inverse variance weighted (IVW) method with multiplicative random effects as the primary analysis, supplemented by three sensitivity analyses: the weighted median method,[35] MR-Egger regression,[36] and MR-PRESSO.[37] The IVW method is known for its precision but is highly sensitive to SNP outliers, which can lead to horizontal pleiotropy. By contrast, the weighted median method can provide a robust estimate when more than half of the total weight comes from valid SNPs. The MR-Egger regression includes an intercept test to assess horizontal pleiotropy and offers corrected estimates, although it tends to be underpowered. It also detects outlier SNPs and recalculates estimates after their removal, improving the robustness of the results. We calculated Cochran's Q to evaluate heterogeneity among SNP estimates. Horizontal pleiotropy was assessed using the MR-Egger intercept test and the MR-PRESSO global test. Multivariable MR using exposure-specific instruments was performed using the MendelianRandomization R package and mediation of metabolic traits was calculated using the formula: (total effect-direct effect)/total effect. Two-sample MR analysis was conducted using the TwoSampleMR package in R 4.3.2. Statistical significance was set at a two-sided p value of ≤0.05.

Results

Nationwide matched case–control study

The characteristics of the cases of MASLD and general population controls included in the study are shown in Table 1. In the analysis of hypothyroidism, we included a total of 12,172 MASLD cases with different histological features and 56,831 matched population comparators. Patients with MASLD were more frequently diagnosed with diabetes mellitus, obesity, dyslipidemia, and hypertension compared with controls. For the analysis treating siblings as controls, we included 5,478 MASLD cases with 10,682 sibling comparators.

A previous diagnosis of hypothyroidism was observed in 309 (2.5%) patients with MASLD and 819 (1.4%) controls, indicating a 1.81-fold increased odds of MASLD (95% CI 1.57–2.09) in the crude model. This association persisted in the

Table 1. Characteristics of MASLD cases and controls.

Characteristic	Population controls n = 56,831	All MASLD n = 12,172	Simple steatosis n = 8,144	MASH without fibrosis n = 1,399	Non-cirrhotic fibrosis n = 1,941	Cirrhosis n = 688
Sex, n (%)						
Men	30,795 (54.2)	6,666 (54.8)	4,517 (55.5)	721 (51.5)	1,052 (54.2)	376 (54.7)
Women	26,036 (45.8)	5,506 (45.2)	3,627 (44.5)	678 (48.5)	889 (45.8)	312 (45.3)
Age						
Mean (SD)	52.1 (16.4)	52.4 (16.4)	51.9 (16.0)	52.1 (17.1)	52.4 (17.9)	58.5 (13.7)
Median (IQR)	54 (41–64)	54 (42–64)	53 (41–64)	54 (41–64)	56 (43–65)	61 (52–67)
Country of birth, n (%)						
Nordic country	52,097 (91.7)	10,946 (89.9)	7,376 (90.6)	1,241 (88.7)	1,705 (87.8)	624 (90.7)
Other	4,734 (8.3)	1,226 (10.1)	768 (9.4)	158 (11.3)	236 (12.2)	64 (9.3)
Education level*, n (%)						
≤9 years	18,027 (31.7)	3,959 (32.5)	2,643 (32.5)	443 (31.7)	606 (31.2)	267 (38.8)
10–12 years	22,016 (38.7)	4,963 (40.8)	3,266 (40.1)	596 (42.6)	829 (42.7)	272 (39.5)
≥13 years	14,234 (25.1)	2,466 (20.3)	1,681 (20.6)	278 (19.9)	417 (21.5)	90 (13.1)
Missing	2,554 (4.5)	784 (6.4)	554 (6.8)	82 (5.8)	89 (4.6)	59 (8.6)
Start of follow-up, year						
1969–1989	11,016 (19.4)	2,293 (18.9)	1,815 (26.2)	182 (13.0)	159 (8.2)	137 (20.0)
1990–2005	31,694 (55.8)	6,751 (55.5)	4,723 (58.1)	787 (56.3)	893 (46.0)	348 (50.7)
2006–2019	14,063 (24.8)	3,116 (25.6)	1,598 (19.6)	429 (30.7)	888 (45.8)	201 (29.3)
**Comorbidities, n (%)**						
Any metabolic comorbidity	7,092 (12.5)	2,975 (24.4)	1,654 (20.3)	379 (27.1)	704 (36.3)	238 (34.6)
Diabetes	1,798 (3.2)	1,137 (9.3)	585 (7.2)	134 (9.6)	271 (14.0)	147 (21.4)
Obesity	111 (0.2)	218 (1.8)	121 (1.5)	39 (2.8)	51 (2.6)	7 (1.0)
Dyslipidemia	3,994 (7.0)	1,402 (11.5)	786 (9.7)	191 (13.7)	336 (17.3)	89 (12.9)
Hypertension	3,361 (5.9)	1,306 (10.7)	659 (8.1)	175 (12.5)	349 (18.0)	123 (17.9)
No. of hospital visits over past 12 months (mean, SD)	2.6 (8.1)	8.6 (17.8)	6.9 (14.5)	9.8 (18.9)	14.6 (26.0)	8.9 (17.8)

All variables reported as mean (SD) or n (%), unless described otherwise.
MASH, metabolic dysfunction-associated steatohepatitis.
*Education categories were based on compulsory school, high school, and college. Education level was recorded beginning in 1990; thus, data presented are for persons with index dates on or after 1 January, 1990. Persons with index dates before 1990 had their education level recorded as missing.

multivariable analysis (OR 1.68; 95% CI 1.36–2.06). In addition, the relationship between previous hypothyroidism and the development of MASLD varied across histological stages. The OR increased from 1.22 (95% CI 0.91–1.65) for simple steatosis to 2.54 (95% CI 1.44–4.48) for MASH without fibrosis, and further to 2.58 (95% CI 1.76–3.78) for MASLD with non-cirrhotic fibrosis (Table 2). However, for cirrhosis, the OR was 1.46 (95% CI 0.54–3.96). This pattern was also observed in the analysis among MASLD by treating simple steatosis as the reference group (Table S6).

Subgroup analysis revealed possibly higher risks of MASLD following a previous hypothyroidism diagnosis in men and in the group younger than 40 years (Table S7). The OR of MASLD was 3.10 (95% CI 2.02–4.76) in men and 5.32 (95% CI 2.29–12.34) in the group younger than 40 years. The association between hypothyroidism and MASLD remained stable in the sensitivity analysis treating unaffected siblings as controls (Table S8). The mediation analysis revealed that 41.2% (95% 30.1–53.0%) of the hypothyroidism–MASLD association was mediated by metabolic disorders diagnosed after hypothyroidism and before MASLD.

In the analysis of hyperthyroidism, we included a total of 11,875 MASLD cases and 56,248 matched population comparators. A previous diagnosis of hyperthyroidism was observed in 11 (0.09%) patients with MASLD and 69 (0.13%) controls. Compared with non-hyperthyroidism, prior diagnosis of hyperthyroidism was associated with a reduced odds of MASLD in the crude model (OR 0.74, 95% CI 0.39–1.40) and (OR 0.17, 95% CI 0.05–0.56) in the multivariable analysis. The association became clear after adjusting for autoimmune disease and remained stable in the analysis treating unaffected siblings as controls.

Table 2. Association between hypothyroidism and MASLD among MASLD cases and general population controls.

	Population controls n = 56,831	All MASLD n = 12,172	Simple steatosis n = 8,144	MASH without fibrosis n = 1,399	Non-cirrhotic fibrosis n = 1,941	Cirrhosis n = 688
%	1.4	2.5	1.6	3.7	5.3	2.8
Crude model, OR (95% CI)	1 (ref.)	1.81 (1.57–2.09)	1.55 (1.26–1.92)	2.11 (1.47–3.04)	2.32 (1.79–3.00)	1.29 (0.75–2.21)
Multivariable-adjusted model, OR (95% CI)	1 (ref.)	1.68 (1.36–2.06)	1.22 (0.91–1.65)	2.54 (1.44–4.48)	2.58 (1.76–3.78)	1.46 (0.54–3.96)

Estimates were obtained from conditional logistic regression; all models were conditioned on stratum factors (age, sex, biopsy calendar year, and county). Multivariable-adjusted model was adjusted for education, country at birth, metabolic disorders (obesity, type 2 diabetes mellitus, dyslipidemia, and hypertension), autoimmune disease, chronic obstructive pulmonary disease, anemia, and clinical visit in the past 12 months.
MASH, metabolic dysfunction-associated steatohepatitis; OR, odds ratio.

MR study

Out of the 254 SNPs associated with TSH, 226 were available in the MASLD GWAS dataset, whereas 219 of the 265 SNPs associated with hypothyroidism were present in the same dataset. Genetically predicted higher levels of TSH and genetic liability to hypothyroidism were associated with an increased risk of MASLD (Table 3). The OR of MASLD was 1.10 (95% CI 1.03–1.17) per SD increase in genetically predicted inverse normal transformed TSH levels, and 1.07 (95% CI 1.03–1.12) per unit increase in genetic predisposition to hypothyroidism. Genetic liability to hyperthyroidism was not associated with MASLD (OR 0.92, 95% CI 0.84–1.01; p = 0.096). The associations remained generally consistent in sensitivity analyses and no significant heterogeneity or horizontal pleiotropy was detected (Table 3).

Approximately 5.4% and 19.4% of the associations between genetically predicted TSH and MASLD was mediated by BMI and triglycerides, respectively. Likewise, 2.8% and 9.7% of the associations between genetically predicted hypothyroidism and MASLD was mediated by BMI and triglycerides, respectively. However, these analyses were underpowered to detect statistically significant mediation effects (Table S9). Limited mediation was observed for genetically predicted T2DM or systolic blood pressure.

Discussion

This nationwide case–control and MR study identified a positive association between previous hypothyroidism and an elevated risk of developing MASLD. This association was pronounced in men and individuals younger than 40 years. The association intensified in more advanced stages of MASLD, peaking with non-cirrhotic fibrosis, but did not continue to increase as the disease progressed to cirrhosis. Metabolic disorders that developed after diagnosis of hypothyroidism mediated ∼41% of this association. We also found an inverse association between a previous diagnosis of hyperthyroidism and MASLD development; however, this association was not observed in the MR analysis. In absolute terms, hypothyroidism was uncommon and seen in 2.5% in people living with MASLD and in 1.4% controls. Although hypothyroidism might have a lower prevalence, the condition is common enough to have a meaningful population health impact.

Our finding of a positive association between hypothyroidism and MASLD is consistent with previous research.[5,6] A meta-analysis of 13 studies (two cohorts, four case–control, and seven cross-sectional with a sample size up to 18,544) reported a significant association between hypothyroidism and MASLD (OR, 1.52, 95% CI 1.24–1.87), despite considerable heterogeneity among the studies.[5] Another meta-analysis, which included 12 cross-sectional and three longitudinal studies, also found a similar association (OR, 1.42, 95% CI 1.15–1.77).[6] A positive association was also observed between higher levels of TSH and higher levels of liver fibrosis.[38] Furthermore, an MR study utilizing GWAS data from 1,483 European non-alcoholic fatty liver disease cases and 17,781 controls provided additional evidence supporting this link.[39] Our study, which leveraged a nationwide dataset and was well powered, further reinforces the positive association between hypothyroidism and MASLD, adding robust support to the existing body of evidence.

The meta-analysis of three studies, although limited by small sample sizes, investigated the link between hypothyroidism and the risk of severe MASLD,[6] finding that the magnitude of risk closely mirrored the severity of MASLD histology.[6] This partially aligns with our results in that we observed a similar trend, but did not observe a continued increase in risk for cirrhosis. Interestingly, another study reported that, although hypothyroidism was highly prevalent among patients with MASLD and was associated with increased MASLD inflammatory activity, it was not linked to fibrosis or grade of steatosis.[40] A potential explanation for this is that, given that the development of cirrhosis is a prolonged process, hypothyroidism diagnosed in the 5–10 years before cirrhosis diagnosis might have only a marginal impact on liver disease progression. In addition, this might reflect that hypothyroidism might not be a universal driver of MASLD, but rather a factor in a specific subgroup of patients who have a unique hepatic or systemic

Table 3. Genetically predicted higher levels of TSH and hypothyroidism in relation to risk of MASLD in MR analysis.

Exposure	Method	OR (95% CI)	p value
Genetically predicted TSH levels	Inverse variance weighted with multiplicative random effects	1.10 (1.03–1.17)	0.004
	Weighted median	1.11 (1.00–1.24)	0.049
	MR-Egger	1.02 (0.91–1.15)	0.735
	Cochran's Q = 224; $P_{\text{MR-Egger intercept test}}$ = 0.159; $P_{\text{MR-PRESSO global test}}$ = 0.410		
Genetic liability to hypothyroidism	Inverse variance weighted with multiplicative random effects	1.07 (1.03– 1.12)	0.001
	Weighted median	1.07 (1.00–1.15)	0.045
	MR-Egger	1.05 (0.96–1.15)	0.282
	Cochran's Q = 206; $P_{\text{MR-Egger intercept test}}$ = 0.513; $P_{\text{MR-PRESSO global test}}$ = 0.617		
Genetic liability to hyperthyroidism	Inverse variance weighted with multiplicative random effects	0.92 (0.84–1.01)	0.096
	Weighted median	0.94 (0.79–1.12)	0.499
	MR-Egger	0.91 (0.67–1.25)	0.567
	Cochran's Q = 14; $P_{\text{MR-Egger intercept test}}$ = 0.931; $P_{\text{MR-PRESSO global test}}$ = NA		

Results were obtained using method listed in the 'Method' column and are given as OR with 95% CI for genetic liability to MASLD as outcome; p <0.05 were considered statistically significant. MR-PRESSO detected no outlier in either analysis. MASH, metabolic dysfunction-associated steatohepatitis; MR, Mendelian randomization; OR, odds ratio; TSH, thyroid-stimulating hormone.

metabolic response. This hypothesis is partly supported by the results of the trial of resmetirom, where only ~30% of the high-dose patients responded.[3] However, we cannot rule out that the null association for grade of steatosis might be caused by underdiagnosis of subclinical hypothyroidism or inadequate power because of the inclusion of fewer patients with cirrhosis. Nonetheless, these findings collectively underscore the potential importance of early detection and management of hypothyroidism in mitigating the progression of MASLD.

In our study, we observed a notably stronger association between hypothyroidism and MASLD in men (OR, 3.10), which aligns with findings from a Korean cross-sectional study.[7] In addition, we identified a more pronounced association in individuals younger than 40 years (OR, 5.32) compared with those older than 40 years (OR, 1.49). These findings suggest that younger individuals and men are at higher risk for MASLD in the context of hypothyroidism. MASLD is most commonly the consequence of long-standing obesity. Thus, these data underscore the fact that an alternative underlying mechanism leading to the development of MASLD in younger individuals might be hypothyroidism. However, future studies examining all patients in such subgroups are needed.

Although the role of metabolic disorders as intermediaries between hypothyroidism and MASLD has been suggested,[13–15] empirical evidence remains limited. Our cohort study found that ~41% of the association between hypothyroidism and MASLD might be mediated by metabolic disorders. Notably, multivariable MR analysis identified triglycerides as a key mediator, consistent with findings from animal studies.[12] These are consistent with the understanding that metabolic disorder is a key metabolic risk factor for MASLD. Although a larger proportion of the hypothyroidism–MASLD association was mediated by metabolic disorders, hypothyroidism still retained a direct, unmediated effect on MASLD risk. These findings emphasize the dual role of hypothyroidism as: (1) an upstream factor that contributes to metabolic disorders; and (2) an independent risk factor for MASLD through mechanisms unrelated to traditional metabolic disorders, such as direct effects on liver metabolism, inflammation, or fibrosis pathways, implying that liver cells metabolize intracellular fat without noticeable changes in systemic metabolism. Thus, further research is necessary to investigate other alternative pathways and enhance our understanding of the relationship between hypothyroidism and MASLD.

We also found a lower risk of MASLD in patients with hyperthyroidism. This is consistent with earlier studies[9,10,41] and in line with recent trial data demonstrating a beneficial effect of resmetirom.[3]

Our paper has some strengths. As a nationwide case–control study, >12,000 individuals with biopsy-verified MASLD were included. Excluding other liver diseases, our definition of MASLD had a high PPV.[17] We adjusted our analyses for a large number of covariates, including healthcare consumption, and confirmed our hypothyroidism results in a sibling comparison that should have also minimized intrafamilial confounding. Not all patients with hyperthyroidism and hypothyroidism are detected through the National Patient Registry and, therefore, we also exploited Prescribed Drug Register data to increase our sensitivity for thyroid disease. Finally, we carried out MR analyses to triangulate our findings. Notably, directly comparing the magnitude of observational and MR associations was challenging because of differences in exposure measurement, that is, clinical diagnosis in the case–control study versus genetic predisposition in MR. However, these two approaches complement each other: the case–control study reflects real-world clinical implications by using actual disease diagnoses, whereas MR strengthens causal inference by mitigating confounding and reverse causation through the use of genetic instruments.

We acknowledge several limitations in our study. First, the analysis was limited to individuals who underwent liver biopsy, which inherently introduces selection bias. Liver biopsy is typically performed in patients with more severe or atypical presentations of disease, making this cohort less representative of the broader MASLD population. This selection bias is evidenced by the lower rate of simple steatosis observed in our study (67%) compared with the expected rate of 75–80% in the general population. However, the persistence of associations between thyroid diseases and MASLD in this cohort suggests that these links are not confined to early stages of the disease, but also extend to more advanced stages. This highlights the potential role of thyroid diseases as clinically significant risk factors across the MASLD spectrum. By contrast, the observed discrepancy might also partly reflect potential misclassification or variability in pathologist coding practices, particularly given the absence of centralized biopsy reading. Nevertheless, the previous validation of pathology reports provides confidence that steatosis was accurately identified, mitigating concerns about misclassification.[17] Second, we lacked detailed data on the specific indications for liver biopsy. Although liver biopsy in Sweden is generally performed in patients with clinical suspicion of advanced fibrosis or alternative diagnoses, the absence of precise indication data limited our ability to fully contextualize the findings. Third, the general population comparator group did not undergo liver biopsy, preventing direct histological comparisons. This approach assumed that these individuals did not have clinically significant liver disease; however, the possibility of undetected MASLD or other liver conditions in this group cannot be entirely excluded, which could introduce potential bias. Nonetheless, given the invasive nature and associated risks of liver biopsy, it would be both clinically and scientifically inappropriate to compare patients with MASLD to individuals with mild findings who underwent biopsy that turned out to be ‘normal’. In addition, any misclassification of MASLD within the comparator group would likely bias the results toward the null, thereby underestimating the true association. This conservative bias strengthens confidence in the observed findings. Fourth, in the ESPRESSO cohort, we lacked data on thyroid-related hormones, liver function tests, or related biomarkers, which limits our ability to explore mechanistic pathways underlying the observed associations. Fifth, the analyses of hyperthyroidism and the subgroup analysis for cirrhosis were limited by low statistical power, potentially affecting the robustness of these findings. Sixth, while we identified associations between hypothyroidism, hyperthyroidism, and MASLD, we did not have data on the impact of hypothyroidism treatments on liver disease severity. Future studies incorporating treatment data and additional biomarkers could provide more comprehensive insights into these relationships. Finally, the absence of GWAS data on MASLD histopathological subtypes limited our ability to explore the associations between genetically predicted thyroid-related indicators and MASLD severity using MR.

In summary, our study found a significant association between hypothyroidism and an increased risk of developing MASLD, with a particularly pronounced risk in younger individuals and men. This risk escalated with MASLD severity, peaking at non-cirrhotic fibrosis, underscoring the importance of early intervention to mitigate disease progression. Finally, we found a potential inverse relationship between hyperthyroidism and MASLD, supporting an effect of newly established thyroid hormone receptor β-agonist treatment in MASLD.

Affiliations

[1]*Unit of Cardiovascular and Nutritional Epidemiology, Institute of Environmental Medicine, Karolinska Institutet, Stockholm, Sweden;* [2]*Department of Surgery, Perelman School of Medicine at the University of Pennsylvania, PA, USA;* [3]*Corporal Michael J. Crescenz VA Medical Center, Philadelphia, PA, USA;* [4]*Department of Medical Epidemiology and Biostatistics, Karolinska Institutet, Stockholm, Sweden;* [5]*Department of Gastroenterology and Hepatology, Clarunis University Center for Gastrointestinal and Liver Diseases, Basel, Switzerland;* [6]*Department of Medicine, University of Pennsylvania Perelman School of Medicine, Philadelphia, PA, USA;* [7]*School of Human Development and Health, Faculty of Medicine, University of Southampton, Southampton, UK;* [8]*National Institute for Health Research Southampton Biomedical Research Centre, University of Southampton and University Hospital Southampton National Health Service Foundation Trust, Southampton, UK;* [9]*Department of Genetics, Perelman School of Medicine, University of Pennsylvania, Philadelphia, PA, USA;* [10]*Department of Gastroenterology, Central South University Third Xiangya Hospital, Changsha, Hunan, China;* [11]*Division of Hepatology, Department of Upper GI, Karolinska University Hospital, Stockholm, Sweden;* [12]*Department of Medicine, Karolinska Institutet, Stockholm, Sweden;* [13]*Department of Pediatrics, Örebro University Hospital, Örebro, Sweden;* [14]*Division of Digestive and Liver Disease, Department of Medicine, Columbia University Medical Center, New York, NY, USA*

Abbreviations

COPD, chronic obstructive pulmonary disease; ESPRESSO, Epidemiology Strengthened by histoPathology Reports in Sweden cohort; GWAS, genome-wide association study; ICD, International Classification of Diseases; IVW, inverse variance weighted; LISA, Longitudinal Integrated Database for Health Insurance and Labour Market Studies; MASH, non-alcoholic steatohepatitis; MASLD, metabolic-associated steatotic liver disease; MR, Mendelian randomization; OR, odds ratio; PPV, positive predictive value; SNOMED, Systematized Nomenclature of Medicine Clinical Terms; SNPs, single nucleotide polymorphisms; T2DM, type 2 diabetes mellitus; TSH, thyroid-stimulating hormone; VA, Veteran Authority.

Financial support

SY is supported by an American Heart Association Postdoctoral Fellowship (24POST1189614).

Conflicts of interest

FE has served as an advisory board member for Boehringer Ingelheim. HH's institutions have received research funding from Astra Zeneca, EchoSens, Gilead, Intercept, MSD, Novo Nordisk, and Pfizer. HH has served as consultant or on advisory boards for Astra Zeneca, Bristol Myers-Squibb, MSD, and Novo Nordisk, and has been part of hepatic events adjudication committees for Arrowhead, Boehringer Ingelheim, KOWA and GW Pharma. JFL has coordinated an unrelated study on behalf of the Swedish Inflammatory Bowel Disease (IBD) quality register (SWIBREG). This study received funding from Janssen. He has also received financial support from MSD to develop a paper reviewing national healthcare registers in China and has funding from Takeda for a celiac disease project. JFL also has ongoing collaborations with MSD focusing on IBD. The other authors have no conflicts of interest to disclose.

Please refer to the accompanying ICMJE disclosure forms for further details.

Authors' contributions

Conceived and designed the study; authored the initial draft of the manuscript: SY, JFL.
Contributed to data collection: SY, XR, JFL.
Undertook the statistical analyses: SY.
Contributed to data interpretation, offered significant intellectual insights to the manuscript, and approved its final version: SY, FE, DB, MV, ES, XR, JC, HH, JFL.

Data availability statement

Data are not available because of Swedish data protection regulations.

Supplementary data

Supplementary data to this article can be found online at https://doi.org/10.1016/j.jhepr.2025.101369.

References

[1] Riazi K, Azhari H, Charette JH, et al. The prevalence and incidence of NAFLD worldwide: a systematic review and meta-analysis. Lancet Gastroenterol Hepatol 2022;7:851–861.
[2] Paik JM, Henry L, Younossi Y, et al. The burden of nonalcoholic fatty liver disease (NAFLD) is rapidly growing in every region of the world from 1990 to 2019. Hepatol Commun 2023;7(10):e0251.
[3] Harrison SA, Bedossa P, Guy CD, et al. A Phase 3, randomized, controlled trial of resmetirom in NASH with liver fibrosis. N Engl J Med 2024;390(6):497–509.
[4] Sinha RA, Bruinstroop E, Singh BK, et al. Nonalcoholic fatty liver disease and hypercholesterolemia: roles of thyroid hormones, metabolites, and agonists. Thyroid 2019;29(9):1173–1191.
[5] He W, An X, Li L, et al. Relationship between hypothyroidism and non-alcoholic fatty liver disease: a systematic review and meta-analysis. Front Endocrinol 2017;8:335.
[6] Mantovani A, Nascimbeni F, Lonardo A, et al. Association between primary hypothyroidism and nonalcoholic fatty liver disease: a systematic review and meta-analysis. Thyroid 2018;28(10):1270–1284.
[7] Lee J, Ha J, Jo K, et al. Male-specific association between subclinical hypothyroidism and the risk of non-alcoholic fatty liver disease estimated by hepatic steatosis index: korea National Health and Nutrition Examination Survey 2013 to 2015. Sci Rep 2018;8(1):15145.
[8] Sanderson E, Glymour MM, Holmes MV, et al. Mendelian randomization. Nat Rev Methods Primers 2022;2:6.
[9] Labenz C, Kostev K, Armandi A, et al. Impact of thyroid disorders on the incidence of non-alcoholic fatty liver disease in Germany. United Eur Gastroenterol J 2021;9(7):829–836.
[10] Bano A, Chaker L, Plompen EPC, et al. Thyroid function and the risk of nonalcoholic fatty liver disease: the Rotterdam Study. J Clin Endocrinol Metab 2016;101(8):3204–3211.
[11] European Association for the Study of the Liver (EASL). European association for the study of diabetes (EASD), European association for the study of obesity (EASO). EASL-EASD-EASO clinical practice guidelines on the management of metabolic dysfunction-associated steatotic liver disease (MASLD). J Hepatol 2024;81:492–542.
[12] Ferrandino G, Kaspari RR, Spadaro O, et al. Pathogenesis of hypothyroidism-induced NAFLD is driven by intra- and extrahepatic mechanisms. Proc Natl Acad Sci U S A. 2017;114(43):E9172–E9180.
[13] Mavromati M, Jornayvaz FR. Hypothyroidism-associated dyslipidemia: potential molecular mechanisms leading to NAFLD. Int J Mol Sci 2021;22(23):12797.
[14] Tanase DM, Gosav EM, Neculae E, et al. Hypothyroidism-induced nonalcoholic fatty liver disease (HIN): mechanisms and emerging therapeutic options. Int J Mol Sci 2020;21(16):5927.
[15] Hatziagelaki E, Paschou SA, Schön M, et al. NAFLD and thyroid function: pathophysiological and therapeutic considerations. Trends Endocrinol Metab 2022;33(11):755–768.
[16] Ludvigsson JF, Lashkariani M. Cohort profile: ESPRESSO (Epidemiology strengthened by histoPathology reports in Sweden). Clin Epidemiol 2019;11:101–114.
[17] Simon TG, Roelstraete B, Khalili H, et al. Mortality in biopsy-confirmed nonalcoholic fatty liver disease: results from a nationwide cohort. Gut 2021;70(7):1375–1382.
[18] Grabau D. KVAST dokument brösttumörer. Stockholm: Svensk Förening för Patologi – Svensk Förening för Klinisk Cytologi; 2014.
[19] Ludvigsson JF, Håberg SE, Knudsen GP, et al. Ethical aspects of registry-based research in the Nordic countries. Clin Epidemiol 2015;7:491–508.

[20] Wettermark B, Hammar N, Fored CM, et al. The new Swedish Prescribed Drug Register–opportunities for pharmacoepidemiological research and experience from the first six months. Pharmacoepidemiol Drug Saf 2007;16(7):726–735.
[21] Ludvigsson JF, Almqvist C, Bonamy AKE, et al. Registers of the Swedish total population and their use in medical research. Eur J Epidemiol 2016;31(2):125–136.
[22] Ludvigsson JF, Svedberg P, Olén O, et al. The longitudinal integrated database for health insurance and labour market studies (LISA) and its use in medical research. Eur J Epidemiol 2019;34(4):423–437.
[23] Yuan S, Leffler D, Lebwohl B, et al. Older age of celiac disease diagnosis and risk of autoimmune disease: a nationwide matched case-control study. J Autoimmun 2024;143:103170.
[24] Ludvigsson JF, Andersson E, Ekbom A, et al. External review and validation of the Swedish national inpatient register. BMC Public Health 2011;11:450.
[25] Tingley D, Yamamoto T, Hirose K, et al. Mediation: R package for causal mediation analysis. Los Angeles: UCLA Statistics/American Statistical Association; 2014.
[26] Sterenborg RBTM, Steinbrenner I, Li Y, et al. Multi-trait analysis characterizes the genetics of thyroid function and identifies causal associations with clinical implications. Nat Commun 2024;15(1):1–18.
[27] Verma A, Huffman JE, Rodriguez A, et al. Diversity and scale: genetic architecture of 2068 traits in the VA Million Veteran Program. Science 2024;385(6706):eadj1182.
[28] Sakaue S, Kanai M, Tanigawa Y, et al. A cross-population atlas of genetic associations for 220 human phenotypes. Nat Genet 2021;53(10):1415–1424.
[29] Burgess S, Davies NM, Thompson SG. Bias due to participant overlap in two-sample Mendelian randomization. Genet Epidemiol 2016;40(7):597–608.
[30] Ghodsian N, Abner E, Emdin CA, et al. Electronic health record-based genome-wide meta-analysis provides insights on the genetic architecture of non-alcoholic fatty liver disease. Cell Rep Med 2021;2(11):100437.
[31] Yuan S, Chen J, Li X, et al. Lifestyle and metabolic factors for nonalcoholic fatty liver disease: Mendelian randomization study. Eur J Epidemiol 2022;37(7):723–733.
[32] Pulit SL, Stoneman C, Morris AP, et al. Meta-analysis of genome-wide association studies for body fat distribution in 694 649 individuals of European ancestry. Hum Mol Genet 2019;28(1):166–174.
[33] Vujkovic M, Keaton JM, Lynch JA, et al. Discovery of 318 new risk loci for type 2 diabetes and related vascular outcomes among 1.4 million participants in a multi-ancestry meta-analysis. Nat Genet 2020;52(7):680–691.
[34] Evangelou E, Warren HR, Mosen-Ansorena D, et al. Genetic analysis of over 1 million people identifies 535 new loci associated with blood pressure traits. Nat Genet 2018;50(10):1412–1425.
[35] Bowden J, Davey Smith G, Haycock PC, et al. Consistent estimation in Mendelian randomization with some invalid instruments using a weighted median estimator. Genet Epidemiol 2016;40(4):304–314.
[36] Bowden J, Del Greco MF, Minelli C, et al. Assessing the suitability of summary data for two-sample Mendelian randomization analyses using MR-Egger regression: the role of the I2 statistic. Int J Epidemiol 2016;45(6):1961–1974.
[37] Verbanck M, Chen CY, Neale B, et al. Detection of widespread horizontal pleiotropy in causal relationships inferred from Mendelian randomization between complex traits and diseases. Nat Genet 2018;50(5):693–698.
[38] Xiang LL, Cao YT, Sun J, et al. Association between thyroid function and nonalcoholic fatty liver disease: a dose-response meta-analysis. Front Endocrinol 2024;15:1399517.
[39] Qiu S, Cao P, Guo Y, et al. Exploring the causality between hypothyroidism and non-alcoholic fatty liver: a Mendelian randomization study. Front Cell Dev Biol 2021;9:643582.
[40] D'Ambrosio R, Campi I, Maggioni M, et al. The relationship between liver histology and thyroid function tests in patients with non-alcoholic fatty liver disease (NAFLD). PLoS ONE 2021;16(4):e0249614.
41 Wang B, Wang B, Yang Y, et al. Thyroid function and non-alcoholic fatty liver disease in hyperthyroidism patients. BMC Endocr Disord 2021;21(1):27.

Keywords: Hyperthyroidism; Hypothyroidism; Liver disease.

Received 22 October 2024; received in revised form 18 February 2025; accepted 21 February 2025; Available online 26 February 2025

Journal of Hepatology, Volume *7*

Supplemental information

Thyroid dysfunction in MASLD: Results of a nationwide study

Shuai Yuan, Fahim Ebrahimi, David Bergman, Marijana Vujković, Eleonora Scorletti, Xixin Ruan, Jie Chen, Hannes Hagström, and Jonas F. Ludvigsson

Thyroid dysfunction in MASLD: Results of a nationwide study

Shuai Yuan, Fahim Ebrahimi, David Bergman, Marijana Vujković, Eleonora Scorletti,

Xixin Ruan, Jie Chen, Hannes Hagström, Jonas F. Ludvigsson

Table of contents

Table S1 2
Table S2 3
Table S3 4
Table S4 5
Table S5 7
Table S6 20
Table S7 21
Table S8 22
Table S9 23
Fig. S1. 24
Fig. S2. 25

Table S1. Study exclusion criteria

Excluded Conditions[1]	ICD-7 / ICD-8	ICD-9*	ICD-10
Alcohol abuse/misuse or Alcohol-related liver disease	280,00; 281,00; 307,00; 307,10; 307,99; 322; 581,10; 583,10; 261,00; 262,00; 291; 291,1; 303; 571,00; 571,01; 979; 980,00; 980,01; 980,98; 980,99	291; 294A; 303; 305A; 357F; 425F; 535D; 571A-D; 760W; 790D; 977D; 980A; 980X; V97B	E24.4; F10; G31.2; G62.1; G72.1; I42.6; K29.2; K70; K86.0; Q35.4; R78.0; T51.0; T51.8; T51.9; X65; Y15; Y57.3; Y90; Y91; Z50.2; Z71.4; Z71.2
Other abuse- and drug-related diagnoses	571,0; E860; N980	571A-D	F11-F19
Drug-induced liver disease	–	573D	K71
Viral hepatitis (e.g., hepatitis B, C)	070; 999,20	070	B15-19; B00.8; B25.1
Budd-Chiari	–	453A	I82
Liver abscess	572	572A	K75.0; A06.4
HIV	079,83; Y40,49; Y41,49	279K	B20-B24
Haemochromatosis	273,2	275A	E83.1
Wilson's disease	273,3	275B	E83.0
Autoimmune hepatitis	–	573D; 571E	K75.4
Primary biliary cholangitis	–	571G	K74.3; K74.4
Other cholangitis	574,06	576B	K83; K83.0A
Alpha-1 antitrypsin deficiency	–	277G	E88.0
Glycogen storage disease	–	271	E74
Liver transplantation†	–	V42H	Z94.4<br>JJC, DJ005; DJ006†
Gastric bypass surgery	–	–	JDF†

†Liver transplantation and Bariatric surgery were further defined via procedure codes.
[1] We excluded any person with a diagnosis of another aetiology of liver disease, or alcohol abuse/misuse or alcohol-related liver disease, defined on or before the index date.
*Note that in the Swedish versions of ICD-9, the fourth position of the code is denoted by a letter, and not by a digit. This letter corresponds alphabetically to the fourth digit that is used in US versions of ICD (for example, 571A corresponds to 571.0).
Abbreviations: HIV, Human Immunodeficiency Virus; ICD, International Classification of Disease.

Table S2: Definition of MASLD subgroups according to histology

Subgroup	SNOMED and ICD codes	
	Inclusion	Exclusion
Cirrhosis	M4950x	
MASLD with non-cirrhotic fibrosis<br><br>(note that this may or may not include NASH)	Steatosis: M5008x or M5520x, PLUS at least one fibrosis code: M49 [exactly], M4900x or M49060.	Cirrhosis: M495 [exactly] or M4950x.
NASH without fibrosis	Steatosis: either M5008x or M5520x, PLUS at least one of the following:<br><br>1. any M4- code, or<br><br>2. M5400x	M4 defines a broad category of inflammation, both acute and chronic.<br><br>Cannot have any of the following:<br><br>Fibrosis codes (M49 [exactly], M4900x or M49060) OR<br><br>Cirrhosis: M495 [exactly] or M4950x.
Simple steatosis	M5008x or M5520x	Cannot have any of the following:<br><br>Inflammation codes: M4- or M5400x OR<br><br>Fibrosis: M49 [exactly], M4900x or M49060 OR<br><br>Cirrhosis: M495 [exactly] or M4950x

Abbreviations: ICD, International Classification of Diseases; NASH, Nonalcoholic steatohepatitis; SNOMED, Systematized Nomenclature of Medicine.

Table S3. Definitions of covariates

Covariate	ICD8 (1969-1986)	ICD9 (1987-1996)	ICD10 (1997-)	ATC code
Pernicious anemia	281	281A	D51	
Diabetes mellitus	250<br><br>Or any of the ATC medication codes (see right-most column)	250<br><br>Or any of the ATC medication codes (see right-most column)	E10-E14<br>(Note: E14 also includes E14.0-E14.9)<br>Or any of the ATC medication codes (see right-most column)	A10A<br>A10AB-AE<br>A10BA-BX
Obesity	278<br>649,1	278<br>649B	E650-E679<br>E65<br>E66	
Dyslipidemia	272;<br>Or any of the ATC medication codes	272<br>Or any of the ATC medication codes	E78<br>Or any of the ATC medication codes	C10AA<br>C10BA<br>C10BX<br>C10AB<br>C10AC
Hypertension	400-404	401-405	I10-I13, I15	C09
COPD (≥40 years)		491, 492, 496	J41, 42,43,44	

COPD. Chronic obstructive Pulmonary Disease.

Table S4. Definitions of autoimmune diseases*

Autoimmune disease	ICD code
Systemic/cutaneous lupus erythematosus	– ICD-8: 734,1<br>– ICD-9: 710A<br>– ICD-10: M32, L931
Vitiligo	– ICD-8: 709,05<br>– ICD-9: -<br>– ICD-10: L80
Psoriasis	– ICD-8: 696,0 and 696,1<br>– ICD-9: 696B<br>– ICD-10: L40
Alopecia areata	– ICD-8: 704,00<br>– ICD-9: -<br>– ICD-10: L63
Systemic sclerosis	– ICD-8: 734,00-734,19<br>– ICD-9: 710B<br>– ICD-10: M34
Dermatomyositis or juvenile dermatomyositis	– ICD-8: 716,00<br>– ICD-9: 710D<br>– ICD-10: M33 except M33.2
Polymyositis	– ICD-8: 716,10<br>– ICD-9: 710E<br>– ICD-10: M33.2
Type 1 diabetes	– ICD-8: 250 (limited to first diagnosis ≤30 years)<br>– ICD-9: 250 (limited to first diagnosis ≤30 years)<br>– ICD-10: E10
Addison´s disease	– ICD-8: 255,10<br>– ICD-9: 255E<br>– ICD-10: E27.1 and E27.2
Autoimmune hepatitis	– ICD-8: 573,0 and 571,9<br>– ICD-9: 573D, 573E<br>– ICD-10: K75.4
Primary biliary cholangitis	– ICD-8: -<br>– ICD-9: 571G<br>– ICD-10: K74.3
Inflammatory bowel disease	– ICD8: 569,04; 563,1; 563,10; 569,02; 563,00<br>– ICD-9: 555, 556<br>– ICD-10: K50, K51 and K52.3
Sjögren syndrome	– ICD-8: 734,90

	– ICD-9: 710C<br>– ICD-10: M35.0
Myasthenia gravis	– ICD-8: 733,00<br>– ICD-9: 358A<br>– ICD-10: G70.0
Rheumatoid arthritis	– ICD-8: 712,10; 712,20; 712,38; 712,39<br>– ICD-9: 714A; 714B; 714C; 714W; 719D<br>– ICD-10: M05; M06.0; M06.2; M06.3, M06.8; M06.9; M12.3
Multiple sclerosis	– ICD-8: 340,99<br>– ICD-9: 340<br>– ICD-10: G35
Ankylosing spondylitis	– ICD-8: 712,40<br>– ICD-9: 720A<br>– ICD-10: M45
Spondyloarthritis	– ICD8: 720,1 and 720,2<br>– ICD-9: 720A; 696A; 713B; 099D; 711A; 720B; 720C; 720W<br>– ICD-10: M45; M08.1; L40.5; M07.0-3; M07.4-5; M07.6; M09.1.2; M02.0-2; M02.3; M02.8-9; M46.0/M46.1/8/9
Sarcoidosis	– ICD-8: 135<br>– ICD-9: 135<br>– ICD-10: D86; G53.2; M63.3
Celiac disease	– ICD-8: 269,00<br>– ICD-9: 579A<br>– ICD-10: K90.0

* Code reference: Yuan S, Leffler D, Lebwohl B, Green PHR, Larsson SC, Söderling J, Sun J, Ludvigsson JF. Older age of celiac disease diagnosis and risk of autoimmune disease: A nationwide matched case-control study. J Autoimmun. 2024;143:103170.

Table S5. Genetic instruments for TSH, hypothyroidism, and hyperthyroidism

Exposure	SNP	EA	EAF	Beta	SE	P value	F statistic
TSH	rs4268443	A	0.31	-0.017	0.003	4.26E-08	30
TSH	rs10748781	A	0.57	-0.059	0.003	1.97E-87	387
TSH	rs11190164	A	0.74	-0.020	0.003	1.69E-09	37
TSH	rs1931666	A	0.69	-0.018	0.003	3.52E-08	30
TSH	rs12783458	C	0.63	0.023	0.003	5.57E-14	57
TSH	rs17481499	T	0.17	0.046	0.004	9.13E-33	146
TSH	rs11255770	T	0.24	-0.024	0.003	1.16E-12	50
TSH	rs4933466	A	0.60	0.033	0.003	2.67E-28	121
TSH	rs4934383	T	0.66	-0.023	0.003	8.64E-14	54
TSH	rs12411841	T	0.97	-0.055	0.008	1.32E-11	46
TSH	rs74451129	T	0.28	-0.024	0.004	1.77E-11	45
TSH	rs7417283	T	0.80	0.033	0.004	1.78E-19	80
TSH	rs17020127	A	0.91	-0.103	0.005	1.57E-88	405
TSH	rs11605461	A	0.48	-0.039	0.003	6.91E-41	179
TSH	rs6590334	T	0.48	0.017	0.003	5.69E-09	34
TSH	rs11221835	T	0.23	-0.022	0.003	1.33E-10	42
TSH	rs11602347	C	0.60	-0.024	0.003	9.20E-15	59
TSH	rs179785	A	0.48	0.018	0.003	4.35E-10	39
TSH	rs11038357	A	0.28	-0.065	0.003	1.26E-92	415
TSH	rs11204756	A	0.50	-0.019	0.003	3.91E-11	44
TSH	rs2581928	A	0.27	-0.022	0.003	8.69E-12	45
TSH	rs6669935	C	0.43	0.018	0.003	1.91E-09	37
TSH	rs2229738	T	0.09	-0.039	0.006	8.41E-13	51
TSH	rs523587	T	0.43	0.016	0.003	2.29E-08	32
TSH	rs56279106	A	0.27	-0.020	0.003	2.03E-09	37
TSH	rs10751136	A	0.50	0.019	0.003	3.89E-11	43
TSH	rs1138328	C	0.72	0.019	0.003	1.57E-08	33
TSH	rs2147948	A	0.18	-0.028	0.004	7.99E-13	51
TSH	rs7529294	T	0.54	-0.018	0.003	1.16E-09	37
TSH	rs139260332	A	0.01	-0.100	0.015	7.37E-12	47
TSH	rs12091047	T	0.35	0.067	0.003	8.98E-106	466
TSH	rs10799824	A	0.16	-0.119	0.004	5.12E-194	881
TSH	rs12133889	A	0.39	0.029	0.003	2.21E-22	93
TSH	rs4844563	A	0.67	-0.017	0.003	3.54E-08	30
TSH	rs1920413	A	0.46	0.016	0.003	4.84E-08	29
TSH	rs3184504	T	0.49	0.030	0.003	2.47E-25	108
TSH	rs10743983	A	0.55	-0.018	0.003	7.23E-10	39
TSH	rs1690789	T	0.48	-0.029	0.003	5.11E-23	99
TSH	rs2807367	C	0.67	0.020	0.003	6.07E-11	42
TSH	rs11830037	A	0.08	0.040	0.006	3.42E-13	53
TSH	rs182307407	A	0.03	0.052	0.009	7.07E-09	33

TSH	rs7315310	A	0.40	-0.020	0.003	1.25E-11	46
TSH	rs7955258	A	0.42	-0.047	0.003	1.97E-54	249
TSH	rs7975994	C	0.92	0.040	0.005	6.09E-14	56
TSH	rs12826041	A	0.37	-0.018	0.003	3.67E-09	34
TSH	rs71447494	A	0.84	-0.027	0.004	1.07E-10	42
TSH	rs11836059	A	0.74	-0.019	0.003	2.15E-08	31
TSH	rs149363012	T	0.02	0.125	0.012	1.90E-26	114
TSH	rs61916657	A	0.32	-0.019	0.003	1.18E-09	36
TSH	rs10878984	T	0.35	-0.021	0.003	2.08E-12	50
TSH	rs61938844	A	0.03	0.172	0.010	5.52E-71	315
TSH	rs10735341	A	0.13	-0.042	0.004	1.52E-22	94
TSH	rs9521826	A	0.73	0.025	0.003	1.88E-14	57
TSH	rs17462267	A	0.73	-0.037	0.003	6.08E-29	122
TSH	rs9537312	T	0.21	-0.024	0.004	4.37E-11	43
TSH	rs10914681	T	0.25	0.023	0.003	2.34E-11	44
TSH	rs9532662	T	0.31	0.019	0.003	3.17E-09	36
TSH	rs568995	A	0.21	0.034	0.004	7.42E-22	94
TSH	rs4885687	T	0.73	0.026	0.003	5.96E-15	61
TSH	rs12889167	T	0.55	0.039	0.003	1.20E-40	181
TSH	rs12436555	A	0.16	-0.022	0.004	3.54E-08	31
TSH	rs6680638	T	0.47	-0.016	0.003	4.08E-08	30
TSH	rs7145546	C	0.55	-0.035	0.003	7.00E-34	148
TSH	rs148291250	A	0.99	0.137	0.019	1.33E-12	50
TSH	rs116909374	T	0.04	-0.159	0.009	1.11E-78	349
TSH	rs12891811	T	0.58	-0.022	0.003	1.67E-13	53
TSH	rs71405769	A	0.03	0.057	0.008	6.16E-12	48
TSH	rs117266160	A	0.03	-0.060	0.009	1.46E-11	46
TSH	rs61981267	T	0.02	0.072	0.013	9.88E-09	33
TSH	rs10132220	A	0.07	-0.045	0.006	2.61E-15	63
TSH	rs78218384	T	0.91	-0.066	0.005	1.01E-38	173
TSH	rs2234919	A	0.06	-0.053	0.006	2.71E-18	77
TSH	rs75235317	A	0.06	0.049	0.006	5.13E-15	61
TSH	rs8022931	C	0.54	-0.049	0.003	1.58E-62	280
TSH	rs117655019	T	0.03	-0.082	0.010	6.11E-17	70
TSH	rs2371566	T	0.08	-0.044	0.005	2.55E-16	67
TSH	rs112123737	T	0.06	-0.038	0.006	3.89E-09	34
TSH	rs77303590	T	0.03	0.068	0.010	1.72E-12	49
TSH	rs2295727	A	0.93	-0.033	0.006	1.51E-08	32
TSH	rs768356	T	0.80	-0.074	0.004	1.35E-92	424
TSH	rs12567744	A	0.68	0.036	0.003	1.15E-30	135
TSH	rs78218421	T	0.06	0.044	0.006	9.05E-13	50
TSH	rs1288492	T	0.46	0.019	0.003	2.94E-10	41
TSH	rs1044474	A	0.55	0.026	0.003	9.71E-19	78
TSH	rs17477923	T	0.75	0.061	0.003	7.59E-75	341

TSH	rs6494466	A	0.26	-0.020	0.003	2.08E-09	36
TSH	rs11857151	C	0.68	0.049	0.003	6.56E-57	250
TSH	rs30233	A	0.57	-0.032	0.003	1.53E-27	114
TSH	rs334699	A	0.06	-0.145	0.007	6.89E-98	440
TSH	rs17305041	A	0.92	0.036	0.006	3.07E-09	35
TSH	rs7203093	A	0.91	-0.038	0.005	8.47E-14	55
TSH	rs11641216	A	0.61	0.016	0.003	4.96E-08	29
TSH	rs1045476	A	0.18	0.043	0.004	2.55E-29	124
TSH	rs4785953	A	0.87	-0.032	0.004	7.54E-14	57
TSH	rs524770	A	0.39	0.023	0.003	2.89E-15	61
TSH	rs57186408	T	0.03	0.049	0.009	2.11E-08	31
TSH	rs73575083	A	0.67	0.092	0.003	3.03E-199	887
TSH	rs11864793	C	0.91	-0.044	0.006	1.80E-14	60
TSH	rs117544538	A	0.94	0.036	0.006	1.85E-08	32
TSH	rs12036629	A	0.53	-0.032	0.003	1.25E-27	119
TSH	rs55938136	A	0.78	-0.038	0.004	1.50E-23	99
TSH	rs199528	T	0.20	0.040	0.004	4.99E-29	125
TSH	rs35073649	T	0.38	0.025	0.003	4.56E-17	68
TSH	rs113599227	T	0.04	-0.084	0.007	9.31E-30	128
TSH	rs1801690	C	0.94	0.056	0.006	3.18E-19	80
TSH	rs9889941	A	0.70	0.018	0.003	7.30E-09	33
TSH	rs1042673	A	0.54	-0.043	0.003	9.41E-50	220
TSH	rs4793439	T	0.47	0.041	0.003	3.57E-45	197
TSH	rs879736	T	0.17	0.026	0.004	4.24E-11	44
TSH	rs112719042	C	0.24	-0.021	0.004	3.02E-08	31
TSH	rs6567094	A	0.55	0.031	0.003	2.22E-26	114
TSH	rs72978712	T	0.79	-0.038	0.004	3.79E-22	96
TSH	rs1496627	A	0.58	0.021	0.003	5.31E-13	53
TSH	rs11670562	T	0.74	-0.020	0.003	2.92E-09	35
TSH	rs4807021	A	0.30	0.024	0.003	7.64E-14	57
TSH	rs76978239	T	0.09	-0.031	0.005	1.00E-08	33
TSH	rs11672947	T	0.39	-0.024	0.003	1.36E-14	58
TSH	rs3848573	T	0.81	0.032	0.004	1.28E-17	73
TSH	rs7248104	A	0.41	-0.058	0.003	3.20E-87	379
TSH	rs12974053	T	0.04	0.048	0.007	6.45E-11	42
TSH	rs141735698	A	0.96	0.076	0.009	7.50E-19	79
TSH	rs1203930	A	0.22	-0.065	0.004	1.08E-78	347
TSH	rs1883806	T	0.39	0.020	0.003	5.21E-10	38
TSH	rs28482886	A	0.71	0.026	0.003	1.53E-15	63
TSH	rs139242164	T	0.01	0.116	0.016	2.55E-13	54
TSH	rs55830103	T	0.82	-0.028	0.004	2.52E-12	50
TSH	rs237080	T	0.29	-0.023	0.003	5.99E-12	46
TSH	rs6133344	T	0.51	-0.024	0.003	1.43E-16	68
TSH	rs11675342	T	0.42	0.028	0.003	1.34E-21	94

TSH	rs79259951	A	0.93	0.037	0.006	1.39E-10	41
TSH	rs6414089	A	0.36	0.018	0.003	6.44E-09	34
TSH	rs4471862	A	0.32	-0.025	0.003	2.40E-15	64
TSH	rs3792198	A	0.56	0.017	0.003	7.69E-09	34
TSH	rs62114518	T	0.31	-0.020	0.003	3.98E-09	35
TSH	rs28484879	T	0.92	0.031	0.005	7.83E-09	33
TSH	rs2288188	T	0.96	0.072	0.008	6.16E-20	83
TSH	rs2364727	T	0.11	0.027	0.005	1.30E-08	32
TSH	rs4340549	T	0.76	-0.023	0.004	1.45E-11	44
TSH	rs4274624	T	0.77	-0.020	0.004	9.34E-09	34
TSH	rs62189580	C	0.60	0.018	0.003	4.36E-09	35
TSH	rs56092701	T	0.09	0.045	0.005	7.79E-19	78
TSH	rs737308	T	0.28	-0.093	0.003	1.02E-177	786
TSH	rs6724073	T	0.73	0.051	0.004	3.35E-48	209
TSH	rs7589228	A	0.63	0.026	0.003	2.43E-18	77
TSH	rs5997969	T	0.33	0.025	0.003	3.99E-16	66
TSH	rs62192963	A	0.15	0.032	0.004	6.44E-15	60
TSH	rs113360717	T	0.03	0.081	0.008	1.38E-23	101
TSH	rs10186921	T	0.56	0.038	0.003	1.05E-38	173
TSH	rs6546566	T	0.69	0.018	0.003	6.65E-09	34
TSH	rs1661584	T	0.75	-0.048	0.003	2.66E-48	214
TSH	rs2439737	T	0.51	-0.017	0.003	4.63E-09	34
TSH	rs55717031	T	0.31	-0.020	0.003	4.94E-10	39
TSH	rs9857136	A	0.42	-0.032	0.003	1.63E-27	120
TSH	rs7651090	A	0.69	0.048	0.003	2.38E-52	236
TSH	rs59381142	A	0.24	-0.050	0.004	1.03E-46	207
TSH	rs6805350	T	0.94	-0.035	0.006	2.95E-08	31
TSH	rs115044883	A	0.08	-0.033	0.006	1.19E-08	33
TSH	rs17450274	A	0.29	0.027	0.003	8.33E-17	69
TSH	rs115371738	A	0.02	0.062	0.011	7.58E-09	34
TSH	rs13138273	A	0.80	0.111	0.004	1.00E-200	895
TSH	rs76697438	A	0.01	-0.104	0.015	4.75E-12	48
TSH	rs524424	A	0.56	-0.018	0.003	2.23E-09	35
TSH	rs1385737	T	0.13	-0.036	0.005	2.47E-14	59
TSH	rs11732564	A	0.28	-0.025	0.003	6.50E-15	59
TSH	rs1458819	T	0.89	0.028	0.005	6.34E-09	33
TSH	rs155943	A	0.47	0.019	0.003	4.11E-11	43
TSH	rs77994712	C	0.94	0.062	0.006	6.13E-22	94
TSH	rs6898408	C	0.34	0.018	0.003	7.57E-09	33
TSH	rs28567918	T	0.80	-0.022	0.004	2.19E-08	31
TSH	rs440225	A	0.71	-0.022	0.004	3.07E-08	31
TSH	rs62363038	A	0.77	0.026	0.003	3.90E-14	58
TSH	rs10075093	T	0.21	-0.028	0.004	7.38E-15	61
TSH	rs7707827	A	0.10	0.035	0.005	9.20E-12	46

TSH	rs115827777	T	0.02	0.096	0.012	7.61E-17	70
TSH	rs79211479	C	0.97	-0.068	0.009	5.25E-13	52
TSH	rs2928167	A	0.86	0.133	0.004	1.00E-200	957
TSH	rs9687206	A	0.57	-0.136	0.003	1.00E-200	2183
TSH	rs151297408	T	0.01	-0.091	0.014	1.50E-10	41
TSH	rs142891552	T	0.02	0.069	0.011	2.57E-10	40
TSH	rs148079416	C	0.98	0.085	0.012	5.95E-13	52
TSH	rs13436203	A	0.97	-0.069	0.008	9.80E-17	69
TSH	rs16874254	T	0.06	-0.065	0.007	1.28E-22	95
TSH	rs4273585	A	0.44	-0.043	0.003	3.11E-48	215
TSH	rs115236194	A	0.98	-0.066	0.012	1.48E-08	32
TSH	rs1969151	T	0.21	0.020	0.004	1.24E-08	33
TSH	rs1114707	T	0.34	0.021	0.003	3.03E-11	45
TSH	rs6901783	A	0.74	-0.019	0.003	6.79E-09	33
TSH	rs9497965	T	0.40	0.033	0.003	9.03E-29	128
TSH	rs79708723	A	0.03	0.068	0.009	5.14E-14	57
TSH	rs7758026	C	0.88	0.050	0.005	3.14E-28	119
TSH	rs73022136	T	0.03	0.072	0.010	2.91E-12	48
TSH	rs56228667	T	0.85	0.097	0.004	3.98E-122	557
TSH	rs1033701	A	0.27	-0.117	0.003	1.00E-200	1332
TSH	rs117801821	C	0.97	-0.068	0.011	1.27E-09	37
TSH	rs146760552	T	0.02	0.082	0.014	9.20E-09	33
TSH	rs12199716	A	0.02	0.109	0.014	4.99E-16	65
TSH	rs375911171	T	0.02	0.081	0.014	3.68E-09	35
TSH	rs34471367	A	0.96	-0.043	0.008	3.22E-08	31
TSH	rs9364808	T	0.92	0.033	0.006	4.84E-09	35
TSH	rs893310	T	0.53	-0.021	0.003	6.08E-13	51
TSH	rs62425402	T	0.11	-0.030	0.005	1.12E-10	41
TSH	rs751171	T	0.66	-0.036	0.003	9.06E-31	133
TSH	rs9396865	T	0.84	-0.030	0.004	4.57E-14	58
TSH	rs16895218	A	0.94	0.041	0.007	2.82E-10	39
TSH	rs1265091	T	0.18	0.045	0.004	2.79E-29	126
TSH	rs9461666	T	0.97	-0.067	0.011	5.14E-10	39
TSH	rs1129735	T	0.33	0.039	0.003	2.01E-30	134
TSH	rs184252375	T	0.29	0.038	0.005	4.11E-14	56
TSH	rs2396083	C	0.68	0.097	0.003	1.00E-200	921
TSH	rs34046483	A	0.08	0.078	0.006	4.12E-41	179
TSH	rs77547428	T	0.98	-0.105	0.015	4.85E-13	52
TSH	rs66760320	T	0.25	-0.078	0.003	7.94E-120	528
TSH	rs2031365	T	0.28	0.049	0.003	5.37E-52	231
TSH	rs62621812	A	0.03	0.080	0.010	2.08E-16	68
TSH	rs55837101	T	0.22	0.020	0.004	1.75E-08	32
TSH	rs706024	A	0.61	-0.020	0.003	3.16E-11	44
TSH	rs4318967	T	0.59	0.019	0.003	3.84E-10	38

TSH	rs10950803	A	0.55	0.018	0.003	2.82E-09	36
TSH	rs700750	A	0.62	0.031	0.003	3.15E-25	105
TSH	rs1875057	A	0.37	0.017	0.003	8.74E-09	33
TSH	rs10263288	A	0.89	-0.032	0.005	5.37E-12	48
TSH	rs3134107	A	0.18	-0.023	0.004	1.11E-09	37
TSH	rs72682433	T	0.90	-0.035	0.005	2.32E-13	53
TSH	rs78404781	A	0.99	0.183	0.016	2.86E-32	140
TSH	rs114322847	T	0.03	-0.161	0.010	2.84E-61	275
TSH	rs28640053	A	0.17	0.028	0.004	2.94E-13	52
TSH	rs56009477	A	0.85	0.052	0.004	2.62E-38	169
TSH	rs66963240	T	0.43	-0.038	0.003	6.98E-40	174
TSH	rs73234168	A	0.26	-0.061	0.003	1.43E-75	336
TSH	rs62506639	T	0.81	-0.027	0.004	1.47E-13	54
TSH	rs9772642	T	0.70	-0.029	0.003	1.84E-19	79
TSH	rs67639219	A	0.33	-0.020	0.003	4.44E-11	43
TSH	rs396483	T	0.30	0.021	0.003	7.73E-11	41
TSH	rs2921053	C	0.46	-0.025	0.003	1.48E-16	66
TSH	rs17729624	A	0.10	0.034	0.005	1.72E-12	50
TSH	rs925489	T	0.65	0.075	0.003	4.50E-138	623
TSH	rs182020360	T	0.98	-0.064	0.011	5.84E-09	34
TSH	rs4743168	A	0.63	-0.017	0.003	2.47E-08	31
TSH	rs10818666	T	0.73	-0.019	0.003	9.46E-09	33
TSH	rs954585	A	0.65	-0.020	0.003	3.04E-11	44
TSH	rs7853472	A	0.38	0.028	0.003	2.51E-21	93
TSH	rs734638	C	0.70	0.020	0.003	6.50E-10	37
TSH	rs554833	T	0.35	0.042	0.003	8.54E-44	194
TSH	rs4917375	A	0.44	0.017	0.003	1.74E-08	32
TSH	rs9298749	A	0.61	-0.032	0.003	3.50E-26	112
TSH	rs10814737	T	0.95	-0.054	0.007	2.98E-14	58
TSH	rs806028	A	0.08	0.029	0.005	4.87E-08	30
TSH	rs17209669	T	0.25	0.025	0.003	3.28E-14	58
TSH	rs7864565	A	0.41	-0.036	0.003	1.56E-34	153
TSH	rs10122640	A	0.09	0.036	0.005	1.13E-12	50
TSH	rs10814915	T	0.44	0.052	0.003	8.80E-70	317
TSH	rs4741961	T	0.84	-0.033	0.004	5.02E-16	64
TSH	rs7875193	T	0.35	0.017	0.003	4.08E-08	29
TSH	rs13286806	A	0.42	-0.018	0.003	1.42E-09	36
Hypothyroidism	rs10119187	T	0.82	-0.054	0.007	5.48E-14	57
Hypothyroidism	rs10180164	A	0.97	-0.088	0.016	4.27E-08	30
Hypothyroidism	rs10407062	T	0.54	0.037	0.006	3.24E-11	44
Hypothyroidism	rs1042127	A	0.82	0.048	0.008	1.05E-08	33
Hypothyroidism	rs1042140	A	0.77	-0.039	0.007	8.58E-09	33
Hypothyroidism	rs1050979	A	0.50	-0.039	0.006	3.24E-12	49
Hypothyroidism	rs10735341	A	0.17	-0.047	0.008	2.04E-08	31

Hypothyroidism	rs1074849	G	0.77	-0.043	0.007	8.29E-11	42
Hypothyroidism	rs10748781	C	0.44	0.060	0.006	1.73E-24	104
Hypothyroidism	rs10751648	T	0.43	0.034	0.006	1.97E-09	36
Hypothyroidism	rs10753774	C	0.71	-0.032	0.006	4.39E-08	30
Hypothyroidism	rs10768126	A	0.60	-0.043	0.006	1.07E-13	55
Hypothyroidism	rs10775303	G	0.48	0.031	0.006	4.49E-08	30
Hypothyroidism	rs1079418	A	0.69	0.102	0.006	9.00E-61	270
Hypothyroidism	rs10814915	T	0.44	0.063	0.006	3.26E-29	126
Hypothyroidism	rs10818037	G	0.44	-0.181	0.006	1.00E-200	916
Hypothyroidism	rs10887774	G	0.73	0.043	0.006	3.09E-12	49
Hypothyroidism	rs10905430	C	0.58	0.048	0.006	1.79E-15	63
Hypothyroidism	rs10910095	G	0.86	-0.051	0.007	3.58E-12	48
Hypothyroidism	rs10917470	G	0.50	-0.096	0.006	4.35E-64	286
Hypothyroidism	rs11073716	C	0.62	0.034	0.006	1.53E-09	36
Hypothyroidism	rs11181289	C	0.62	0.033	0.006	4.54E-08	30
Hypothyroidism	rs11185131	A	0.47	0.037	0.006	3.06E-09	35
Hypothyroidism	rs112165453	C	0.97	-0.116	0.019	1.44E-09	37
Hypothyroidism	rs112614658	A	0.94	-0.068	0.012	1.14E-08	33
Hypothyroidism	rs112880194	G	0.96	0.090	0.016	7.56E-09	33
Hypothyroidism	rs113135100	G	0.87	0.049	0.009	4.02E-08	30
Hypothyroidism	rs113170275	G	0.68	0.037	0.006	3.89E-09	35
Hypothyroidism	rs113317416	C	0.88	-0.104	0.011	8.52E-22	92
Hypothyroidism	rs113866322	T	0.63	0.040	0.006	3.36E-12	48
Hypothyroidism	rs114786190	G	0.92	-0.067	0.011	4.80E-10	39
Hypothyroidism	rs11603616	G	0.68	0.033	0.006	4.02E-08	30
Hypothyroidism	rs11666808	T	0.38	-0.033	0.006	4.58E-08	30
Hypothyroidism	rs11675342	C	0.58	-0.154	0.006	7.18E-159	721
Hypothyroidism	rs11683914	T	0.46	0.057	0.006	7.45E-22	92
Hypothyroidism	rs11687741	C	0.65	0.038	0.006	4.52E-11	43
Hypothyroidism	rs116909374	C	0.97	0.143	0.019	9.68E-14	55
Hypothyroidism	rs11718237	G	0.56	0.036	0.006	3.08E-10	40
Hypothyroidism	rs11724128	T	0.69	-0.038	0.007	3.74E-08	30
Hypothyroidism	rs117591891	C	0.98	-0.158	0.025	1.86E-10	41
Hypothyroidism	rs117744081	A	0.97	-0.089	0.016	2.13E-08	31
Hypothyroidism	rs11830037	C	0.92	-0.063	0.011	5.09E-09	34
Hypothyroidism	rs12046347	G	0.41	-0.035	0.006	2.42E-09	36
Hypothyroidism	rs12134155	C	0.37	0.047	0.006	4.95E-16	66
Hypothyroidism	rs12144309	C	0.81	0.044	0.007	6.20E-11	43
Hypothyroidism	rs12187443	T	0.68	0.041	0.006	2.14E-11	45
Hypothyroidism	rs12357667	G	0.59	0.048	0.006	1.03E-16	69
Hypothyroidism	rs1239704	G	0.20	0.047	0.007	2.86E-11	44
Hypothyroidism	rs12636552	A	0.69	-0.032	0.006	3.94E-08	30
Hypothyroidism	rs12893151	C	0.79	0.044	0.007	4.99E-10	39
Hypothyroidism	rs12904211	T	0.66	0.039	0.006	2.78E-10	40

Hypothyroidism	rs12928973	C	0.89	0.049	0.009	2.97E-08	31
Hypothyroidism	rs12937081	A	0.86	0.050	0.008	5.29E-10	39
Hypothyroidism	rs12952267	G	0.76	0.038	0.007	1.68E-08	32
Hypothyroidism	rs13075906	C	0.60	0.074	0.006	4.87E-35	153
Hypothyroidism	rs1317085	C	0.67	0.035	0.006	3.21E-08	31
Hypothyroidism	rs1320344	A	0.31	0.036	0.006	2.38E-10	40
Hypothyroidism	rs13209358	C	0.92	-0.092	0.010	1.05E-19	83
Hypothyroidism	rs13250295	C	0.70	0.045	0.006	5.42E-13	52
Hypothyroidism	rs13399762	A	0.95	-0.071	0.012	6.64E-09	34
Hypothyroidism	rs142127908	A	0.47	0.048	0.008	4.78E-09	34
Hypothyroidism	rs142647938	C	0.98	0.124	0.022	1.97E-08	32
Hypothyroidism	rs143104579	G	0.98	0.128	0.023	1.45E-08	32
Hypothyroidism	rs145124960	C	0.98	-0.140	0.022	2.42E-10	40
Hypothyroidism	rs145269503	G	0.98	0.116	0.020	6.88E-09	34
Hypothyroidism	rs1470579	A	0.68	0.065	0.006	1.10E-28	123
Hypothyroidism	rs147563297	G	0.99	-0.197	0.024	8.09E-17	69
Hypothyroidism	rs147610574	T	0.96	-0.086	0.015	3.52E-09	35
Hypothyroidism	rs1479567	G	0.62	-0.125	0.006	2.00E-102	462
Hypothyroidism	rs148554863	T	0.98	0.205	0.022	7.09E-20	83
Hypothyroidism	rs149875147	G	0.93	-0.066	0.012	1.36E-08	32
Hypothyroidism	rs151190323	C	0.91	-0.061	0.010	8.49E-10	38
Hypothyroidism	rs151213404	G	0.61	0.062	0.006	6.90E-26	111
Hypothyroidism	rs151233	C	0.87	-0.068	0.008	4.89E-16	66
Hypothyroidism	rs1534424	A	0.61	0.055	0.006	1.49E-20	86
Hypothyroidism	rs1567124	C	0.70	-0.036	0.006	2.08E-08	31
Hypothyroidism	rs16899682	G	0.98	-0.120	0.018	1.81E-11	45
Hypothyroidism	rs1702877	C	0.68	-0.050	0.006	3.63E-17	71
Hypothyroidism	rs17111361	T	0.88	0.058	0.008	5.01E-12	48
Hypothyroidism	rs17194601	T	0.87	0.054	0.009	4.59E-09	34
Hypothyroidism	rs17494319	C	0.79	0.042	0.007	1.18E-08	33
Hypothyroidism	rs17664732	T	0.73	0.069	0.006	4.00E-27	116
Hypothyroidism	rs17676218	C	0.77	0.037	0.007	2.79E-08	31
Hypothyroidism	rs177055	G	0.36	-0.033	0.006	7.19E-09	33
Hypothyroidism	rs17770799	T	0.85	0.073	0.008	8.16E-21	88
Hypothyroidism	rs185985460	C	0.98	-0.134	0.020	2.01E-11	45
Hypothyroidism	rs1868167	A	0.22	0.055	0.007	4.18E-16	66
Hypothyroidism	rs189508974	T	0.97	0.102	0.018	1.75E-08	32
Hypothyroidism	rs192874879	G	0.98	-0.155	0.025	6.96E-10	38
Hypothyroidism	rs196047	A	0.39	-0.032	0.006	4.32E-08	30
Hypothyroidism	rs1998616	A	0.72	-0.041	0.006	2.87E-12	49
Hypothyroidism	rs2016105	G	0.98	-0.260	0.024	1.04E-26	114
Hypothyroidism	rs2056625	G	0.61	-0.033	0.006	4.73E-08	30
Hypothyroidism	rs2061914	A	0.91	-0.059	0.010	1.42E-09	37
Hypothyroidism	rs2111485	A	0.40	-0.039	0.006	1.73E-11	45

Hypothyroidism	rs2160042	G	0.87	0.052	0.009	1.71E-09	36
Hypothyroidism	rs2250179	T	0.43	-0.036	0.006	8.14E-10	38
Hypothyroidism	rs229540	T	0.58	-0.051	0.006	3.46E-19	80
Hypothyroidism	rs2297163	T	0.81	-0.046	0.007	3.81E-11	44
Hypothyroidism	rs2314345	G	0.37	0.094	0.006	5.60E-59	262
Hypothyroidism	rs2374589	A	0.93	-0.061	0.011	3.07E-08	31
Hypothyroidism	rs2391836	C	0.73	0.068	0.006	1.15E-25	110
Hypothyroidism	rs2435204	A	0.77	-0.088	0.007	3.05E-39	172
Hypothyroidism	rs244689	A	0.14	0.054	0.008	2.86E-11	44
Hypothyroidism	rs2456451	G	0.66	0.070	0.006	6.23E-33	143
Hypothyroidism	rs2472540	G	0.54	-0.063	0.006	9.38E-29	124
Hypothyroidism	rs263660	A	0.72	0.036	0.006	2.28E-08	31
Hypothyroidism	rs2654188	T	0.39	0.051	0.006	3.12E-16	67
Hypothyroidism	rs2675662	A	0.58	0.032	0.006	4.03E-09	35
Hypothyroidism	rs2706119	A	0.10	0.054	0.010	3.53E-08	30
Hypothyroidism	rs2761063	C	0.48	-0.068	0.006	1.04E-32	142
Hypothyroidism	rs2783971	A	0.47	-0.044	0.006	7.05E-14	56
Hypothyroidism	rs2811985	C	0.31	-0.039	0.006	2.52E-09	36
Hypothyroidism	rs28558845	G	0.84	-0.052	0.007	2.46E-12	49
Hypothyroidism	rs28646006	C	0.79	0.100	0.009	1.31E-31	137
Hypothyroidism	rs2911460	A	0.31	0.036	0.006	8.78E-09	33
Hypothyroidism	rs2921053	G	0.55	0.071	0.006	6.57E-33	143
Hypothyroidism	rs2928166	T	0.87	0.070	0.009	5.64E-16	66
Hypothyroidism	rs30233	G	0.44	0.038	0.006	1.99E-10	40
Hypothyroidism	rs3087243	G	0.55	0.125	0.006	4.47E-109	492
Hypothyroidism	rs3134996	A	0.36	-0.145	0.007	3.37E-98	442
Hypothyroidism	rs3184504	T	0.49	0.132	0.006	2.19E-121	549
Hypothyroidism	rs329124	A	0.58	0.037	0.006	8.01E-10	38
Hypothyroidism	rs334719	A	0.07	-0.130	0.014	9.15E-20	83
Hypothyroidism	rs34048763	G	0.71	0.043	0.007	5.45E-11	43
Hypothyroidism	rs34292522	A	0.69	-0.048	0.006	8.85E-15	60
Hypothyroidism	rs34357460	G	0.67	0.054	0.006	5.90E-17	70
Hypothyroidism	rs34536443	G	0.95	0.084	0.013	2.86E-10	40
Hypothyroidism	rs34666276	T	0.63	-0.037	0.006	1.50E-10	41
Hypothyroidism	rs34787248	C	0.83	0.094	0.011	1.06E-17	73
Hypothyroidism	rs35000415	C	0.89	-0.053	0.009	2.60E-09	35
Hypothyroidism	rs35004101	T	0.96	0.096	0.016	2.67E-09	35
Hypothyroidism	rs35074907	G	0.98	-0.155	0.020	1.92E-14	59
Hypothyroidism	rs35484831	C	0.95	-0.071	0.013	2.66E-08	31
Hypothyroidism	rs369340113	G	0.56	0.065	0.008	5.44E-18	75
Hypothyroidism	rs3755397	A	0.90	0.061	0.009	1.12E-10	42
Hypothyroidism	rs3761959	C	0.56	-0.047	0.006	1.28E-16	68
Hypothyroidism	rs3796220	C	0.79	-0.043	0.007	1.26E-09	37
Hypothyroidism	rs3802214	T	0.19	0.048	0.007	6.31E-11	43

Hypothyroidism	rs3821374	A	0.23	0.045	0.008	3.43E-09	35
Hypothyroidism	rs3826884	G	0.82	-0.044	0.007	9.46E-10	37
Hypothyroidism	rs3850765	T	0.42	-0.034	0.006	3.37E-09	35
Hypothyroidism	rs41295121	C	0.99	0.191	0.030	1.64E-10	41
Hypothyroidism	rs41444548	C	0.93	0.103	0.011	1.01E-19	83
Hypothyroidism	rs4269168	C	0.53	-0.045	0.005	9.68E-17	69
Hypothyroidism	rs4409785	T	0.82	-0.069	0.007	2.59E-22	94
Hypothyroidism	rs4478401	G	0.62	0.039	0.006	1.63E-10	41
Hypothyroidism	rs4664302	T	0.22	-0.040	0.007	5.81E-09	34
Hypothyroidism	rs4679083	T	0.58	-0.034	0.006	5.80E-09	34
Hypothyroidism	rs4684098	G	0.27	0.085	0.007	1.14E-36	160
Hypothyroidism	rs4690362	A	0.14	-0.093	0.010	2.76E-22	94
Hypothyroidism	rs4742719	G	0.73	-0.055	0.006	4.39E-19	80
Hypothyroidism	rs4743034	G	0.77	0.052	0.007	9.52E-15	60
Hypothyroidism	rs4760332	C	0.69	0.034	0.006	7.18E-09	33
Hypothyroidism	rs4804416	T	0.58	-0.088	0.006	5.27E-55	244
Hypothyroidism	rs4820946	C	0.24	0.047	0.007	2.05E-12	49
Hypothyroidism	rs4844563	A	0.67	-0.034	0.006	6.01E-09	34
Hypothyroidism	rs4915077	T	0.92	-0.238	0.010	5.80E-133	602
Hypothyroidism	rs4933466	A	0.60	0.051	0.006	7.03E-18	74
Hypothyroidism	rs496201	C	0.49	0.031	0.005	2.28E-08	31
Hypothyroidism	rs4963198	G	0.36	-0.033	0.006	1.66E-08	32
Hypothyroidism	rs535850137	G	0.62	-0.059	0.009	5.54E-11	43
Hypothyroidism	rs557444314	A	0.98	-0.197	0.030	8.01E-11	42
Hypothyroidism	rs559734662	T	0.93	0.083	0.014	1.16E-09	37
Hypothyroidism	rs56249713	T	0.58	0.036	0.006	1.33E-10	41
Hypothyroidism	rs564419538	G	0.80	-0.061	0.010	2.49E-09	36
Hypothyroidism	rs56822079	C	0.88	0.050	0.009	2.87E-08	31
Hypothyroidism	rs5750617	T	0.70	-0.042	0.006	8.28E-11	42
Hypothyroidism	rs58288624	T	0.92	0.076	0.013	5.26E-09	34
Hypothyroidism	rs58397833	A	0.67	-0.037	0.006	1.06E-09	37
Hypothyroidism	rs58554773	G	0.82	0.042	0.007	9.49E-09	33
Hypothyroidism	rs587611953	C	0.82	-0.057	0.009	3.34E-11	44
Hypothyroidism	rs59381142	G	0.76	0.046	0.006	5.70E-13	52
Hypothyroidism	rs6036117	A	0.72	0.043	0.007	4.50E-10	39
Hypothyroidism	rs60389750	C	0.68	-0.038	0.007	1.65E-08	32
Hypothyroidism	rs6081210	A	0.20	-0.041	0.007	2.91E-08	31
Hypothyroidism	rs62226440	G	0.71	0.054	0.006	9.05E-18	74
Hypothyroidism	rs62476228	A	0.75	-0.054	0.007	4.03E-13	53
Hypothyroidism	rs62560473	G	0.96	0.214	0.019	1.14E-29	128
Hypothyroidism	rs62621812	G	0.98	-0.105	0.018	8.51E-09	33
Hypothyroidism	rs6415788	G	0.39	0.036	0.006	1.12E-08	33
Hypothyroidism	rs6679677	C	0.90	-0.215	0.009	6.17E-123	556
Hypothyroidism	rs6733824	G	0.44	0.031	0.006	1.26E-08	32

Hypothyroidism	rs67412685	G	0.82	-0.043	0.008	1.08E-08	33
Hypothyroidism	rs6748294	G	0.82	0.044	0.008	1.05E-08	33
Hypothyroidism	rs6751804	C	0.34	-0.049	0.006	1.14E-15	64
Hypothyroidism	rs6755639	A	0.49	-0.044	0.006	2.86E-15	62
Hypothyroidism	rs6892488	C	0.87	-0.051	0.008	6.06E-10	38
Hypothyroidism	rs6919204	C	0.78	-0.042	0.007	3.18E-09	35
Hypothyroidism	rs6923866	T	0.75	0.074	0.007	1.23E-25	110
Hypothyroidism	rs6996585	A	0.59	0.040	0.006	5.14E-12	48
Hypothyroidism	rs7017073	T	0.78	0.043	0.007	8.80E-09	33
Hypothyroidism	rs706778	C	0.59	-0.052	0.005	4.21E-22	93
Hypothyroidism	rs7100297	T	0.41	0.048	0.006	1.32E-17	73
Hypothyroidism	rs7101437	A	0.53	0.041	0.006	2.48E-13	54
Hypothyroidism	rs7128207	G	0.44	-0.056	0.006	5.57E-23	97
Hypothyroidism	rs71311695	T	0.28	0.040	0.007	1.15E-09	37
Hypothyroidism	rs71351111	G	0.65	-0.057	0.006	1.54E-19	82
Hypothyroidism	rs71508903	C	0.80	-0.055	0.007	1.02E-15	64
Hypothyroidism	rs7151424	G	0.55	0.059	0.006	3.33E-24	103
Hypothyroidism	rs7185291	C	0.38	-0.034	0.006	1.53E-08	32
Hypothyroidism	rs7228151	T	0.80	-0.061	0.007	8.71E-18	74
Hypothyroidism	rs7251	C	0.68	0.040	0.006	2.64E-11	44
Hypothyroidism	rs7254729	T	0.42	0.049	0.006	2.30E-17	72
Hypothyroidism	rs72682416	G	0.89	-0.052	0.009	1.89E-09	36
Hypothyroidism	rs72845719	T	0.88	0.119	0.010	8.50E-35	151
Hypothyroidism	rs7298096	G	0.45	0.031	0.006	4.81E-08	30
Hypothyroidism	rs7327740	T	0.73	-0.058	0.006	1.55E-21	91
Hypothyroidism	rs7329077	C	0.90	0.061	0.010	2.43E-10	40
Hypothyroidism	rs73575095	T	0.68	0.103	0.006	4.77E-61	272
Hypothyroidism	rs737308	T	0.28	-0.095	0.007	3.72E-47	208
Hypothyroidism	rs7417283	C	0.21	-0.063	0.008	1.23E-15	64
Hypothyroidism	rs74267890	G	0.98	0.143	0.022	9.17E-11	42
Hypothyroidism	rs742870	T	0.52	0.070	0.006	9.02E-32	138
Hypothyroidism	rs7444385	C	0.60	0.032	0.006	1.29E-08	32
Hypothyroidism	rs74745605	A	0.83	-0.063	0.008	9.73E-17	69
Hypothyroidism	rs748741	A	0.64	-0.063	0.006	3.69E-26	112
Hypothyroidism	rs751171	T	0.67	-0.042	0.006	8.00E-12	47
Hypothyroidism	rs75172178	T	0.56	-0.036	0.006	5.62E-09	34
Hypothyroidism	rs75258843	A	0.98	0.175	0.026	1.44E-11	46
Hypothyroidism	rs75438091	C	0.95	0.107	0.015	6.03E-13	52
Hypothyroidism	rs7568275	G	0.22	0.065	0.007	4.86E-23	98
Hypothyroidism	rs7570971	C	0.62	0.042	0.007	3.13E-09	35
Hypothyroidism	rs75848076	A	0.98	-0.124	0.020	3.41E-10	39
Hypothyroidism	rs75926384	C	0.99	0.287	0.046	3.06E-10	40
Hypothyroidism	rs7617081	A	0.34	0.035	0.006	7.39E-09	33
Hypothyroidism	rs76318082	C	0.90	-0.052	0.009	3.95E-08	30

Hypothyroidism	rs76428106	T	0.99	-0.179	0.027	5.82E-11	43
Hypothyroidism	rs7655915	C	0.81	0.095	0.007	1.96E-39	173
Hypothyroidism	rs77349713	T	0.94	0.078	0.012	1.56E-11	45
Hypothyroidism	rs7752035	T	0.95	-0.076	0.013	4.25E-09	35
Hypothyroidism	rs7754251	G	0.44	-0.069	0.006	1.30E-34	151
Hypothyroidism	rs7772305	A	0.69	-0.058	0.007	5.45E-17	70
Hypothyroidism	rs7799042	G	0.80	0.043	0.007	1.23E-09	37
Hypothyroidism	rs78432262	G	0.94	0.070	0.012	8.08E-09	33
Hypothyroidism	rs78533227	A	0.84	0.046	0.008	6.98E-09	34
Hypothyroidism	rs7853349	A	0.62	-0.146	0.006	4.56E-134	607
Hypothyroidism	rs78622464	C	0.90	0.053	0.010	3.97E-08	30
Hypothyroidism	rs7870475	T	0.52	0.033	0.006	3.56E-08	30
Hypothyroidism	rs7955141	A	0.44	-0.034	0.006	7.87E-09	33
Hypothyroidism	rs7955258	A	0.40	-0.038	0.006	1.63E-10	41
Hypothyroidism	rs7993585	T	0.49	-0.038	0.006	3.92E-11	44
Hypothyroidism	rs8013274	G	0.80	-0.112	0.007	8.99E-56	248
Hypothyroidism	rs8015224	T	0.69	0.037	0.006	5.49E-09	34
Hypothyroidism	rs8107162	T	0.57	0.032	0.006	9.39E-09	33
Hypothyroidism	rs836473	T	0.58	-0.034	0.006	6.67E-09	34
Hypothyroidism	rs855655	T	0.50	-0.037	0.007	2.04E-08	31
Hypothyroidism	rs871852	G	0.50	0.038	0.006	2.31E-11	45
Hypothyroidism	rs881858	G	0.30	-0.108	0.006	1.81E-69	310
Hypothyroidism	rs910634	G	0.51	-0.033	0.006	2.10E-09	36
Hypothyroidism	rs911760	C	0.78	-0.048	0.008	1.69E-08	32
Hypothyroidism	rs9298817	A	0.36	0.046	0.006	9.13E-14	56
Hypothyroidism	rs932036	A	0.70	-0.034	0.006	8.32E-09	33
Hypothyroidism	rs933392	T	0.17	0.063	0.008	6.46E-14	56
Hypothyroidism	rs9368695	C	0.98	0.119	0.021	1.58E-08	32
Hypothyroidism	rs9377117	T	0.72	-0.097	0.007	1.42E-49	219
Hypothyroidism	rs9380229	C	0.71	0.122	0.008	2.57E-48	213
Hypothyroidism	rs9457243	C	0.94	-0.080	0.011	1.06E-12	51
Hypothyroidism	rs9554561	C	0.73	0.042	0.006	8.47E-11	42
Hypothyroidism	rs9729284	C	0.28	-0.036	0.006	1.82E-09	36
Hypothyroidism	rs9821630	A	0.73	0.036	0.007	3.97E-08	30
Hypothyroidism	rs9878655	T	0.56	0.091	0.006	1.32E-50	224
Hypothyroidism	rs992105	C	0.17	-0.049	0.008	1.45E-10	41
Hypothyroidism	rs9926188	C	0.49	0.032	0.006	2.42E-08	31
Hypothyroidism	rs995734	G	0.24	-0.169	0.007	2.05E-121	549
Hyperthyroidism	rs2476601	G	0.89	-0.386	0.054	6.18E-13	52
Hyperthyroidism	rs1977710	G	0.44	0.146	0.022	5.19E-11	43
Hyperthyroidism	rs12612769	C	0.22	0.145	0.025	8.78E-09	33
Hyperthyroidism	rs231779	T	0.47	0.235	0.022	3.61E-26	112
Hyperthyroidism	rs6780858	G	0.47	-0.163	0.023	3.85E-13	53
Hyperthyroidism	rs13136820	T	0.70	-0.136	0.024	2.37E-08	31

Hyperthyroidism	rs73393463	C	0.03	-0.316	0.056	1.51E-08	32
Hyperthyroidism	rs3998799	G	0.40	0.317	0.026	1.21E-33	147
Hyperthyroidism	rs11758670	C	0.12	-0.486	0.035	3.36E-43	190
Hyperthyroidism	rs116749187	T	0.11	0.297	0.035	2.62E-17	72
Hyperthyroidism	rs75324027	G	0.28	0.344	0.024	4.18E-46	204
Hyperthyroidism	rs9357156	C	0.23	0.440	0.025	1.09E-68	307
Hyperthyroidism	rs7754251	C	0.49	0.131	0.023	7.16E-09	33
Hyperthyroidism	rs239933	G	0.48	0.135	0.022	6.73E-10	38
Hyperthyroidism	rs860262	A	0.42	-0.137	0.025	2.11E-08	31
Hyperthyroidism	rs2466028	C	0.36	-0.145	0.024	6.28E-10	38
Hyperthyroidism	rs10821944	T	0.70	-0.157	0.023	1.43E-11	46
Hyperthyroidism	rs4409785	C	0.14	0.203	0.035	6.81E-09	34
Hyperthyroidism	rs7135295	A	0.11	0.237	0.036	3.51E-11	44
Hyperthyroidism	rs28414437	C	0.42	0.287	0.023	1.98E-36	159
Hyperthyroidism	rs9929899	A	0.56	-0.224	0.039	1.31E-08	32
Hyperthyroidism	rs17689159	C	0.30	0.162	0.024	1.55E-11	46
Hyperthyroidism	rs1569723	A	0.71	0.164	0.023	1.62E-12	50
Hyperthyroidism	rs34544259	G	0.40	0.141	0.023	6.75E-10	38

EA, effect allele; EAF, effect allele frequency; TSH, thyroid-stimulating hormone.

Table S6. Association between hypothyroidism and metabolic dysfunction-associated steatotic liver disease (MASLD) histological subtypes by treating simple steatosis as the reference group

	Simple steatosis<br>N=8144	MASH without fibrosis<br>N=1399	Non-cirrhotic fibrosis<br>N=1941	Cirrhosis<br>N=688
Number, %	1.7	3.7	5.3	2.8
· Unadjusted HR (95% CI)	1 (ref.)	2.2 (1.58-3.05)	3.29 (2.52-4.29)	1.48 (0.91-2.42)
· Multivariable adjusted model (95% CI)	1 (ref.)	2.11 (1.42-3.12)	2.72 (2-3.7)	1.17 (0.61-2.24)

Abbreviations: CI, confidence interval; MASH, metabolic dysfunction-associated steatohepatitis; OR, odds ratio.
All models were conditioned on matching factors, including age, sex, biopsy calendar year, and county.
Multivariable adjusted model was adjusted for education, country at birth, metabolic disorders, autoimmune disease, chronic obstructive pulmonary disease, anemia, clinical visit in the past 12 months.

Table S7: Results of subgroup analyses

Stratified Models	Population Controls N=56,831	All MASLD N=12,172	Simple Steatosis N=8144	NASH without fibrosis N=1399	Non-Cirrhotic Fibrosis N=1941	Cirrhosis N=688
Male	1 (ref.)	3.1 (2.02-4.76)	2.76 (1.46-5.21)	1.22 (0.28-5.31)	4.56 (2.04-10.22)	4.15 (0.4-42.78)
Female	1 (ref.)	1.63 (1.3-2.04)	1.23 (0.89-1.71)	2.89 (1.53-5.47)	2.25 (1.49-3.41)	1.16 (0.41-3.29)
Age at index biopsy						
18-39 years	1 (ref.)	5.32 (2.29-12.34)	2.84 (0.76-10.65)	8.18 (0.45-149.8)	24.54 (2.85-210.88)	-
≥40 years	1 (ref.)	1.49 (1.19-1.85)	1.14 (0.83-1.56)	2.24 (1.22-4.11)	2.06 (1.38-3.07)	1.48 (0.55-3.98)

The association was adjusted for age, sex, index calendar year, county, education, metabolic disorders, autoimmune disease, chronic obstructive pulmonary disease, anemia, clinical visit in the past 12 months.

Table S8. Association between hypothyroidism and metabolic dysfunction-associated steatotic liver disease (MASLD) among MASLD cases and their sibling controls

	Sibling controls	All MASLD	Simple Steatosis	MASH without fibrosis	Non-Cirrhotic Fibrosis	Cirrhosis
	N=10,682	N=5478	N=3655	N=632	N=931	N=260
Number, %	1.6	3	1.9	5.1	5.9	3.5
Crude model, OR (95% CI)	1 (ref.)	2.30 (1.73-3.05)	1.93 (1.27-2.92)	2.23 (1.10-4.51)	3.76 (2.13-6.62)	1.52 (0.57-4.04)
Multivariable adjusted model, OR (95% CI)	1 (ref.)	2.08 (1.31-3.31)	1.52 (0.76-3.04)	1.51 (0.35-6.48)	7.94 (1.91-33)	0.81 (0.12-5.65)

Abbreviations: CI, confidence interval; MASH, metabolic dysfunction-associated steatohepatitis; OR, odds ratio.
Crude model was adjusted for age, sex, index calendar year (biopsy year for cases and matching year for sibling controls), and county. Multivariable adjusted model was adjusted for age, sex, index calendar year, county, education, metabolic disorders, autoimmune disease, chronic obstructive pulmonary disease, anemia, clinical visit in the past 12 months.

Table S9. Results of multivariable Mendelian randomization and mediation analyses

Exposure	Adjustment	Beta	SE	P	Mediation	Mediation_LB	Mediation_UB
TSH	None	0.093	0.032	0.004			
	BMI	0.088	0.033	0.007	5.38%	-89.01	99.77
	Type 2 diabetes	0.098	0.033	0.003	-5.38%	-104.81	94.06
	Triglycerides	0.075	0.033	0.024	19.35%	-68.93	107.64
	Systolic blood pressure	0.091	0.033	0.006	2.15%	-73.64	108.05
Hypothyroidism	None	0.072	0.021	0.001			
	BMI	0.070	0.021	0.001	2.78%	-76.95	82.51
	Type 2 diabetes	0.073	0.021	0.000	-1.39%	-82.80	80.02
	Triglycerides	0.065	0.021	0.002	9.72%	-67.29	86.74
	Systolic blood pressure	0.077	0.021	0.000	-6.94%	-75.01	83.35

LB, lower bound of 95% confidence interval; TSH, thyroid-stimulating hormone; UB, upper bound of 95% confidence interval.

Fig. S1. Flow chart of sample selection.

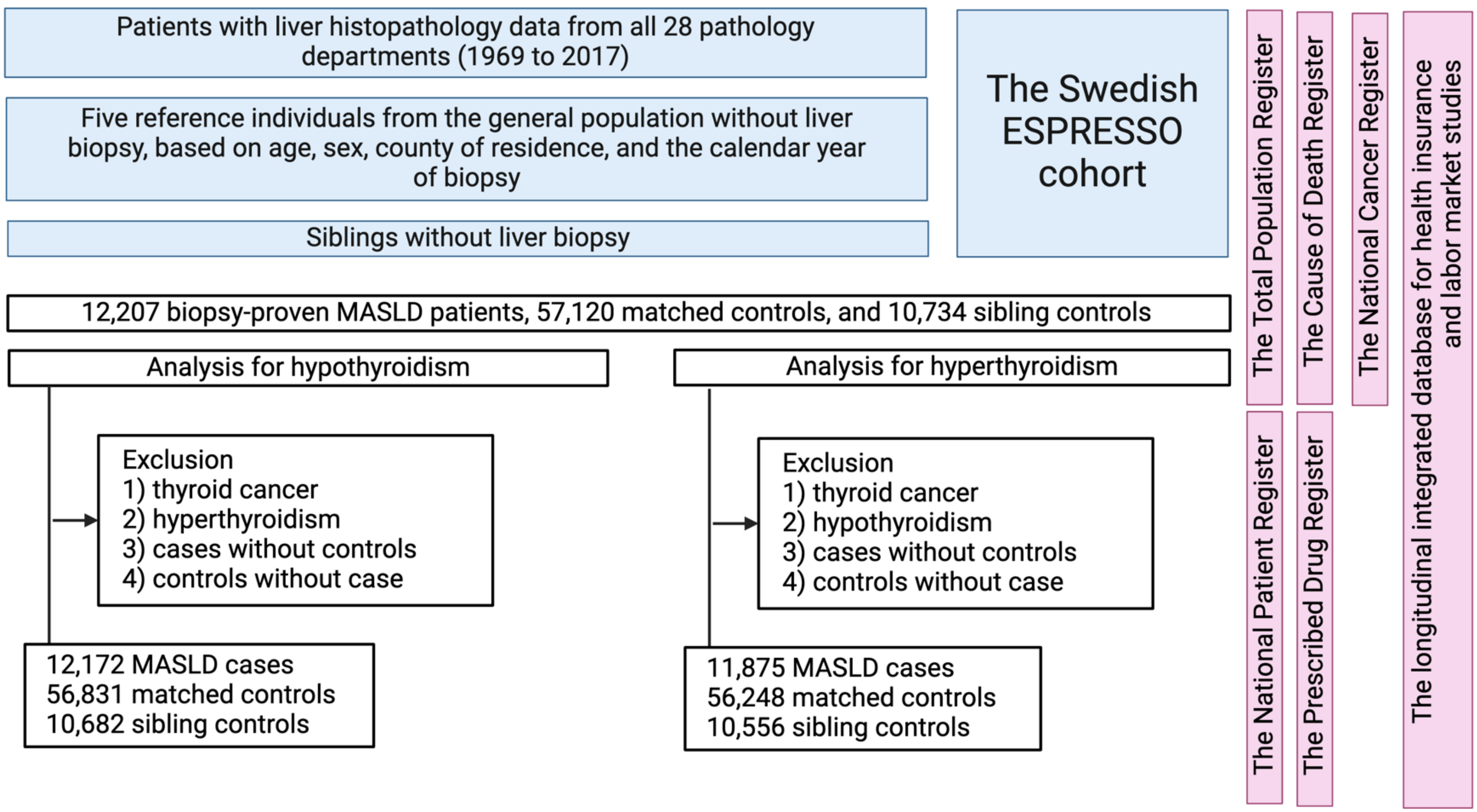

MASLD, metabolic dysfunction-associated steatotic liver disease.

Fig. S2. The directed acyclic graph distinguishing confounding and mediating roles of metabolic disorders in case-control study.

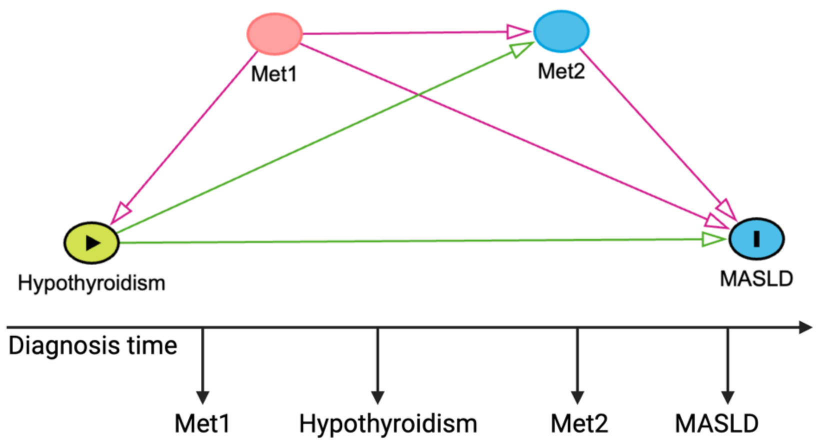

MASLD, metabolic dysfunction-associated steatotic liver disease. Met1 indicates metabolic disorders diagnosed before hypothyroidism and Met2 indicates metabolic disorders diagnosed after hypothyroidism and before MASLD.
